# 14-3-3**ε**–dependent deubiquitination and translocation of NLRP3 activates the inflammasome during sepsis

**DOI:** 10.1172/jci.insight.192970

**Published:** 2026-01-09

**Authors:** Xingyu Li, Siqi Ming, Can Cao, Yating Xu, Jingxian Shu, Ning Tan, Xi Huang, Yongjian Wu

**Affiliations:** 1 Center for Infection and Immunity and Guangdong Provincial Engineering Research Center of Molecular Imaging, the Fifth Affiliated Hospital of Sun Yat-sen University, Zhuhai, Guangdong Province, China.; 2 Department of Laboratory Medicine, Guangdong Provincial Hospital of Chinese Medicine, Zhuhai Hospital, Zhuhai, Guangdong Province, China.; 3 National Clinical Research Center for Infectious Disease, Shenzhen Third People’s Hospital, the Second Affiliated Hospital of Southern University of Science and Technology, Shenzhen, Guangdong Province, China.; 4 Department of Neonatology, the Fifth Affiliated Hospital, Sun Yat-sen University, Zhuhai, Guangdong Province, China.

**Keywords:** Infectious disease, Inflammation, Macrophages

## Abstract

The activation of the NLRP3 inflammasome is a pivotal step in hyperinflammation in sepsis; however, the regulatory mechanisms underlying its activation are not fully understood. In this study, we found that 14-3-3ε facilitates NLRP3 inflammasome activation by enhancing NLRP3 K63 deubiquitination and promoting its translocation to the mitochondria-associated ER membranes (MAMs) for full activation. Mass spectrometry revealed that 14-3-3ε binds to NLRP3 in macrophages during sepsis. Plasma 14-3-3ε levels were elevated in patients with sepsis and were positively associated with disease severity. 14-3-3ε promoted NLRP3 inflammasome activation by facilitating NLRP3 aggregation and NLRP3–ASC assembly. The interaction between 14-3-3ε and NLRP3 was dependent on phosphorylation at the S194 site of NLRP3 NACHT domain. The NLRP3–14-3-3ε interaction promoted K63 deubiquitination and enhanced the translocation of NLRP3 to MAMs, which is necessary for full activation of NLRP3 inflammasome. Furthermore, macrophage-conditional KO of 14-3-3ε or treatment with BV02, a 14-3-3 inhibitor, improved the survival rate and alleviated organ injuries in septic mice. Taken together, our data indicate that 14-3-3ε functions as a positive regulator of the NLRP3 inflammasome and could be a target for sepsis treatment.

## Abstract 2

14-3-3ε promotes NLRP3 inflammasome activation via K63 deubiquitination and MAM translocation, serving as a potential therapeutic target in sepsis.

## Introduction

Sepsis is a life-threatening disease caused by an uncontrolled inflammatory response against pathogens, with the most severe cases characterized by multiple organ dysfunction syndrome (1). An estimated 48.9 million cases of sepsis result in 11 million deaths each year (2), which is a major cause of death in hospitalized patients (3). Because an uncontrolled inflammatory response is the core factor in sepsis pathogenesis, investigating the mechanisms leading to excessive inflammatory responses in sepsis is of great importance.

The macrophage inflammasome, particularly the NLRP3 inflammasome, is a critical factor contributing to the excessive inflammation observed in sepsis (4). Knocking out NLRP3 and treatment with NLRP3 inhibitors, such as MCC950, OLT1177, and CY-09, could considerably increase the survival rates of mice with sepsis (5, 6). NLRP3 is the core component of the inflammasome, and its expression and degradation, together with posttranslational modifications and intracellular distribution, control inflammasome activation (7). Ubiquitination of NLRP3 is one of the major processes controlling inflammasome assembly (8). In resting state, K48-linked ubiquitination mediates proteasomal degradation of NLRP3 and K63-linked ubiquitination maintains NLRP3 in an inactive state, which results in a very low level of NLRP3 and restricts its interaction with ASC (9–11). The activation of the NLRP3 inflammasome is also closely associated with NLRP3 transfer between the mitochondria and Golgi network (12, 13). In the resting state, NLRP3 localizes to ER structures, whereas upon inflammasome activation, both NLRP3 and its adaptor ASC are recruited to mitochondria, where they colocalize with mitochondria-associated ER membranes (MAMs) (13, 14). MAMs are specialized subdomains of the ER that are in close contact with mitochondria and facilitate the transfer of calcium ions (15, 16). The activation of NLRP3 inflammasome requires reactive oxygen species produced by mitochondria, which are closely associated with calcium ion transport (17). MAMs localize adjacent to the Golgi membranes in response to inflammasome activators, where NLRP3 is activated by the phosphorylation of protein kinase D and is released from MAMs (18). Subsequently, the dissociation and recruitment to the Golgi network participates in inflammasome activation (12, 19). However, the molecular basis of these regulatory mechanisms has not been fully elucidated.

In this study, we found that 14-3-3ε, a chaperone protein, promoted inflammasome assembly. In particular, 14-3-3ε interacted with NLRP3 dependent on the phosphorylation of NLRP3 at a S194 site on the NACHT domain. 14-3-3ε promoted K63-linked deubiquitination of NLRP3 and enhanced the translocation of NLRP3 to MAMs via interaction with S194. In vivo experiments showed that macrophage-conditional KO of 14-3-3ε and treatment with a 14-3-3 inhibitor enhanced the survival of mice with sepsis and relieved sepsis-associated organ damage and proinflammatory responses. Collectively, our results reveal the role of 14-3-3ε in NLRP3 inflammasome activation and demonstrate that macrophage-conditional KO of 14-3-3ε or inhibition of 14-3-3 alleviated sepsis-induced organ injury in mice. Thus, 14-3-3ε might be an attractive target for sepsis treatment.

## Results

### 14-3-3ε is a binding protein of NLRP3 inflammasome and is positively correlated with disease severity in sepsis.

**Figure 1:**
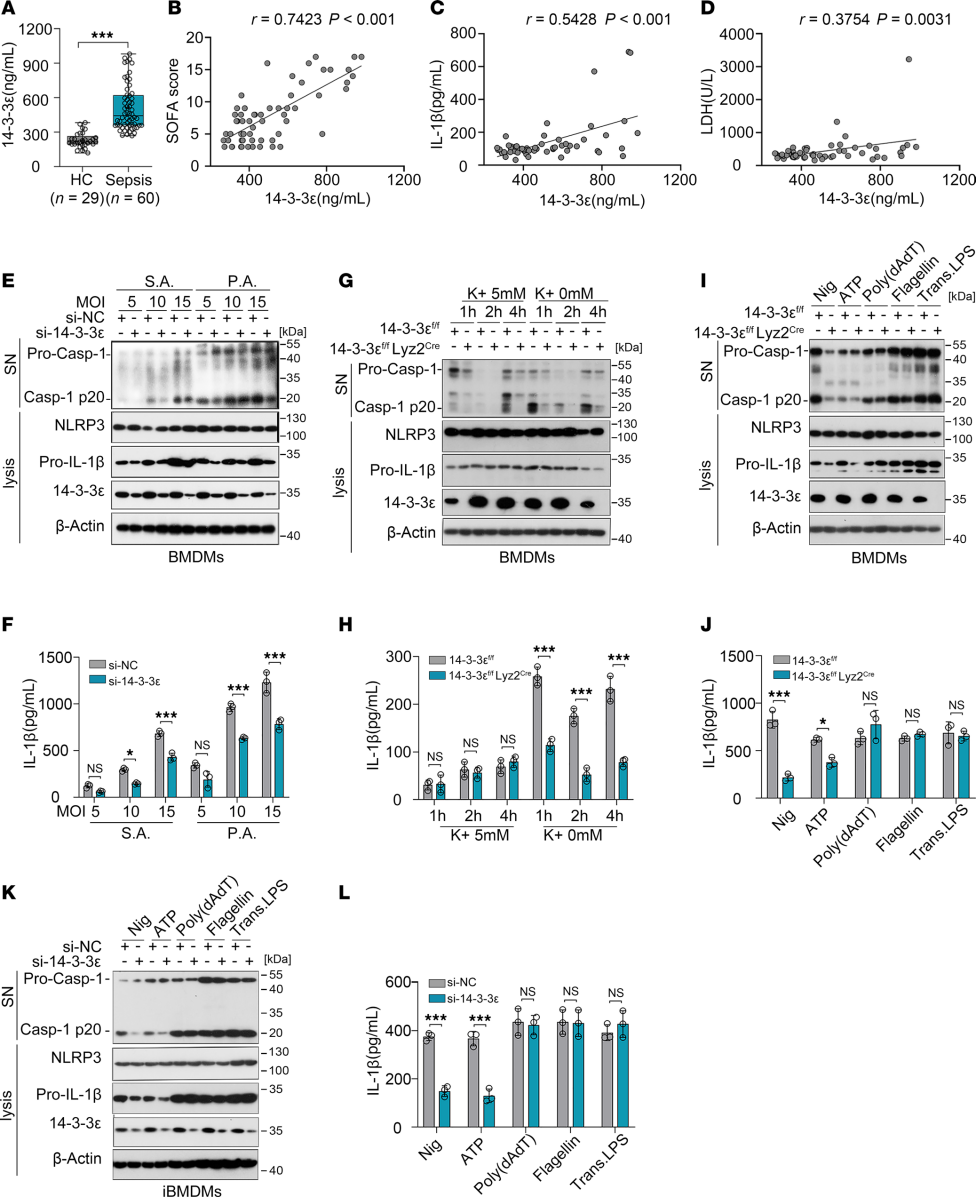
14-3-3ε specifically promotes the activation of NLRP3 inflammasome. (**A**) Levels of 14-3-3ε protein in the plasma of healthy controls (*n* = 29) and patients with sepsis (*n* = 60) determined using ELISA (HC, healthy control). (**B**–**D**) Pearson correlation between plasma14-3-3ε levels and SOFA score (**B**), IL-1β (**C**), or LDH (**D**) levels (*n* = 60). (**E** and **F**) BMDMs were transfected with si-NC or si–14-3-3ε for 24 hours and then infected with *Staphylococcus aureus* (S.A.) or *Pseudomonas aeruginosa* (P.A.) at the indicated MOI for 3 hours. The levels of indicated proteins were detected using western blot analysis (**E**) and the levels of IL-1β (*n* = 3) in culture supernatants were measured using ELISA (**F**). (**G** and **H**) BMDMs from 14-3-3ε^fl/fl^ mice or 14-3-3ε^fl/fl^ Lyz2^Cre^ mice were induced and treated for different time periods with medium containing 0 or 5 mM K^+^. The levels of indicated proteins and the levels of IL-1β (*n* = 3) in culture supernatants were detected. (**I** and **J**) BMDMs from 14-3-3ε^fl/fl^ mice or 14-3-3ε^fl/fl^ Lyz2^Cre^ mice were induced and treated with LPS and stimulated with Nig or ATP or transfected with poly(dAdT), flagellin, or LPS. The expression levels of the indicated proteins (**I**) and the levels of IL-1β (*n* = 3) in culture supernatants were detected (**J**). (**K** and **L**) iBMDMs were transfected with si-NC or si–14-3-3ε and were primed with LPS and stimulated with Nig or ATP or transfected with poly(dAdT), flagellin, or LPS. The expression levels of the indicated proteins (**K**) and the levels of IL-1β (*n* = 3) in culture supernatants were detected (**L**). Data are presented as mean ± SD and were analyzed using 2-tailed *t* test (**A**) and 2-way ANOVA with Tukey’s multiple-comparison test (**F**, **H**, **J**, and **L**). *P < 0.05; **P < 0.01; ***P < 0.001.

To assess the mechanisms by which NLRP3 triggers inflammasome activation in macrophages during sepsis, we identified proteins that directly interact with it in CD11b^+^F4/80^+^ cells sorted from sham-operated or cecal ligation and puncture–challenged (CLP-challenged) mice using mass spectrometry (Supplemental Figure 1A; supplemental material available online with this article; https://doi.org/10.1172/jci.insight.192970DS1). Among the top-20 differential proteins, we noted that the chaperone molecule 14-3-3ε (also named Ywhae) exhibited increased interaction with NLRP3 (Supplemental Table 1). 14-3-3ε is a 246–amino acid protein with molecular weights between 28 and 33 kDa (20). Consistent with this, bands of differential protein that interacted with NLRP3 in the macrophages of sham and septic mice were observed at approximately 30 kDa via silver staining (Supplemental Figure 1B). To determine the association between 14-3-3ε and sepsis, we measured the plasma levels of 14-3-3ε and found that this protein was markedly upregulated in the plasma of patients with sepsis compared with that in healthy controls (Figure 1A). Correlation analysis revealed that plasma 14-3-3ε concentrations were positively correlated with not only sequential organ failure assessment (SOFA) scores (*R* = 0.7423), but also with IL-1β (*R* = 0.5428) and lactate dehydrogenase (LDH; *R* = 0.3754) plasma levels (Figure 1, B–D), indicating that 14-3-3ε correlated with inflammation and severity of sepsis. To investigate changes in 14-3-3ε expression within peripheral blood monocytes and neutrophils from patients with sepsis, we performed flow cytometry to analyze 14-3-3ε levels in CD15^+^CD16^+^ neutrophils and CD14^+^ monocytes. No statistically significant differences were found in either the positivity rate or mean fluorescence intensity (MFI) of 14-3-3ε in peripheral monocytes and neutrophils between patients with sepsis and healthy controls (Supplemental Figure 1, C–F). However, Western blot analysis revealed a substantial increase in 14-3-3ε levels in the supernatant of bone marrow–derived macrophages (BMDMs) following NLRP3 inflammasome activation induced by LPS and nigericin (Nig) (Supplemental Figure 2A). These findings suggest that the elevated plasma 14-3-3ε levels in patients with sepsis may originate from pyroptotic cell. Because levels of cleaved caspase 1 p20 (Casp-1 p20) and release of mature IL-1β are indicators of inflammasome activation (21), we further evaluated these 2 indicators to detect the regulatory effect of 14-3-3ε on NLRP3 inflammasome activation. Here, we transfected BMDMs with 14-3-3ε or negative control siRNA and infected them with *Staphylococcus aureus* (S.A.) or *Pseudomonas aeruginosa* (P.A.), the 2 commonly reported bacteria in sepsis. Using immunoblotting to detect the p20 fragment and ELISA to detect the IL-1β levels in cell-culture supernatants, we observed that the levels of Casp-1 p20 and IL-1β were increased at MOI of 10 and 15, and both levels decreased when 14-3-3ε was silenced (Figure 1, E and F). K^+^ efflux is an essential step in the activation of the NLRP3 inflammasome (22). When BMDMs from 14-3-3ε^fl/fl^ mice or 14-3-3ε^fl/fl^ Lyz2^Cre^ mice (macrophage-conditional KO of 14-3-3ε) were cultured with medium containing or not containing K^+^ for the indicated time, the levels of Casp-1 p20 fragment and IL-1β in cell-culture supernatants decreased in 14-3-3ε–KO BMDMs (Figure 1, G and H). Next, we investigated whether 14-3-3ε affects other inflammasomes. BMDMs from 14-3-3ε^fl/fl^ mice or 14-3-3ε^fl/fl^ Lyz2^Cre^ mice were preprimed with LPS and subsequently treated with Nig and ATP (NLRP3 inflammasome activators), or transfected with poly(dAdT) (an AIM2 inflammasome activator), flagellin (an NLRC4 inflammasome activator), or LPS (a caspase 11 inflammasome activator). The Western blot analysis and ELISA results show that 14-3-3ε KO only downregulated Casp-1 p20 and IL-1β levels in cells treated with NLRP3 inflammasome activators but had no effect in groups treated with other activators (Figure 1, I and J). The results in iBMDMs also revealed that 14-3-3ε silencing only inhibited NLRP3 inflammasome activation but did not affect the activation of AIM2, NLRC4, or caspase 11 inflammasomes (Figure 1, K and L). Since 14-3-3ε could regulate cell death or autophagy through interacting with the Bcl-2–associated death promoter (BAD) or protein phosphatase 1B (PPM1B) (23, 24), we then determined the apoptosis and autophagy in 14-3-3ε–KO BMDM upon LPS and Nig treatment. 14-3-3ε–KO did not affect LPS- and Nig-induced apoptosis (Supplemental Figure 3, A and B) or expression of microtubule-associated protein 1 light chain 3-II (LC3-II) (Supplemental Figure 3C). Taken together, these findings indicate that 14-3-3ε was associated with sepsis and promoted the activation of NLRP3 inflammasome.

### 14-3-3ε facilitates the assembly of the NLRP3 inflammasome complex.

**Figure 2:**
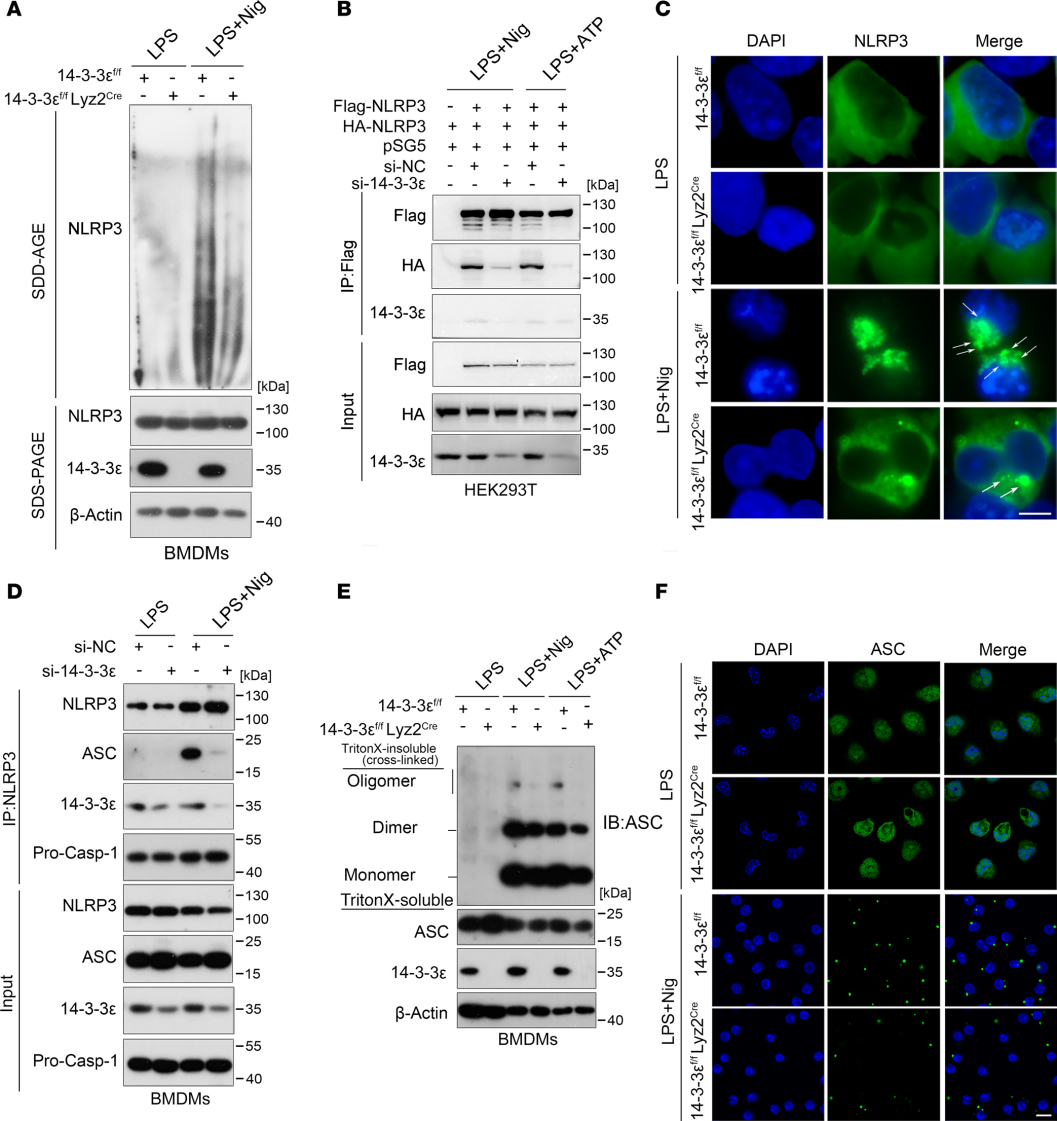
14-3-3ε facilitates the assembly of the NLRP3 inflammasome. (**A**) BMDMs from 14-3-3ε^fl/fl^ or 14-3-3ε^fl/fl^ Lyz2^Cre^ mice were stimulated with LPS alone or with LPS and Nig; the NLRP3 oligomerization was then fractionated by SDD-AGE, and detected via western blot analysis. (**B**) Co-IP analysis of the interaction between FLAG tagged- and HA tagged-NLRP3 in HEK 293T cells transfected with si-NC or si–14-3-3ε and stimulated with LPS- and Nig or ATP. (**C**) BMDMs from14-3-3ε^fl/fl^ or 14-3-3ε^fl/fl^ Lyz2^Cre^ mice were induced and treated with LPS and Nig, and NLRP3 was visualized using immunofluorescence staining and imaging using confocal microscopy. Scale bar:10 μm. (**D**) BMDMs were transfected with si-NC or si–14-3-3ε, and then NLRP3–ASC interactions in LPS and Nig-stimulated BMDMs were detected using co-IP analysis. (**E** and **F**) BMDMs from 14-3-3ε^fl/fl^ mice or 14-3-3ε^fl/fl^ Lyz2^Cre^ mice were primed with LPS and stimulated with Nig or ATP. (**E**) ASC oligomerization was crosslinked by DSS and detected using western blot analysis. (**F**) ASC specks were detected using immunofluorescence staining with an anti-ASC antibody followed by confocal microscopy. Scale bar:10 μm.

The activation of NLRP3 inflammasome is involved in priming and activation phases, which include transcriptional upregulation of inflammasome components and formation of protein complex, respectively (25). Because the expression of inflammasome component genes is dependent on the activation of NF-κB/MAPKs signaling pathways (26), we first detected the role of 14-3-3ε in the regulation of NF-κB/MAPKs signaling pathways. Western blot analysis results show that LPS treatment remarkably increased ERK1/2, JNK, and p38 phosphorylation but KO of 14-3-3ε had no obvious effects on these phosphorylation (Supplemental Figure 4A). Consistent with this, 14-3-3ε KO did not affect the RNA expression levels of *nlrp3*, *il1b*, *casp1*, or *asc* (Supplemental Figure 4B). Next, we validated the regulatory effect of 14-3-3ε on the inflammasome components at the protein level and found that silencing 14-3-3ε did not influence the protein levels of Pro–casp-1 and NLRP3 but decreased the expression of the Pro–IL-1β protein (Supplemental Figure 4, C–E). Moreover, the reduction in Pro–IL-1β protein levels after 14-3-3ε silencing or KO was partly attenuated by treatment with the proteasome inhibitor MG132 (Supplemental Figure 4, F and G), suggesting that 14-3-3ε may preserve the protein levels of Pro–IL-1β by affecting its proteasomal degradation. Next, we investigated whether 14-3-3ε participates in the assembly of NLRP3 and ASC. The effect of 14-3-3ε on the endogenous NLRP3 oligomerization was confirmed using semidenaturing detergent agarose gel electrophoresis (SDD-AGE), a method for detecting large protein oligomers. The oligomerization of NLRP3 was markedly decreased in 14-3-3ε–deficient BMDMs (Figure 2A). FLAG- and HA-tagged NLRP3 plasmids and 14-3-3ε siRNA were transfected into HEK-293T cells. The NLRP3-NLRP3 interaction was found to be considerably weakened when 14-3-3ε expression was silenced (Figure 2B). Next, we examined the NLRP3-NLRP3 interaction by immunofluorescence staining of BMDMs from 14-3-3ε^fl/fl^ mice or 14-3-3ε^fl/fl^ Lyz2^Cre^ mice subjected to LPS and Nig treatment. We found that 14-3-3ε KO suppressed the formation of the NLRP3 complex (Figure 2C). During NLRP3 inflammasome activation upon treatment of BMDMs with LPS and Nig, 14-3-3ε silencing notably weakened the NLRP3-ASC interaction (Figure 2D) and 14-3-3ε KO suppressed the formation of ASC oligomerization (Figure 2E). Consistent with this, 14-3-3ε KO decreased ASC-speck formation in BMDMs following LPS and Nig treatment, as observed with immunofluorescence staining (Figure 2F). Together, these results indicate that 14-3-3ε mainly promoted NLRP3 inflammasome assembly but did not induce transcriptional upregulation of the inflammasome components.

### NACHT domain of NLRP3 is necessary for 14-3-3ε interaction.

**Figure 3:**
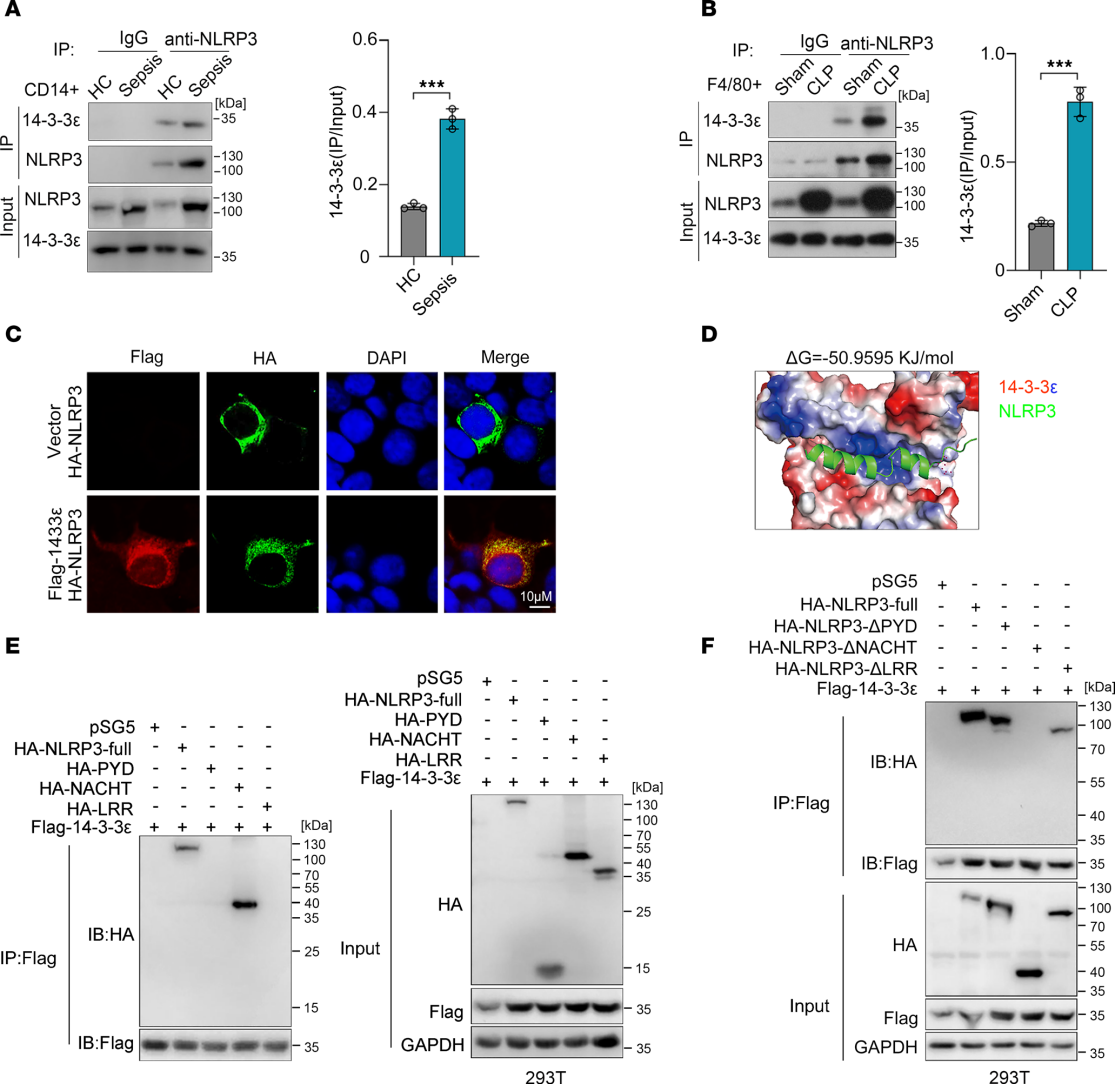
14-3-3ε interacts with the NACHT domain of NLRP3. (**A**) Co-IP analysis of the interaction between NLRP3 and 14-3-3ε in CD14^+^ cells sorted from the peripheral blood of healthy controls and patients with sepsis. The gray-scale values of 14-3-3ε protein bands were measured using the ImageJ software (NIH). (**B**) Co-IP analysis of the interaction between NLRP3 and 14-3-3ε in CD11b^+^F4/80^+^ cells sorted from the tissue of mouse subjected to sham or CLP surgery. The gray-scale values of 14-3-3ε protein bands were measured using the ImageJ software. (**C**) Confocal microscopy images of HEK-293T cells cotransfected with HA-tagged NLRP3 and FLAG-tagged 14-3-3ε plasmids. Scale bar: 10 μm. (**D**) Optimal conformation of the interaction between 14-3-3ε and NLRP3 predicted using AlphaFold3; molecular visualization was carried out using the PyMOL software. 14-3-3ε is presented in the surface potential mode and NLRP3 is presented in the stick mode. (**E**) Co-IP analysis of the interaction between 14-3-3ε and the different domains of NLRP3 in HEK-293T cells cotransfected with FLAG-tagged 14-3-3ε plasmid and plasmids containing HA-tagged full-length NLRP3 or the PYD, NACHT, or LRR domain. (**F**) Co-IP analysis of the interaction between FLAG-tagged 14-3-3ε and HA-tagged full-length NLRP3 or NLRP3 lacking the PYD (ΔPYD), NACHT (ΔNACHT), or LRR (ΔLRR) domain in HEK-293T cells. Data are presented as mean ± SD and were analyzed using 2-tailed *t* test (**A** and **B**). ***P < 0.001.

To further confirm the interaction between NLRP3 and 14-3-3ε, coimmunoprecipitation (co-IP) was performed in human monocytes and mouse macrophages. Compared with those in monocytes sorted from healthy controls, increased NLRP3 expression levels and an enhanced interaction between NLRP3 and 14-3-3ε were observed in CD14^+^ monocytes from the peripheral blood of patients with sepsis (Figure 3A). Consistent with this, the NLRP3–14-3-3ε interaction was also augmented in CD11b^+^F4/80^+^ macrophages from septic mice (Figure 3B). Fluorescence microscopy showed colocalization of exogenously expressed NLRP3 (with HA tag) and 14-3-3ε (with FLAG tag) in the cytoplasm of HEK-293T cells (Figure 3C). In the best docking conformation of NLRP3 and 14-3-3ε, the corresponding binding free energy was −50.9595 KJ/mol, indicating strong binding potential for NLRP3 for 14-3-3ε binding (Figure 3D). NLRP3 consists of 3 domains: a pyrin domain (PYD) at the N-terminus, a central NACHT domain, and a leucine-rich repeat domain (LRR) at the C-terminus (7). To explore the specific domain of NLRP3 interacted by 14-3-3ε, we detected the interaction between 14-3-3ε and the 3 functional domains of NLRP3. Co-IP assay showed that 14-3-3ε specifically copurified with the NACHT domain but not with the PYD or LRR domain (Figure 3E). When the NACHT domain of NLRP3 was deleted, the NLRP3–14-3-3ε interaction was lost (Figure 3F). These results indicated that 14-3-3ε directly interacted with NLRP3 and that the major domain in NLRP3 mediating its interaction with 14-3-3ε is the NACHT domain.

### Interaction of 14-3-3ε with NLRP3 is dependent on the S194 phosphorylation of NLRP3.

**Figure 4:**
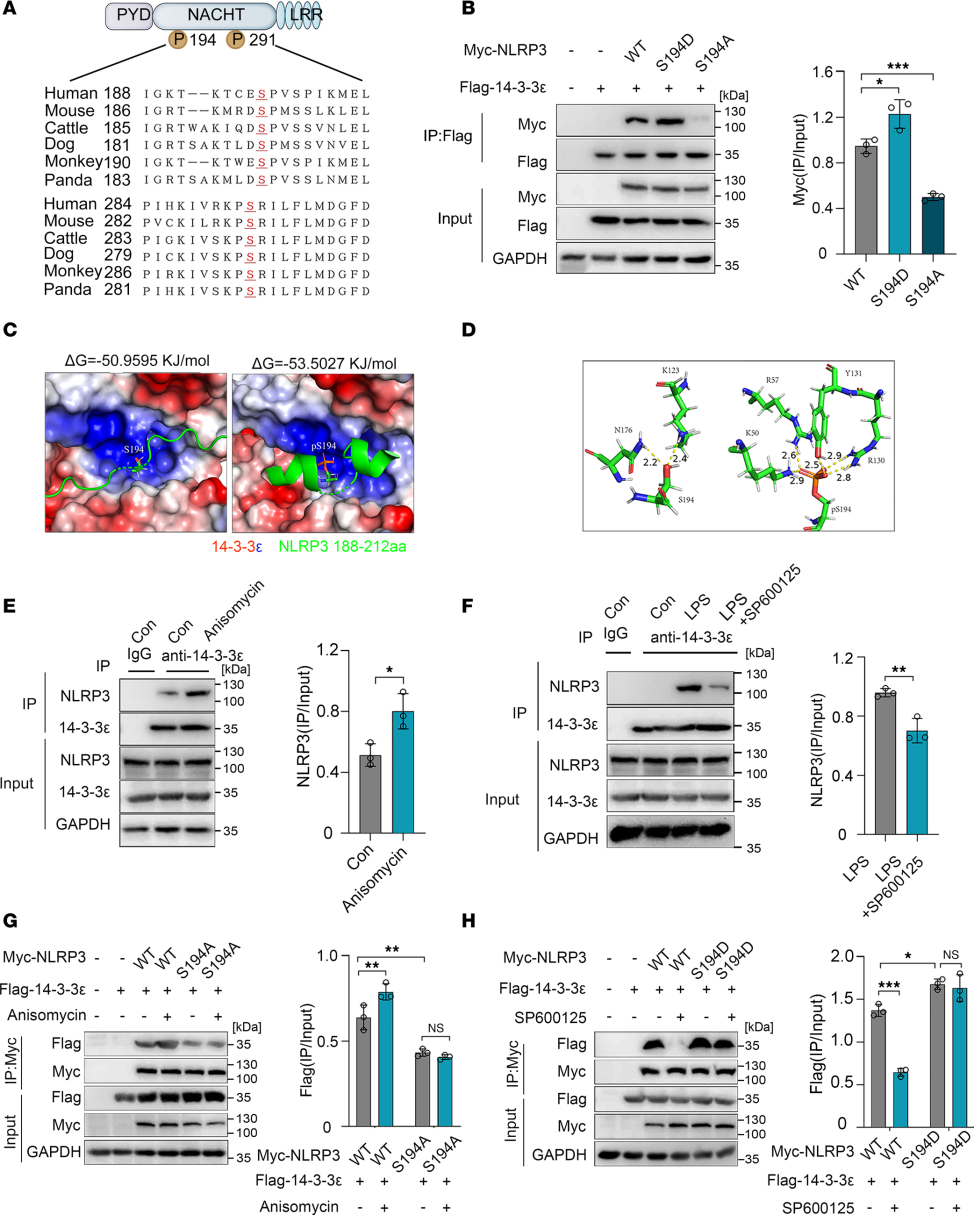
14-3-3ε–NLRP3 interaction depends on NLRP3 phosphorylation at S194 position. (**A**) Alignment of NLRP3 orthologs. S194 and S291 are marked in red. (**B**) Co-IP analysis of the interaction between 14-3-3ε and S194A or S194D NLRP3 protein. The gray-scale values of Myc-tagged NLRP3 protein bands were measured using the ImageJ software (*n* = 3). (**C**) Optimal conformation of the interaction between 14-3-3ε and NLRP3 188–212 amino acid sequence (without or with phosphorylation on S194) predicted using AlphaFold3; molecular visualization was carried out using the PyMOL software. (**D**) Residues in the protein complex subunits that form hydrogen bonds (yellow) presented in the stick mode. (**E**) Co-IP analysis of the endogenous interaction between 14-3-3ε and NLRP3 in iBMDMs with or without anisomycin treatment. The gray-scale values of NLRP3 protein bands were measured using the ImageJ software (*n* = 3). (**F**) iBMDMs were pretreated with SP600125 and then treated with LPS. Co-IP was performed and the gray-scale values of NLRP3 protein bands were measured using the ImageJ software (*n* = 3). (**G**) HEK-293T cells were transfected with FLAG-tagged 14-3-3ε and Myc-tagged NLRP3 plasmids with S194A mutation and then treated with anisomycin. The interaction between 14-3-3ε and NLRP3 was then detected using co-IP analysis. The gray-scale values of NLRP3 protein bands were measured using the ImageJ software (*n* = 3). (**H**) HEK-293T cells were transfected with FLAG-tagged 14-3-3ε and Myc-tagged NLRP3 plasmids with S194D mutation, before being treated with SP600125. The interaction between 14-3-3ε and NLRP3 was detected using co-IP analysis. The gray-scale values were measured using the ImageJ software (*n* = 3). Data are presented as mean ± SD and were analyzed using 1-way ANOVA with Tukey’s multiple-comparison test (**B**), 2-tailed *t* test (**E** and **F**), and 2-way ANOVA with Tukey’s multiple-comparison test (**G** and **H**). *P < 0.05; **P < 0.01; ***P < 0.001.

The interaction of 14-3-3ε with its chaperone proteins is dependent on the phosphorylation of serine residues (27). As 14-3-3ε interacts with NLRP3 through the NACHT domain, we observed 2 known serine phosphorylation sites, serine 194 (S194) and serine 291 (S291) on the NACHT domain of NLRP3 (Figure 4A). To clarify if the interaction between 14-3-3ε and NLRP3 is dependent on the phosphorylation of S194 or S291, the serine (S) was mutated to alanine (A) to abolish phosphorylation, or it was mutated to aspartate (D) to mimic phosphorylation (28–30). Co-IP assays in HEK-293T cells showed that the phosphomimetic mutation S194D of NLRP3 potentiated its interaction with 14-3-3ε, whereas the unphosphorylated mutation S194A weakened this interaction (Figure 4B). However, neither the phosphomimetic nor the unphosphorylated mutation on S291 affected the NLRP3-14-3-3ε interaction (Supplemental Figure 5A). The structure prediction of the protein complex showed that phosphorylation at S194 had a low binding free energy (Figure 4C). Using molecular docking to analyze the intermolecular interactions between NLRP3 and 14-3-3ε, we found that S194 phosphorylation greatly enhanced the binding affinity of NLRP3 for 14-3-3ε by increasing hydrophilic forces (Figure 4D). Phosphorylation of NLRP3 at S194 specifically depends on the activation of c-Jun N-terminal kinase (JNK). The JNK activator anisomycin promotes, whereas the JNK-specific inhibitor SP600125 inhibits, the phosphorylation of NLRP3 at S194 (30). Here, we found that anisomycin treatment augmented the 14-3-3ε–NLRP3 interaction (Figure 4E) and SP600125 treatment weakened the interaction in iBMDMs (Figure 4F). However, when S194 phosphorylation was abolished by mutating S194 to alanine, the strengthened interaction caused by anisomycin treatment was suppressed (Figure 4G). Corroborating this, the inhibitory effects of SP600125 on the 14-3-3ε–NLRP3 interaction were almost abolished when S194 was mutated to aspartate (Figure 4H). Together, these data indicate that the phosphorylation of S194 site of NLRP3 is critical for its interaction with 14-3-3ε.

### 14-3-3ε triggers NLRP3 K63–linked deubiquitination via S194 phosphorylation of NLRP3.

**Figure 5:**
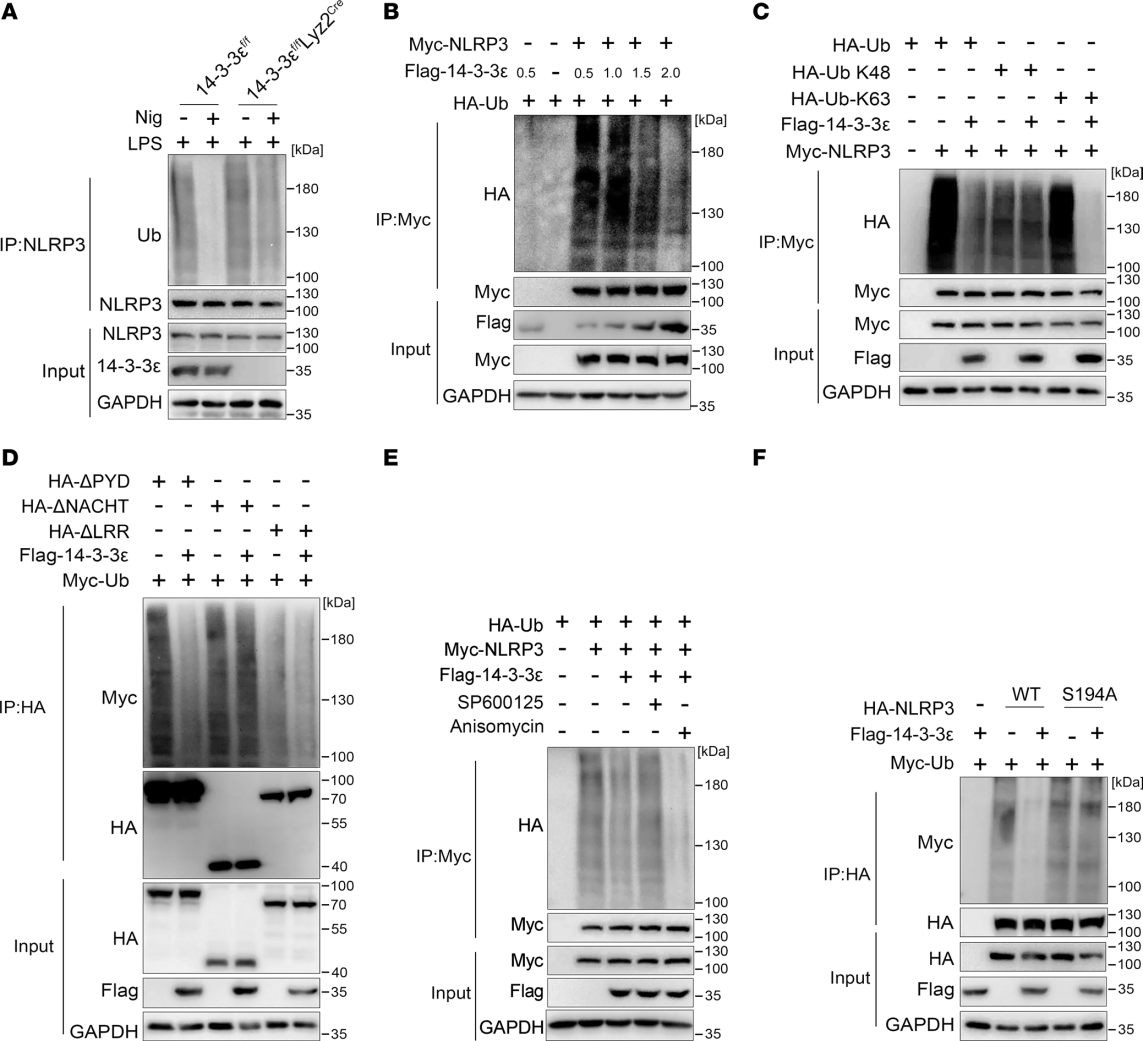
14-3-3ε promotes NLRP3 K63–linked deubiquitination via the phosphorylation of NLRP3 at S194. (**A**) BMDMs from 14-3-3ε^fl/fl^ mice or 14-3-3ε^fl/fl^ Lyz2^Cre^ mice were treated with LPS alone or cotreated with LPS and Nig. Ubiquitination levels of NLRP3 were detected using co-IP analysis. (**B**) HEK-293T cells were cotransfected with Myc-tagged NLRP3 and HA-tagged Ub plasmids together with different amounts of FLAG-tagged 14-3-3ε plasmids (μg). Myc-NLRP3 was then immunoprecipitated with anti-Myc antibody to detect the ubiquitination levels of NLRP3. (**C**) Co-IP analysis was performed to detect the K48 or K63 ubiquitinated-NLRP3 in MG132-treated HEK-293T cells, which were transfected with FLAG-tagged 14-3-3ε, Myc-NLRP3, and HA-tagged K48/K63-linked Ub plasmids. (**D**) HEK-293T cells were cotransfected with FLAG-tagged 14-3-3ε, Myc-tagged Ub, and HA-tagged ΔPYD, ΔNACHT, and ΔLRR plasmids, after which they were treated with MG132 and the ubiquitination levels of NLRP3 truncations were detected using co-IP analysis. (**E**) HEK-293T cells were cotransfected with FLAG-tagged 14-3-3ε, Myc-tagged NLRP3, and HA-tagged Ub plasmids. The cells were then treated with anisomycin or SP100625 and subsequently with MG132. Whole cell lysates were prepared to detect the ubiquitination levels of NLRP3 using co-IP analysis. (**F**) HEK-293T cells were cotransfected with FLAG-tagged 14-3-3ε, Myc-tagged Ub, and HA-tagged WT or S194A mutated NLRP3 plasmids and treated with MG132. The ubiquitination levels of NLRP3 were detected using co-IP analysis.

As a molecular chaperone, 14-3-3ε associates with and regulates the ubiquitination of its client proteins (31, 32). Moreover, NLRP3 deubiquitination is necessary for the assembly and activation of the NLRP3 inflammasome (33). We, therefore, checked whether 14-3-3ε influenced the deubiquitination of NLRP3. The co-IP revealed that the ubiquitination levels of NLRP3 were decreased upon LPS and Nig treatment, but when 14-3-3ε was knocked out, the reduction in NLRP3 ubiquitination was inhibited (Figure 5A). We also observed that when 14-3-3ε was exogenously expressed in HEK-293T cells, increased NLRP3 deubiquitination was associated with increased expression of 14-3-3ε (Figure 5B). K48 and K63 are the 2 most common sites of ubiquitination in NLRP3 (34). To determine whether the enhancement of NLRP3 deubiquitination by 14-3-3ε was K48 or K63 linked, we transfected HA-tagged ubiquitin (Ub) K48 and K63 plasmids, with FLAG-tagged 14-3-3ε and Myc-tagged NLRP3 plasmids into HEK-293T cells and assessed the Myc-immunoprecipitates for ubiquitinated NLRP3. The results show that 14-3-3ε overexpression enhanced the total and K63-linked deubiquitination, instead of K48-linked deubiquitination of NLRP3 (Figure 5C). NLRP3 ubiquitination on the sites in different domains is a dynamic process coordinated by ubiquitylating and deubiquitylating enzymes. Here, plasmids expressing HA-tagged domain deletion mutants were transfected into HEK-293T cells with plasmids expressing Myc-tagged Ub and FLAG-tagged 14-3-3ε, and the results revealed that deletion of the NACHT or LRR domain diminished the deubiquitination induced by 14-3-3ε overexpression, whereas deletion of the PYD retained the 14-3-3ε–induced deubiquitination (Figure 5D). Several E3 Ub ligases and deubiquitylases have been reported to regulate NLRP3 via the sites on NACHT or LRR domain (35). To identify the Ub-conjugating E3 enzyme that may be contributing to 14-3-3ε–related deubiquitination of NLRP3, we examined the mRNA levels of ubiquitylating (RNF 125, Pellino2, FBXL2, Cbl-b, CUL1, March7, and ARIK2) and deubiquitylating (USP7, USP47, and BRCC3) enzymes that were associated with NACHT or LRR domain in LPS- and Nig-treated BMDMs from 14-3-3ε^fl/fl^ or 14-3-3ε^fl/fl^ Lyz2^Cre^ mice (34). We found that the treatment induced the downregulation of the ubiquitylating enzyme RNF125, and 14-3-3ε KO counteracted this inhibition (Supplemental Figure 6, A and B), which indicated that RNF125 might be one of the key enzymes involved in 14-3-3ε–induced NLRP3 deubiquitination. Because the ubiquitination of NLRP3 depended on its phosphorylation and 14-3-3ε interacted with NLRP3 through the phosphorylation on S194 of NLRP3, we detected whether S194 phosphorylation affected 14-3-3ε–induced NLRP3 ubiquitination. The co-IP results show that anisomycin and SP600125 promoted and inhibited NLRP3 deubiquitination, respectively (Figure 5E). However, when S194 of NLRP3 was mutated to S194A, the 14-3-3ε–induced NLRP3 deubiquitination was abolished (Figure 5F). Together, these results indicate that 14-3-3ε promoted NLRP3 K63 deubiquitination through interacting with S194 phosphorylated site of NLRP3.

### 14-3-3ε promotes NLRP3 trafficking to MAMs.

**Figure 6:**
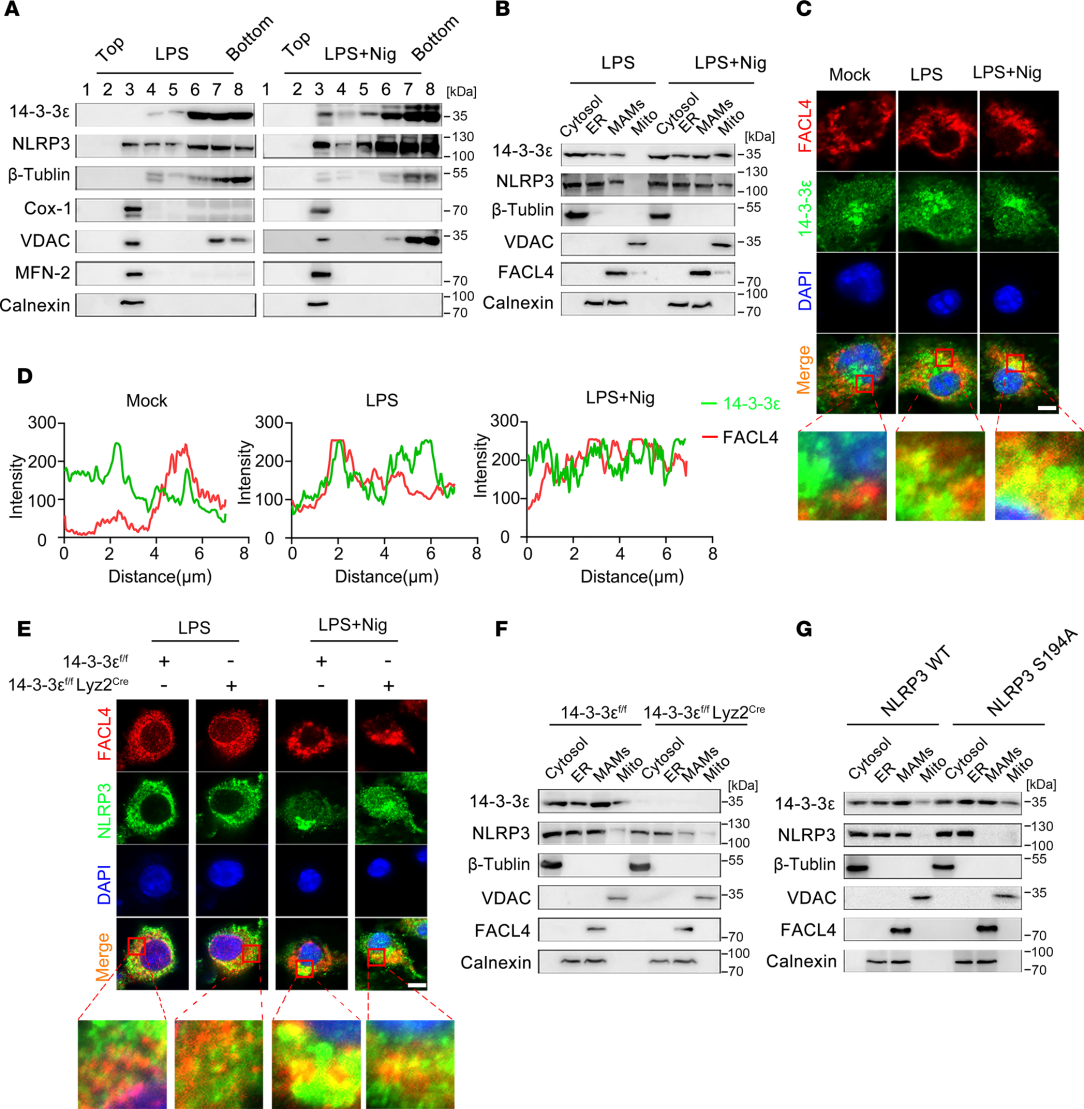
14-3-3ε promotes the translocation of NLRP3 proteins to mitochondria-associated membranes (MAMs). (**A**) iBMDMs were treated with LPS alone or co-treated with LPS and Nig. The cell lysate was used to isolate the membrane fractions via membrane flotation. The membrane markers (β-tubulin for cytosol, Cox-1 for inner mitochondrial membrane [mito], VDAC for outer mito, MFN-2 for MAMs), and calnexin for ER, 14-3-3ε, and NLRP3 in different fractions were probed with the indicated antibodies using Western blot. (**B**) iBMDMs were treated with LPS alone or cotreated with LPS and Nig. The cytosol, ER, MAMs, and mito were isolated using organelle isolation, and the levels of 14-3-3ε, NLRP3, and membrane markers in different fractions were detected using western blot analysis. (**C**) BMDMs were treated with LPS alone or cotreated with LPS and Nig, stained for FACL4 (red), and 14-3-3ε (green) and were observed by confocal microscopy. Scale bar: 5 μm. (**D**) The fluorescence intensity along the diagonal line from top left corner to the lower right corner of the bottom images in **C** were analyzed by ImageJ and were shown in the graphs. (**E**) BMDMs from 14-3-3ε^fl/fl^ or 14-3-3ε^fl/fl^ Lyz2^Cre^ mice were induced and treated with LPS alone or cotreated with LPS and Nig. NLRP3 (red) and FACL4 (green) were stained and observed using confocal microscopy. Scale bar: 5 μm. (**F**) BMDMs from 14-3-3ε^fl/fl^ or 14-3-3ε^fl/fl^ Lyz2^Cre^ mice were induced and treated with LPS and Nig. The cytosol, ER, MAMs, and mitochondria were isolated, and the protein levels of 14-3-3ε, NLRP3, and membrane markers were detected using western blot analysis. (**G**) HEK-293T cells were transfected with WT of S194A mutated Myc-tagged NLRP3 plasmids and treated with LPS and Nig. The cytosol, ER, MAMs, and mitochondria were isolated and detected using Western blot.

Given that 14-3-3ε regulates intracellular localization of the client proteins and intracellular localization of NLRP3 affects its activation, we subsequently investigated whether 14-3-3ε affects subcellular redistribution of NLRP3. Thus, the organellar membranes and cytoplasmic fractions in LPS-treated or LPS and Nig-treated iBMDMs were separated by membrane flotation, and 8 fractions collected from top to bottom were used for Western blot analysis. We found that membrane organelles, including the mitochondrial membrane, ER and MAMs, were mostly present in fraction 3, whereas cytoplasmic proteins were present mainly in fractions 6, 7, and 8 (Figure 6A). 14-3-3ε and NLRP3 were mainly detected in the cytoplasm when treated with LPS alone but were transferred to organellar membrane (fraction 3) upon LPS- and Nig treatment (Figure 6A). We next isolated cytosol, ER, MAMs, and mitochondria and found that 14-3-3ε and NLRP3 were mainly transferred to MAMs and mitochondria (Figure 6B). Immunofluorescence staining revealed that the colocalization of 14-3-3ε and fatty acid CoA ligase 4 (FACL4, a MAMs marker) increased in the LPS and Nig group (Figure 6, C and D). We next sought to decipher whether the translocation of NLRP3 to MAMs is 14-3-3ε dependent. BMDMs from 14-3-3ε^fl/fl^ or 14-3-3ε^fl/fl^ Lyz2^Cre^ mice were induced with LPS and Nig to activate the NLRP3 inflammasome, and the translocation of NLRP3 to MAMs was examined using immunofluorescence by staining with antibodies against NLRP3 and FACL4. NLRP3 was located in the cytoplasm of BMDMs when treated with LPS alone, and when stimulated with LPS and Nig, it colocalizes with FACL4. However, when 14-3-3ε was knocked out, the colocalization of NLRP3 and FACL4 was decreased (Figure 6E). Decreased level of NLRP3 in MAMs was also confirmed in 14-3-3ε KO BMDMs (Figure 6F). We next determined if the translocation of NLRP3 to MAMs was dependent on the S194 by expressing S194 mutated NLRP3 in HEK-293T cells. As evident from the Western blot analysis results, when S194 was mutated to S194A, the NLRP3 translocation to MAMs was decreased (Figure 6G). These results indicate that 14-3-3ε promoted the translocation of NLRP3 to MAMs, and the translocation of NLRP3 to MAMs was associated with S194 phosphorylation of NLRP3.

### Macrophage-conditional KO of 14-3-3ε protects mice from polymicrobial sepsis.

**Figure 7:**
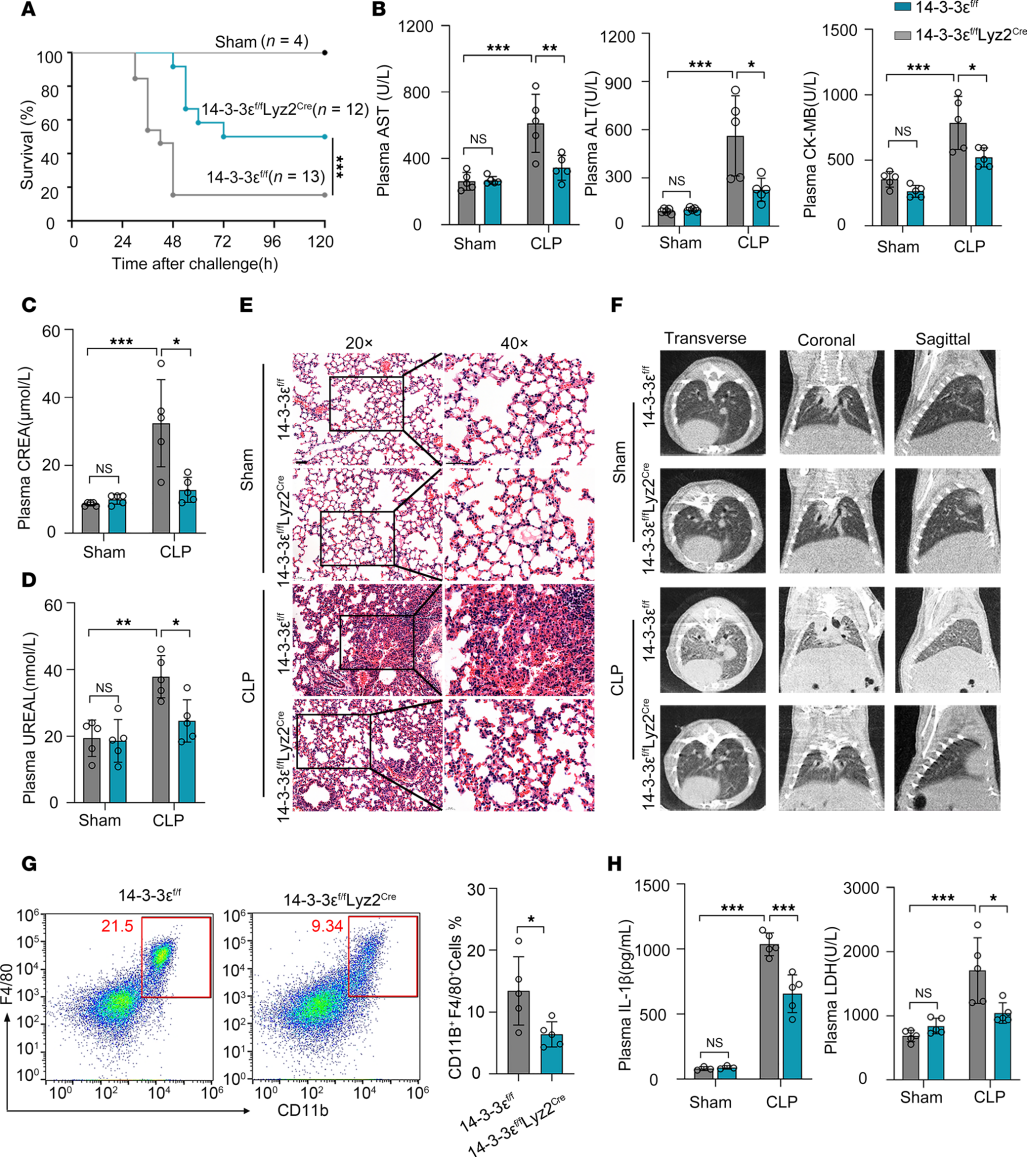
14-3-3ε deficiency in myeloid cells reduces organ damage and inflammatory injury in septic mice. (**A**) Survival rates of 14-3-3ε^fl/fl^ and 14-3-3ε^fl/fl^ Lyz2^Cre^ mice subjected to CLP surgery. Statistical analysis was performed using the log-rank (Mantel–Cox) test. (**B**–**D**) Blood biochemical indices AST, ALT, CK-MB, CREA, and UREAL in serum after 24 hours of sham or CLP surgery. (**E**) Representative H&E images of lungs sections after 24 hours of sham or CLP surgery. Scale bar: 50 μm. (**F**) Representative CT images of lungs after 24 hours of sham or CLP surgery. (**G**) Flow cytometric analysis of macrophages (CD11b^+^F4/80^+^) from the peritoneal cavity after 24 hours of sham or CLP surgery. (**H**) IL-1β levels in mouse serum were measured by ELISA, while blood LDH levels were quantified using an automated biochemistry analyzer. Data are presented as mean ± SD and were analyzed using 2-way ANOVA with Tukey’s multiple-comparison test (**B**–**D** and **H**) and 2-tailed *t* test (**G**); *P < 0.05; **P < 0.01; ***P < 0.001.

To investigate the effects of 14-3-3ε in sepsis, the 14-3-3ε flox (14-3-3ε^fl/fl^) mice were crossed with Lyz2-Cre transgenic mice to generate 14-3-3ε^fl/fl^ Lyz2^Cre^ mice (Supplemental Figure 7A). The Lyz2-Cre system typically was expressed in myeloid lineage cells, leading to deletion of target gene across myeloid cells (36). To assess KO efficiency, we performed flow cytometric analysis of 14-3-3ε expression in peritoneal macrophages, neutrophils, and dendritic cells (DCs) from 14-3-3ε^fl/fl^ and 14-3-3ε^fl/fl^ Lyz2^Cre^ mice. The results confirm complete 14-3-3ε ablation in macrophages and neutrophils, but intact expression in DCs of 14-3-3ε^fl/fl^ Lyz2^Cre^ mice (Supplemental Figure 7B). However, macrophage-conditional KO of 14-3-3ε did not affect frequencies of neutrophils and DCs in the peritoneal cavity of CLP mice (Supplemental Figure 7, C and D). Next, we performed CLP surgery in 14-3-3ε^fl/fl^ and 14-3-3ε^fl/fl^ Lyz2^Cre^ mice and observed the survival rates within 120 hours of surgery. We found that macrophage-conditional KO of 14-3-3ε considerably increased the survival of mice (Figure 7A). 14-3-3ε KO also decreased the levels of liver and renal function indicators, including aspartate aminotransferase (AST), alanine aminotransferase (ALT), creatine kinase isoenzyme MB (CK-MB), creatinine (CREA), ureal (UREAL) and uric acid (UA; Figure 7, B–D, and Supplemental Figure 8A), which suggests that macrophage-conditional KO of 14-3-3ε attenuated the liver and renal damage in septic mice. The pulmonary injury in 14-3-3ε^fl/fl^ and 14-3-3ε^fl/fl^ Lyz2^Cre^ mice upon CLP was detected using H&E staining and computed tomography (CT). H&E staining showed less infiltration of inflammatory cells and less injury in 14-3-3ε^fl/fl^ Lyz2^Cre^ mice than that in 14-3-3ε^fl/fl^ mice (Figure 7E). The ground glass opacities were also reduced in the lungs of 14-3-3ε^fl/fl^ Lyz2^Cre^ mice as observed in the CT scan (Figure 7F). Because CLP is a model of peritonitis, we collected peritoneal lavage fluid and measured the percentages of macrophages. The results of flow cytometric analysis showed that CD11b^+^F4/80^+^ macrophages tended to diminish (by approximately 15%–8%) in 14-3-3ε^fl/fl^ Lyz2^Cre^ mice (Figure 7G and Supplemental Figure 8B). Macrophages in the mouse peritoneal cavity are divided into large peritoneal macrophages (LPMs) and small peritoneal macrophages (SPMs) (37). We also found that 14-3-3ε KO in macrophages mainly downregulated the percentage of F4/80^–^MHC-II^hi^ SPMs but had no effect on LPMs (Supplemental Figure 8, C–E). Furthermore, KO of 14-3-3ε in macrophages reduced serum levels of IL-1β and LDH in CLP mice (Figure 7H), but had no significant effects on IL-1α or IL-6 levels (Supplemental Figure 8F). However, KO of 14-3-3ε in macrophages did not affect their phagocytosis and intracellular killing of P.A. (Supplemental Figure 8G). Taken together, these results indicate that conditional KO of 14-3-3ε in macrophages inhibited inflammasome activation and protected against CLP-induced organ damage in septic mice.

### Inhibition of 14-3-3 protects mice from polymicrobial sepsis.

**Figure 8:**
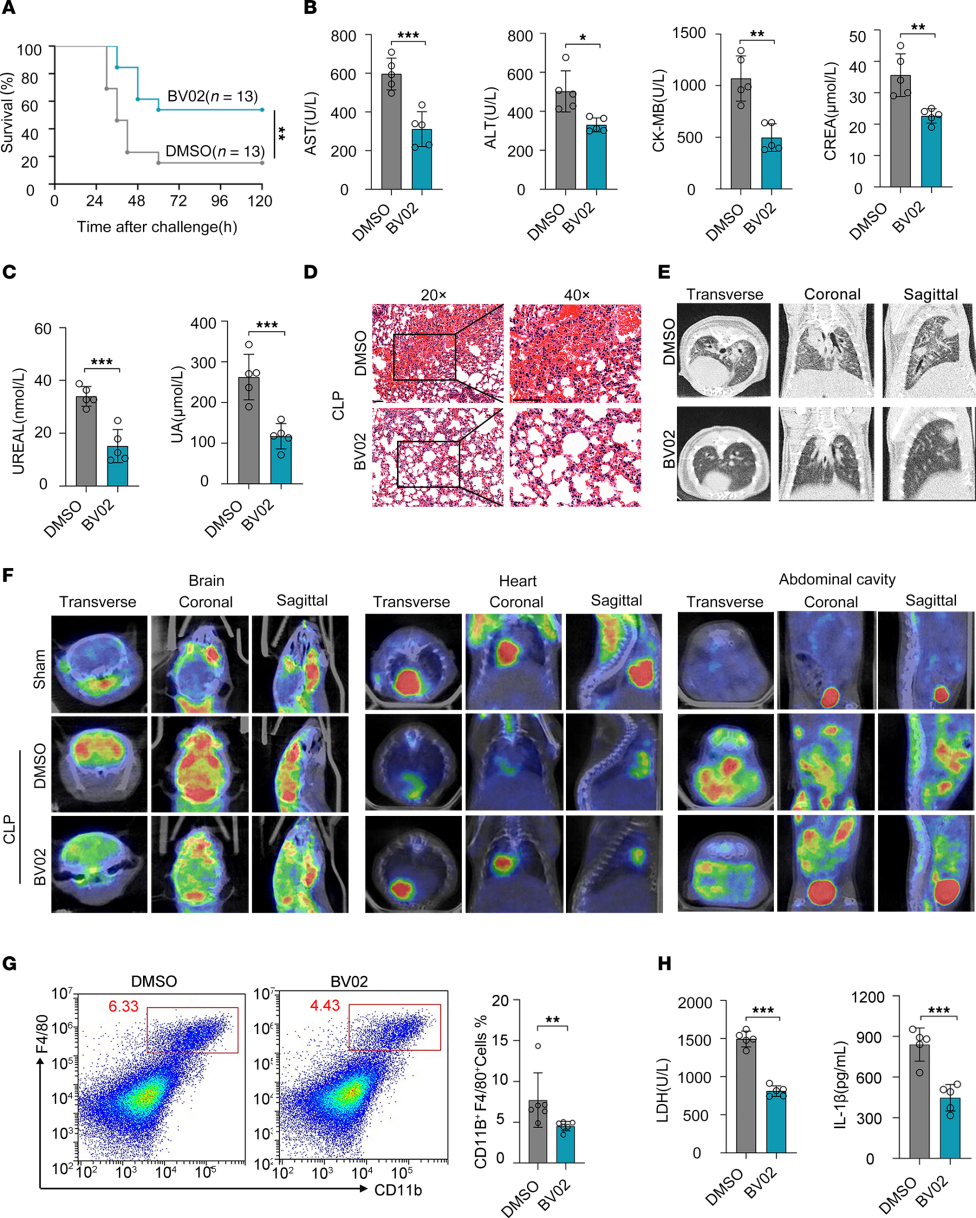
14-3-3 inhibitor protects mice from sepsis by alleviating organ injury and inhibiting inflammatory injury. (**A**) Mice were administered BV02 or DMSO (*n* = 13 per group) by i.p. injection at 12 hours after CLP, and the survival rates were observed. Statistical analysis was performed using the log-rank (Mantel-Cox) test. (**B**) Levels of AST, ALT, CK-MB, and CREA. (**C**) UREAL and UA in serum of septic mice 12 hours after treatment with DMSO or BV02 (*n* = 5 per group). (**D**) Representative H&E images of lung sections from DMSO or BV02-treated septic mice. Scale bar: 50 μm. (**E**) Representative CT images of lungs from DMSO or BV02-treated septic mice. (**F**) Representative PET scans of BV02-treated septic mice following 18 F-FDG treatment. (**G**) Flow cytometric analysis of macrophages (CD11b^+^F4/80^+^) from the peritoneal cavity. (**H**) IL-1β levels in mouse serum were measured by ELISA, while blood LDH levels were quantified using an automated biochemistry analyzer. Data are presented as mean ± SD and were analyzed using 2-tailed *t* test (**B**, **C**, **G**, and **H**); *P < 0.05; **P < 0.01; ***P < 0.001.

BV02 is a small-molecule inhibitor of 14-3-3 interactions that inhibits the binding of 14-3-3 to client proteins (38). We then induced sepsis using CLP and blocked 14-3-3 in vivo using BV02. The IC_50_ of BV02 for inflammasome inhibition was calculated to be 5.1 μM based on LDH levels in supernatants from LPS- and Nig-treated BMDMs (Supplemental Figure 9A).BV02-treated mice showed remarkably improved survival compared with DMSO-treated mice (Figure 8A). It also downregulated serum AST, ALT, CK-MB, CREA, UREAL, and UA levels (Figure 8, B and C), suggesting that BV02 attenuated the liver and renal damage in septic mice. For further analysis of the lung injury, H&E staining was performed, and the results show that BV02 treatment reduced the infiltration of inflammatory cells into the lungs (Figure 8D). CT scan also showed reduced ground-glass opacities in the lungs of BV02-treated mice (Figure 8E). Next, we assessed glucose metabolism using 18 F-FDG PET in view of the fact that elevated glucose metabolism in immune cells is a hallmark of sepsis (39, 40). The imaging results show that CLP promoted 18 F-FDG uptake in mouse brain, heart, and abdominal cavity, and BV02 reduced the promoted uptake in the whole body (Figure 8F). Flow cytometric analysis showed that CD11b^+^F4/80^+^ macrophages tended to diminish in BV02-treated mice (Figure 8G). Moreover, BV02 downregulated the percentage of F4/80^–^MHC-II^hi^ SPMs but had no obvious effect on LPMs (Supplemental Figure 9, B and C). In addition, BV02 treatment did not affect the proportion of CD11b^+^Ly6G^+^ neutrophils (Supplemental Figure 9D). BV02 treatment also reduced serum levels of IL-1β and LDH in CLP-modeled mice (Figure 8H) but had no significant effects on IL-1α or IL-6 levels (Supplemental Figure 9E). In addition, BV02 treatment did not affect the phagocytosis and intracellular killing of P.A. by macrophages (Supplemental Figure 9F). BV02 treatment inhibited inflammasome activation and decreased organ damage and systemic inflammatory responses in mice with sepsis.

## Discussion

Given that inappropriate NLRP3 inflammasome activation in macrophages is closely related to sepsis, inhibition of its activation has emerged as a promising therapeutic strategy for treating sepsis. In this study, we unraveled the role of 14-3-3ε in the activation of NLRP3 inflammasome, which explains one of the mechanisms controlling excessive inflammatory responses in sepsis and provides a potential therapeutic target for sepsis.

Extensive studies have established that 14-3-3ε serves as a key regulator of cell proliferation, differentiation, migration, autophagy and apoptosis through its interactions with various ligands (41). 14-3-3ε is recognized to suppress apoptosis by interacting with the BAD, thereby inhibiting both mitochondrial translocation of BAD and formation of BAD-related protein complexes in noninflammatory diseases such as ischemic cerebral infarction and colorectal cancer (42, 43). 14-3-3ε is also proposed to promote autophagy initiation through its crotonylation-mediated release of PPM1B, which subsequently activates ULK1, the core regulatory protein of autophagy, via dephosphorylation (23, 44). In our study, genetic ablation of 14-3-3ε did not alter LPS- and Nig-induced apoptosis, necrosis, or autophagy in BMDMs, suggesting its specific role in inflammasome activation rather than general cell death regulation under these conditions. Furthermore, 14-3-3ε participates in regulating multiple signaling pathways, including TGF-β–mediated signaling, Wnt/β-catenin pathway, PI3K pathway, NF-κB signaling, and Hedgehog (Hh) signaling transduction (41). Our results show that 14-3-3ε KO did not affect the LPS-induced NF-κB/MAPKs signaling pathway activation in BMDMs. It is possible that 14-3-3ε has different roles in different cell types or in different activation status, and future studies should further investigate how 14-3-3ε regulates sepsis pathogenesis through diverse signaling pathways and distinct cell death modalities.

Our data demonstrate that 14-3-3ε protein levels remain unchanged upon bacterial infection or LPS and Nig stimulation, while it promotes NLRP3 inflammasome activation. We propose that the interaction between 14-3-3ε and NLRP3 remains unsaturated under resting conditions. During sepsis, increased phosphorylation of NLRP3 enhances its binding to 14-3-3ε, which then facilitates NLRP3 translocation to MAMs through an as-yet-undefined mechanism, ultimately leading to NLRP3 inflammasome activation. Previous studies have demonstrated that the phosphorylation of serine and tyrosine is critically important in the protein-protein interactions of 14-3-3. For instance, 14-3-3ε interacts with p53, in a manner dependent on phosphorylation at S366, S378, and T387 of p53 (27). Moreover, 14-3-3 binds to the phosphorylated S179 of MdWRKY18, enhancing its stability and transcriptional activation activity (45). We found that NLRP3 S194 phosphorylation enhances its interaction with 14-3-3ε. Notably, sepsis upregulates both NLRP3 protein expression and its S194 phosphorylation (30), establishing the molecular basis for their increased association under septic conditions. Moreover, Sepsis may trigger the translocation of 14-3-3ε to MAMs, concurrently inducing the cotransport of NLRP3 with 14-3-3ε. In our study, both NLRP3 and 14-3-3ε translocate to MAMs following LPS and Nig stimulation, while 14-3-3ε KO impairs NLRP3 recruitment to MAMs. 14-3-3ε is widely reported to regulate protein distribution (32, 46). 14-3-3ε resides in the cytosol in the resting state, but it controls the translocation of certain proteins when stimulated (47, 48). Previous studies have shown that 14-3-3ε regulates the redistribution of RIG-I from the cytosol to organellar membrane structures (49, 50). 14-3-3ε and 14-3-3β could also bind to SAC1, which controls SAC1 export from the ER (51). Dependent on our experimental findings, we propose that 14-3-3ε may function as a transport adaptor to facilitate NLRP3’s subcellular localization at MAMs, thereby spatiotemporally regulating inflammasome activation.

BV02 is a phthalimide derivative that functions as a 14-3-3 protein-protein interaction inhibitor. It has been widely utilized in chemical biology studies to elucidate the role of 14-3-3 proteins in pathological contexts (52). It is an inhibitor targeting the scaffolding protein docking site of 14-3-3, effectively suppressing its scaffolding function (38, 53). BV02 binds to the amphipathic groove of 14-3-3 proteins, thereby competitively inhibiting the binding of other proteins through conserved phosphopeptide motifs (38). BV02 has been reported to disrupt the interaction between 14-3-3 proteins and their phosphorylated target proteins, such as ABL1 (54), PfCDPK1, and PfPKAr (55). In this study, our molecular docking analysis revealed that NLRP3 likely engages with the amphipathic groove of 14-3-3ε. We therefore hypothesize that BV02 may competitively bind to the amphipathic groove of 14-3-3ε, thereby inhibiting its interaction with NLRP3. Naturally, this hypothesis requires further validation through structural characterization in future studies. BV02 was initially identified as an inhibitor of 14-3-3σ, and has been widely utilized as a pan-14-3-3 family inhibitor (52, 56). 14-3-3 family contains 7 members, designated σ, β, ε, γ, η, τ, and ζ, which is highly conserved from yeast to mammals and has highly similar amino acid sequences and spatial structures (20). In this study, while the use of BV02 as a 14-3-3ε inhibitor inevitably carries potential off-target effects, our data demonstrate its substantial suppression of both systemic inflammation and key inflammasome activation markers (LDH release and IL-1β upregulation) in septic mice. These findings do not exclude potential regulatory roles of other 14-3-3 isoforms in inflammasome activation. However, the overall similarity between 14-3-3 inhibition and 14-3-3ε KO in sepsis suggests that either 14-3-3ε plays a dominant regulatory role compared with other isoforms in sepsis, or multiple 14-3-3 members function redundantly with 14-3-3ε in sepsis.

In summary, this study provides evidence that 14-3-3ε is an important positive regulator of NLRP3 inflammasome activation in sepsis and reveals that 14-3-3ε interacts directly with NLRP3 to promote K63 deubiquitination of NLRP3 and its translocation to MAMs. Our study sheds light on the regulatory roles of 14-3-3ε in sepsis and uncovers the mechanism of regulation of NLRP3 inflammasome activation by 14-3-3ε, which may provide potential avenues for the development of therapeutic strategies for sepsis.

## Methods

### Sex as a biological variable.

For clinical samples, both sexes were involved. For animal models, only male mice were examined to reduce female sexual cycle–related variation. The findings were expected to be relevant to both sexes.

### Patients.

Patients with sepsis and healthy control were recruited from the Fifth Affiliated Hospital of Sun Yat-sen University (Zhuhai, China). Patients were diagnosed as sepsis according to the guideline from the Third International Consensus Definitions for Sepsis and Septic Shock (Sepsis-3). Detailed clinical characteristics and laboratory information are shown in Supplemental Table 2. *Animals*. Lyz2^Cre^ mice (no. T003822) and the 14-3-3ε^fl/fl^ mice (no. T013265) were purchased from GemPharmatech (Nanjing, China). 14-3-3ε^fl/fl^ Lyz2^Cre^ mice were generated by crossing homozygous 14-3-3ε^fl/fl^ mice with homozygous Lyz2^Cre^ transgenic mice. And the Cre recombinase eliminated the floxed exon 2 of 14-3-3ε. Tail-derived genomic DNA was subjected to PCR genotyping. All the mice were housed in a specificpathogen–free environment under standard conditions.

### Statistics.

Statistical analyses were performed using GraphPad Prism 8.0 (GraphPad Software, San Diego, USA). Statistical significance was determined using 2-tailed *t* test, one-way ANOVA, or 2-way ANOVA. Data are expressed as means ± SD. A *p*-value less than 0.05 was considered to indicate a significant difference.

### Study approval.

All samples were collected according to the guidelines from the Ethics Board of Fifth Affiliated Hospital of Sun Yat-sen University (ethics no. L088-1) and informed written consents were obtained from all participants prior to the commencement of the study. All the animal experiments were approved by the Ethics Committee of the Fifth Affiliated Hospital of Sun Yat-sen University (ethics no. 00171).

### Data availability.

Values for all data points in graphs are reported in the Supporting Data Values file.

## Author contributions

XL and SM contributed equally to this work. XL designed and performed molecular biology experiments and figure preparation. SM conducted animal studies and statistical analysis. Both authors jointly wrote the manuscript and approved the final version. XL, SM, CC, NT, and YX conceived, designed, and supervised the study. XL and CC performed most of the experiments. XL and YW conducted part of data analysis. JS performed part of cell studies. XL, XH, and YW provided funding for this study. All authors read and approved the final version of the manuscript.

## Funding support

- The National Natural Science Foundation of China (82402523, 82072062, 82571550)
- The GuangDong Basic and Applied Basic Research Foundation (2023A1515110966)
- China Postdoctoral Science Foundation (2024M753749)
- The Natural Science Foundation of Guangdong Province (2023A1515030065)
- The open research funds from the Sixth Affiliated Hospital of Guangzhou Medical University, Qingyuan People’s Hospital (202301-102)

## Supplementary Material

Supplemental data

Unedited blot and gel images

Supporting data values

## Version 1 01/09/2026

Electronic publication
