# Supplementary material for: 14-3-3ε–dependent deubiquitination and translocation of NLRP3 activates the inflammasome during sepsis
Source: JCI Insight. 2026 Jan 9;11(1):e192970. doi: 10.1172/jci.insight.192970 (PMC12890513; doi:10.1172/jci.insight.192970)
Supplement: Supplemental data [file jciinsight-11-192970-s210.pdf]

Supplementary Materials and Methods

Cell culture

BMDMs were isolated from the femur and tibia of 4–6-week-old mice and differentiated into macrophages in DMEM (ThermoFisher, C11995500BT) supplemented with 10% FBS (GIBCO, 10099-141), 1% penicillin/streptomycin (Vivacell, C3420-0100), and 30% L929 (from ATCC) cell culture supernatant for 7 days. iBMDMs (from National Institute of Biological Sciences, Beijing, China) and HEK-293T cells (from ATCC) were cultured in DMEM supplemented with 10% FBS and 1% penicillin/streptomycin. The cells were cultured in an incubator (Thermo Fisher, US) at 37 ℃ and under a 5% CO_2 environment. We acknowledge Dr. Feng Shao (National Institute of Biological Sciences, Beijing, China) for the generous gifts of iBMDMs.

Bacterial culture

P. aeruginosa (ATCC19660) and *S. aureus* (ATCC43300) were cultured in Luria–Bertani medium overnight, and the bacterial cells were collected by centrifugation at 5000 ×*g* for 10 min. The bacterial count was estimated by measuring the optical density (OD) at 600 nm using a NanoDrop2000 spectrophotometer (Thermo Fisher, US; S.A.: OD_{600}1.0 = 1 × 10^9 CFU/mL; P.A.: OD_{600}1.0 = 8 × 10^8 CFU/mL). The LB medium was prepared as follows: 10 g tryptone (Solarbio, T8490), 5 g yeast extract (Solarbio, Y8030), and 10 g NaCl (Sigma-Aldrich, V90BV020058, 150 mM) were dissolved in 1 L ultrapure water, followed by autoclave sterilization before use.

BMDMs Differentiation

Bone marrow-derived macrophages (BMDMs) were generated via standard 7-day differentiation in complete DMEM supplemented with 30% L929 supernatant (as a

source of M-CSF) (1). Briefly, bone marrow was flushed from tibiae and femora of 4-6-week-old 14-3-3$\varepsilon^{f/f}$ or 14-3-3$\varepsilon^{f/f}$Lyz2^{Cre} mice using a 1-mL syringe with 25G needle. Erythrocytes were lysed with RBC lysis solution (Solarbio, China) for 3 min at room temperature. Nucleated cells were cultured in complete DMEM (10% FBS, 1% penicillin/streptomycin) supplemented with 30% L929 cell-conditioned medium. Medium was replaced on day 3 and 5 of differentiation. Mature BMDMs were harvested on day 7 of differentiation and subsequently used for functional assays.

siRNA Transfection in BMDMs

BMDMs were generated by culturing bone marrow cells in 6-well plates containing L929 cell-conditioned medium, allowing differentiation until reaching approximately 80% confluence. Following differentiation, the medium was exchanged for 1 mL of fresh BMDM culture medium supplemented with 10% FBS and maintained without penicillin-streptomycin. Then, 15μL si-NC or si-14-3-3ε (20 μM) was mixed with 15 μL INVI DNA RNA transfection reagent (Invigentech, US) and incubated at room temperature for 15 min. The resulting complexes were then added to BMDMs cultures and maintained for 24 hours. Next, medium was replaced with fresh medium, and incubation continued for 24 additional hours. Then the cells were collected for further experiments. The siRNA target 14-3-3ε was listed in Supplemental Table 3.

Plasmid construction and transient transfection

The cDNA sequences encoding mouse NLRP3 with a C-terminal Flag tag, mouse NLRP3 with a C-terminal HA tag, mouse NLRP3 with a C-terminal Myc tag, mouse 14-3-3ε with a C-terminal Flag tag were amplified by reverse transcription PCR. These fragments were then subcloned into the pcDNA3.1(+) vector following the manufacturer's protocol (GenScript). NLRP3 lacking the PYD (ΔPYD), NACHT

(ΔNACHT), or LRR (ΔLRR) domain were generated by deleting the PYD domain(1-91Aa), the NACHT domain(136-532Aa), or the LRR domain(739-1033Aa). Segmental and deletion clones were subcloned from the full-length NLRP3 plasmid. The mutations of NLRP3 at S194 and S291 were synthesized and constructed by JinsiruiBio Company (Nanjing, China). Cells were transiently transfected with plasmids or blank vector using Lipofectamine 2000 (Invitrogen, Thermo Fisher Scientific) as a previously described (2).

Co-IP and western blot analyses

For co-IP, cells were lysed in RIPA lysis buffer (pH 7.4) containing Tris (Fude, FD2010, 50 mM), NaCl (Sigma-Aldrich, V900058, 150 mM), NP-40 (Biosharp, BS205, 1%), and a protease inhibitor cocktail (Selleck, B14001, 1%) on ice and the lysate was centrifuged at 10,000 × *g* for 10 min at 4 ℃. After centrifugation, the supernatant was incubated overnight at 4 ℃ with protein A/G beads (Merck Millipore, LSKMAGAG02) and the corresponding antibodies. The beads were then washed five times with RIPA buffer and boiled with 5×SDS-PAGE loading buffer (Fude, FD002) at 100 ℃ for 10 min to release the proteins. To detect the exdogenous association, HEK-293T cells were transfected with the indicated plasmids and the cell lysate was incubated overnight at 4 ℃ with EZview™ Red Anti-HA Affinity Gel (Sigma-Aldrich, E6779), anti-Flag M2 affinity beads (Sigma-Aldrich, M8823), or EZview™ Red Anti-c-myc Affinity Gel (Sigma-Aldrich, E6654). Western blot analysis was performed according to methods described in previous studies (3) using the indicated antibodies. Primary and secondary antibodies were employed with the following specifications: anti-Caspase-1 (p20, AdipoGen, AG-20B-0042, 1μg/mL); anti-NLRP3 antibody (AdipoGen, AG-20B-0014, 1μg/mL); anti-NLRP3 antibody (Abcam, ab270449, 1μg/mL); anti-IL-1β (R&D, BAF401, 1μg/mL); anti-14-3-3ε antibody (Novus, NBP1-89827, 1μg/mL); anti-beta-

actin (Cell Signaling Technology, 4970, 1μg/mL); anti-ASC antibody (Cell Signaling Technology, 67824, 1μg/mL); anti-HA antibody (Cell Signaling Technology, 3724, 1μg/mL); anti-FLAG M2 antibody (Cell Signaling Technology, 14793, 1μg/mL); anti-Myc antibody (Cell Signaling Technology, 2276, 1μg/mL); anti-GAPDH antibody (Abcam, ab181602, 1μg/mL); anti-Ubiquitin antibody (Cell Signaling Technology, 43124, 1μg/mL); anti-β-tublin (Cell Signaling Technology, 2146S, 1μg/mL); anti-Cox-1 antibody (Cell Signaling Technology, 9896, 1μg/mL); anti-VDAC antibody (Cell Signaling Technology, 4661, 1μg/mL); anti-mitofusin-2 antibody (Cell Signaling Technology, 9482, 1μg/mL); anti-Calnexin antibody (Novus, NB100-1965, 1μg/mL); anti-FACL4 antibody (Abcam, ab205199, 1μg/mL); Goat Anti-Mouse IgG-HRP Conjug (BIO-RAD, 1706516, 1:2000); Goat Anti-Rabbit IgG-HRP Conjug (BIO-RAD, 1706515, 1:2000).

Protein structure prediction and molecular docking

The protein sequences (NLRP3: Q8R4B8; 14-3-3ε: P62259) were obtained from the UniProt database. The structure prediction of the protein complex was accomplished using AlphaFold3. The binding free energy of the best conformation was calculated using the MOE software version 2019.0102. Molecular visualization and residue interaction analyses were performed using PyMOL version 2.5.5.

ELISA

ELISA kit for detecting the levels of 14-3-3ε in human plasma were purchased from Huayun Biotechnology. ELISA kit for detecting the levels of IL-1β in human plasma were purchased from Dakewei Biotechnology. ELISA kit for detecting the levels of mouse IL-1β, IL-1αand LDH were purchased from MEIMIAN. ELISA kit for detecting the levels of IL-6 in mouse serum were purchased from Thermo Fisher.

qPCR assay

Cells were lysed with TRIzol (ThermoFisher, 15596018) and RNA was isolated and amplified as previously reported (3, 4). qPCR was performed using GoTaq qPCR Master Mix (Promega, A6002) on a Bio-Rad CFX96 real-time PCR system (Bio-Rad, US). The list of primers is included in Supplemental Table 2.

Immunofluorescence staining

For staining HEK-293T cells and BMDMs, the cells were seeded in disposable confocal dishes and processed accordingly. For ASC speck staining, peritoneal macrophages were seeded in the wells of 12-well plates with cell climbing slices and stimulated with LPS (Sigma-Aldrich, L8274, 400 ng/mL) for 5 hours. Thereafter, the cells were stimulated with Nig (Macklin, N849347, 20 μM) for 30 min or left unstimulated. The cells were fixed with paraformaldehyde and permeabilized using 0.3% Triton X-100 (Biosharp, BS084) in 5% bovine serum albumin (BSA, Biotechology, BW0332) solution, and subsequently incubated with the primary antibodies overnight at 4 ℃. The cells were then washed three times with PBS and incubated with the corresponding secondary antibodies coupled with fluorophores at 37°C for 1 h. The cells were then stained with DAPI (Solarbio, C0065) for 5 min and viewed using a confocal microscope (Zeiss LSM 880, Germany). Primary and secondary antibodies were employed with the following specifications: anti-NLRP3 antibody (Abcam, ab270449, 5μg/mL); anti-ASC antibody (Cell Signaling Technology, 67824, 5μg/mL); anti-FLAG Tag antibody (Novus, NBP1-06712SS, 1μg/mL); anti-HA antibody (Cell Signaling Technology, 3724, 5μg/mL); anti-FACL4 antibody (Abcam, ab205199, 5μg/mL); anti-14-3-3ε antibody (Thermo Fisher Scientific, MA5-49207, 5μg/mL); Goat Anti-Rat IgG H&L (Alexa Fluor® 594, Abcam, ab150160,1:500); Goat Anti-Rabbit IgG H&L (Alexa Fluor® 594, Abcam, ab150080,1:500); Goat Anti-Rabbit IgG H&L (Alexa Fluor® 488,

Abcam, ab150077,1:500); Goat anti-Mouse IgG (H+L) Cross-Adsorbed Secondary Antibody Alexa Fluor 594 (Thermo Fisher Scientific, A-11020, 1:500).

Inflammasome activation

Induced BMDMs or iBMDMs were seeded at a density of 5×10^5 cells/well in 12-well plates. Once adherent, the culture medium was replaced with Opti-MEM® I Reduced Serum Medium (Thermo Fisher, US). Thereafter, the cells were treated with 200 ng/mL LPS for 3 h and then were stimulated separately with Nig (10 μM) for 30 min or ATP (TargetMol, T20089, 50 μM) for 50 min, or with poly (dAdT, InvivoGen, tlrl-patn, 2 μg/mL) transfection for 6 h, or flagelin (Macklin, F879697, 1 μM) transfection for 6 h. The supernatant and whole-cell lysate were collected. The supernatant was mixed with methanol (Aladdin, M116118) and chloroform (Guangzhou, GSSA02-TD) at a ratio of 4:4:1 to separate the proteins. The mixture was then centrifuged at 10,000 $\times g$ for 15 min at 4 ℃. The supernatant was discarded, and the pellet was washed three times with methanol. After drying at room temperature for 15 min, the protein pellet was dissolved in 50 μL RIPA lysis buffer for western blot.

ASC oligomer crosslinking

BMDMs were stimulated with LPS (200 ng/mL) for 3 h and stimulated with 20 μM Nig or 50 μM ATP for 1 h. The cells were lysed with a Triton-X buffer containing 50 mM Tris-HCl pH 7.5, 150 mM NaCl, 0.5% TritonX-100, and EDTA-free protease inhibitor cocktail, and the lysate was centrifuged at 6000 $\times g$ for 15 min at 4 ℃. The soluble fraction was mixed with 5× loading buffer (Fude, FD002) and denatured by heating at 95 ℃ for 10 min and the insoluble fraction was crosslinked with 2 mM disuccinimidyl suberate (DSS, Thermo Scientific, 21655) for 30 min at 37 ℃. After centrifugation at 6000 $\times g$ for 15 min at 4 ℃, the crosslinked pellet was obtained and

resuspend in Triton-X buffer. The mixture was then mixed with 5× loading buffer and denatured by heating at 100 ℃ for 10 min.

SDD-AGE

BMDMs transfected with si-NC or si-14-3-3ε were lysed with a lysis buffer containing 0.5% Triton X-100, 50 mM Tris–HCl, 150 mM NaCl, 10% glycerol (Macklin, G6201), and 1% protease inhibitor. The cell suspension was homogenized by passing it through 21-guage needles 20 times and resuspended in 5× sample buffer (0.5× Tris–Borate–EDTA, TBE, Beyotime, ST718, 10% glycerol, 2% SDS, Acmec, 151-21-3, and 0.0025% bromophenol blue, Macklin, B802654). The lysate was loaded onto a 1.5% agarose gel. After electrophoresis in the running buffer (1× TBE comprising 89 mM Tris, pH 8.3, 89 mM boric acid, and 2 mM EDTA, Macklin, E809068 and 0.1% SDS) at a stable voltage of 80 V for 1 h, the proteins were transferred onto a PVDF membrane (Merck Millipore, IPVH00010) and the membranes were blocked and incubated with anti-NLRP3 antibody (Abcam, ab270449, 5μg/mL); followed by incubation with HRP-conjugated secondary antibodies.

Membrane flotation

The membrane flotation experiments were performed according to the previous studies (5, 6). Briefly, the treated cells were digested and resuspended using 1mL lysis buffer containing 10mM Tris-HCl (EcoTop Bio, ES-8066); 10mM KCl (Aladdin, P656969), and 5mM $MgCl_2$ (Sinopharm, 10012818) and were lysed with a 25-gauge needle and syringe 20 times and were incubated on ice for 20 min. Cell suspension was centrifuged at 1,000 × g for 5 min at 4 °C. After centrifugation, the supernatant was mixed with 3mL hypotonic lysis buffer containing 50mM Tris-HCl, 25mM KCl, 5mM $MgCl_2$, and 72% sucrose (Macklin, S818049). Subsequently, 4 mL of hypotonic lysis buffer

containing 55% sucrose and 1.5 mL of hypotonic lysis buffer containing 10% sucrose were slowly added to obtain a delaminated mixture. Samples were centrifuged at 38000 rpm for 14 hours at 4 ℃. After centrifugation, gradients were fractionated from the top into 8 equal fractions (about 1.2mL/fraction). 0.8mL of hypotonic lysis buffer was added to every fraction and the mixture was concentrated by ultrafiltration tubes (Beyotime, FUF051) for western blot.

Organelle isolation

The organelle isolation experiments were performed according to the previous studies (7, 8). Briefly, the treated cells were digested and resuspended using a lysis buffer 1 containing 225mM D-mannitol (Beyotime, ST2362), 75mM sucrose, 0.1mM EDTA (Macklin, E809068), 30mM Tris-HCl; and were lysed with a 25-gauge needle and syringe 20 times. The homogenate was centrifuged at 600 × *g* for 5 min to remove unbroken cells. Then the supernatant was centrifuged at 7,000 × *g* for 10 min, and the cell supernatant (S1) and pellet (P1) were collected. The supernatant (S1) was incubated on ice for 1.5 hours and was centrifuged at 20,000 × *g* for 30 min, then the new supernatant was then transferred to new tubes and ultracentrifuged at 100,000 × *g* for 1 hour at 4 °C to collect ER (pellet) and cytosol (supernatant). The pellet (P1) were washed twice times with the lysis buffer 1 and resuspended in a lysing buffer 2 containing 250mM D-mannitol, 5mM HEPES (Sigma-Aldrich, 7365-45-9), 0.5mM EDTA. This mixture was added on top of percoll medium (Sigma-Aldrich, P4937) containing 250mM D-mannitol, 25mM HEPES, 1mM EDTA, 30% percoll, then lysing buffer 2 was added to the upper layer. After centrifugation at 95,000 × *g* for 30 min, the liquid was divided into three layers. The middle-layer and lower-layer liquid were collected and mixed with lysing buffer 2. The middle-layer mixture was centrifuged at 100,000 ×g for 1 hour, the precipitated were collected and extracted as MAMs fraction.

The lower-layer mixture was centrifuged at 6,300 ×g for 10 min, the resulting precipitate was the mitochondrial fraction.

CLP mouse model

The mice were anesthetized with pentobarbital via intraperitoneal injection. The mice were fixed, with abdomen facing upwards, and the abdomen was disinfected with 75% alcohol. A longitudinal midline incision was made using a scalpel to expose the cecum, which is located on the left side of the abdomen. The cecum was ligated midway between the distal pole and the cecum base, followed by one needle puncture. A few fecal pellets were squeezed out of the perforation sites, the cecum was returned to the abdomen, and the incision was closed with sutures and skin staples. For the sham surgery, cecal ligation, cecal perforation, and fecal extrusion were omitted. After 24 h, serum was collected for biochemical detection using an automatic biochemical analyzer, the lung sections were used for H&E staining, cells in peritoneal lavage were collected for flow cytometric analysis, and PET and CT were performed using a nanoScan PET/CT 82s scanner.

Flow cytometry

Flow cytometry was performed as previously described (4). Mouse peritoneal lavage fluid was centrifuged at 800 ×*g* for 5 min to obtain cell pellets. Human peripheral blood neutrophils and monocytes were isolated using a human peripheral blood neutrophil isolation kit (Solarbio, P9040). For surface molecule staining, cells were blocked with PBS containing 1% BSA, stained with the corresponding fluorescent antibody, washed, and fixed with a 1% paraformaldehyde solution (Biosharp, BL539A30525-89-4). For 14-3-3ε staining, the cells were fixed with a IC Fixation Buffer (Invitrogen, 00-8222-49) and permeabilized using a permeabilization buffer (Invitrogen, 00-8333-56) after

the surface molecules were stained. Subsequently, cells were stained with 14-3-3ε antibody (Invitrogen, MA5-49207, 1μg/mL) at 4 ℃ for 30 min. After washing, cells were stained with a PE-conjugated secondary antibody (Southern Biotech, 114409) for 30 min and fixed with a 1% paraformaldehyde solution. All flow cytometric assays were performed using a Cyto-FLEX Flow cytometer (BECKMAN COULTER Life Sciences, US). Antibodies were employed with the following specifications: APC anti-mouse F4/80 (BioLegend, 123115); FITC anti-mouse/human CD11b (BioLegend, 101206); PE anti-mouse I-A/I-E (BioLegend, 107608); PE/Cyanine7 anti-mouse Ly-6G (BioLegend, 127618), APC anti-Mouse CD11c(Proteintech, APC-65130). Pacific Blue anti-human CD14 (BioLegend, 325615); APC-Cy7 anti-human CD16 (BD, 560248); PE/Cyanine7 anti-human CD15 (BioLegend, 323029); PE anti-mouse IgG2b(BioLegend, 406708).

Plate count to evaluate phagocytosis and intracellular killing of P.A.

BMDMs were differentiated using L929 supernatant and then infected with P. A. at an MOI of 25. Cells in the BV02 treatment group were pretreated with 5 μg/mL BV02 for 4 hours prior to infection. After 1 hour of infection, gentamicin (300μg/mL) was added to the culture medium and incubated for 30 min to eliminate extracellular bacteria. Subsequently, cells were washed three times with PBS to remove extracellular P. A., followed by lysis with 0.1% Triton X-100. The number of phagocytosed viable bacteria was quantified using colony counting assays. The phagocytosis efficiencies were quantified as colony-forming units (CFU) per cell [CFU (1 h)]. To determine the intracellular bactericidal activity against P. A., extracellular bacteria were removed 1 hour by gentamicin treatment. After an additional 1-hour incubation, cells were lysed and plated for colony counting assays to quantify viable bacteria, with results expressed as CFU per cell [CFU (2h)]. The intracellular killing rate was determined by the

following formula: Intracellular bacterial killing = [CFU (1 h) − CFU (2 h)]/CFU (1 h) × 100%.

IC50 assay

IC50 assay was performed by detecting LDH release into culture supernatants. In brief, BMDMs were seeded into a 6-well plate at a density of 5×10^5 cells per well. Then, BV02 was diluted with fresh medium at a gradient concentration of 0, 0.5, 1.0, 1.5 and 2.0 μM, and added into the cells. Following 12 hours LPS and Nig stimulation, supernatants were centrifuged (4000 ×g, 5 min) and assayed for LDH activity using ELISA. The highest LDH concentration was set to 100% for each experiment, and other concentrations are normalized relative to this value. The IC50 value was calculated by GraphPad Prism 8.0 (GraphPad Software, San Diego, CA, USA).

Supplementary Figures

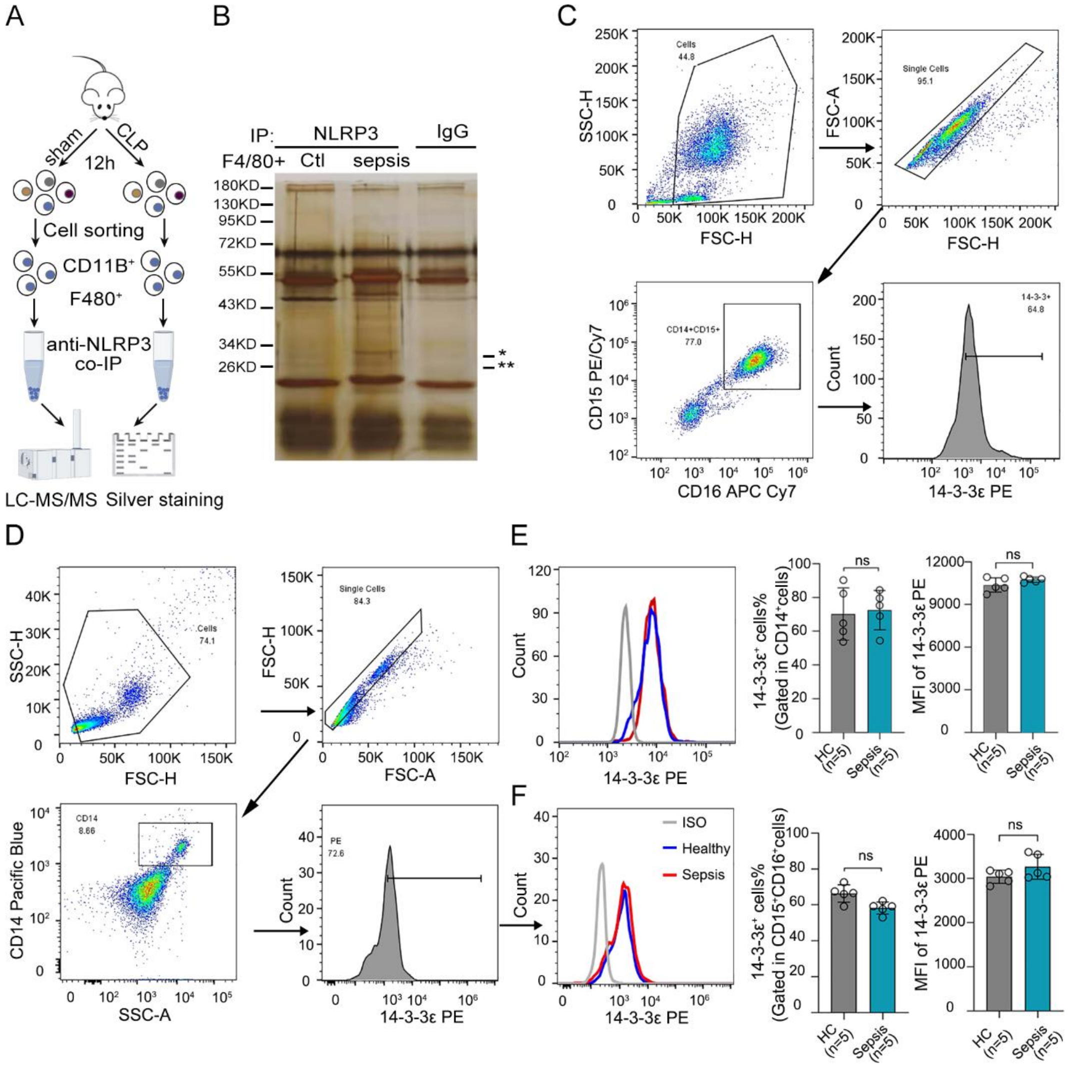

Supplemental Figure 1. Expression levels of 14-3-3ε in sepsis. (A) The flowcharts of LC–MS/MS analysis and silver staining. (B) The representative silver-stained gel image of silver staining, differential bands are marked by asterisk (*). (C, D) Gating strategy for 14-3-3ε expression in $CD15^{+}CD16^{+}$ neutrophils and $CD14^{+}$monocytes from peripheral blood of healthy controls and patients with sepsis. (E, F) The percentage of 14-3-3ε-positive cells, mean fluorescence intensity (MFI) of 14-3-3ε, and representative 14-3-3ε expression histograms in neutrophils and monocytes from peripheral blood of healthy controls (n=5) and patients with sepsis (n=5). Data are

presented as mean ±SD and were analyzed using unpaired Student's t-test (E, F). ns, nonsignificant.

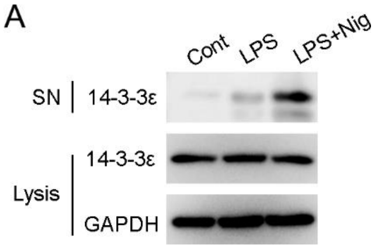

Supplemental Figure 2. The expression level of 14-3-3ε in cell culture supernatants. (A) Western blot analysis of 14-3-3ε expression levels in the supernatants of BMDMs treated with LPS alone or LPS combined with Nig.

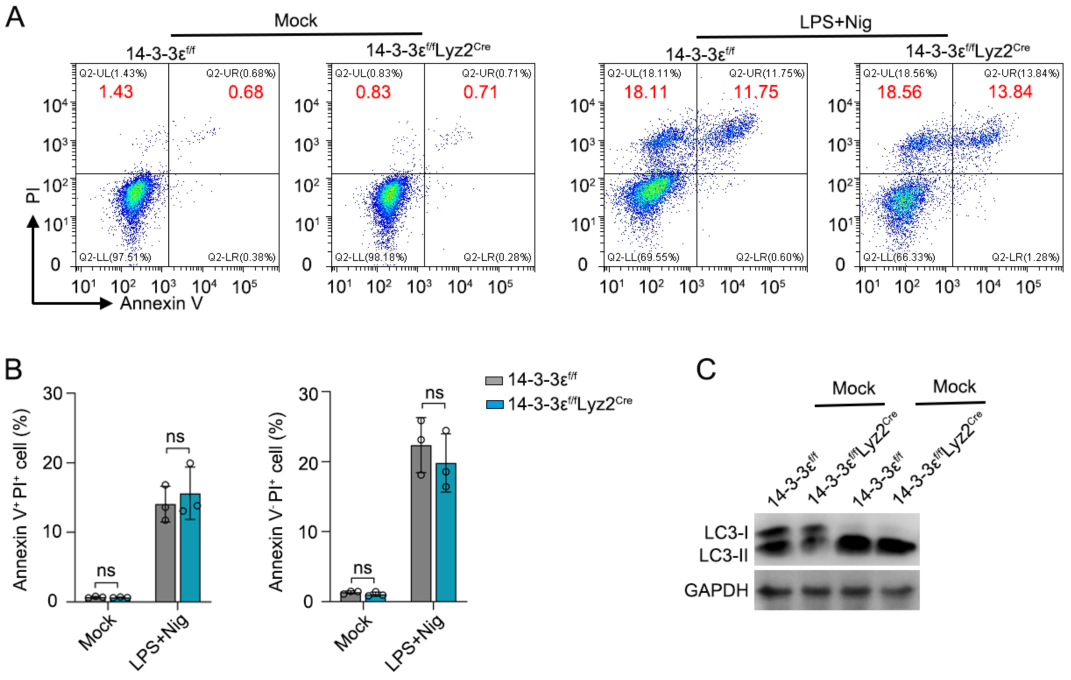

Supplemental Figure 3. 14-3-3ε did not affect macrophage apoptosis, necrosis, or autophagy under LPS and Nig treatment. (A) Representative flow cytometry plots of Annexin V/PI staining in LPS and Nig-treated14-3-3ε$^{f/f}$ and 14-3-3ε$^{f/f}$Lyz2^{Cre} BMDMs. (B) Quantitative analysis of cell apoptotic and necrotic cell populations. (C)

Representative Western blot images of LC3 in LPS and Nig-treated14-3-$3\varepsilon^{f/f}$ and 14-3-$3\varepsilon^{f/f}$Lyz2^{Cre} BMDMs. Data are presented as mean ±SD and were analyzed using two-way ANOVA with Tukey’s multiple-comparison test (A). ns, nonsignificant.

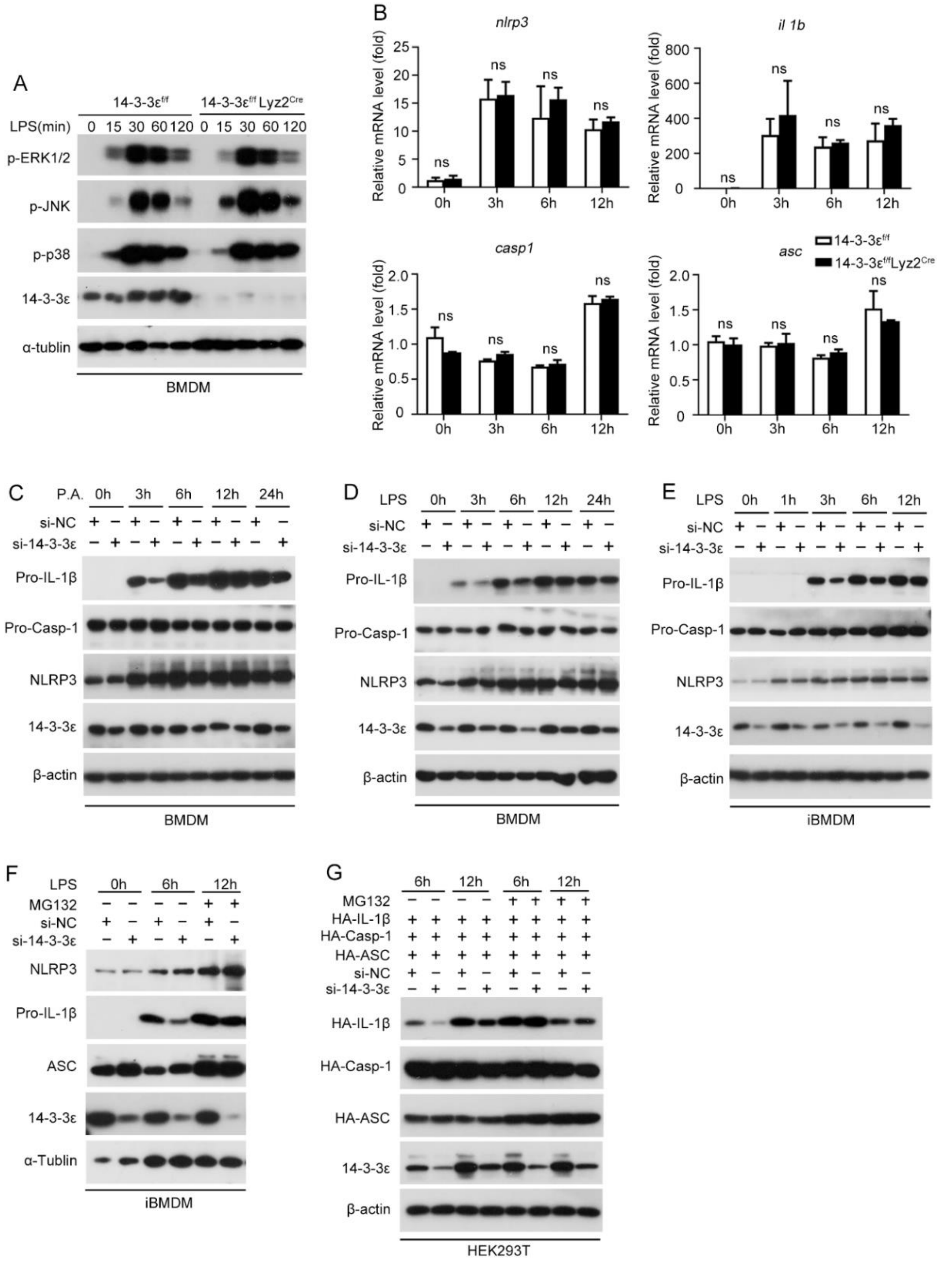

Supplemental Figure 4. 14-3-3ε does not affect NLRP3 protein levels but promotes IL-1β expression via inhibiting ubiquitin-mediated degradation. (A) Western blot analysis of phosphorylated signaling molecules of the MAPKs signaling pathways in BMDMs from 14-3-3ε$^{f/f}$ or 14-3-3ε$^{f/f}$Lyz2^{Cre} mice after stimulation with LPS for the indicated time periods. (B) The mRNA levels of *nlrp3*, *il1b*, *caspase1*, and *asc* in

BMDMs from 14-3-3ε$^{f/f}$ mice or 14-3-3ε$^{f/f}$Lyz2^{Cre} mice and treated with LPS for different time points. (C) Western blot analysis of 14-3-3ε, NLRP3, pro-IL-1β, and pro-caspase1 in si-NC- or si-14-3-3ε-transfected BMDMs with P.A. infection at the indicated time points. (D, E) Western blot analysis of 14-3-3ε, NLRP3, pro-IL-1β, and pro-casp-1 in si-NC- or si-14-3-3ε-transfected BMDMs and iBMDMs with LPS treatment at the indicated time points. (F) Western blot analysis of 14-3-3ε, NLRP3, pro-IL-1β, pro-casp-1 in si-NC- or si-14-3-3ε-transfected iBMDMs with LPS treatment at the indicated time points following MG132 treatment. (G) Western blot analysis of 14-3-3ε, NLRP3, pro-IL-1β, pro-casp-1 in si-NC- or si-14-3-3ε-transfected HEK-293T cells, which were transfected with HA-tagged IL-1β, caspase 1, and ASC and treated with MG132. Data are presented as mean±SD and were analyzed using two-way ANOVA with Tukey's multiple-comparison test (B). ns, nonsignificant.

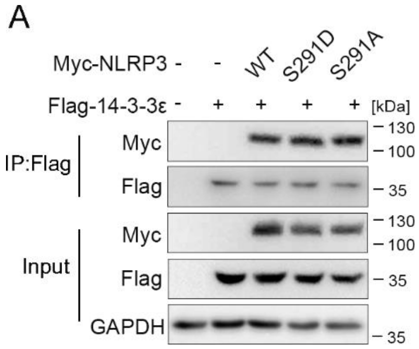

Supplemental Figure 5. 14-3-3ε-NLRP3 interaction does not depend on NLRP3 phosphorylation at S291 position. (A) Co-IP analysis of the interaction between 14-3-3ε and S291A/S291D NLRP3 proteins in HEK-293T cells by transfecting with the expression plasmids corresponding to the indicated proteins.

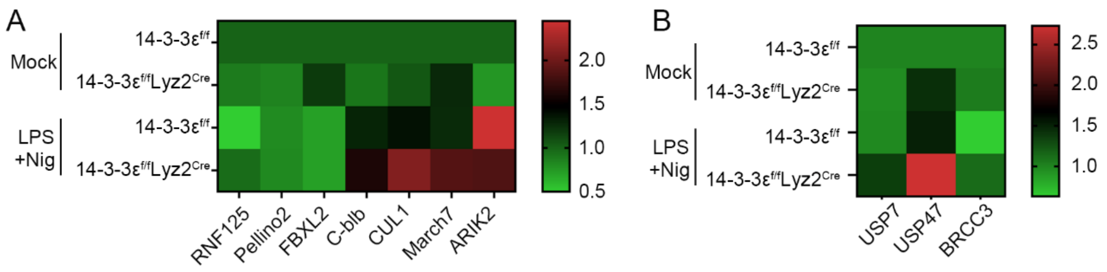

Supplemental Figure 6. RNA expression levels of ubiquitin and de-ubiquitin ligases. BMDMs from 14-3-3ε$^{f/f}$ mice or 14-3-3ε$^{f/f}$Lyz2^{Cre} mice were primed with LPS and stimulated with Nig, and the RNA expression levels of indicated (A) ubiquitin ligases and (B) de-ubiquitin ligases were detected by q-PCR.

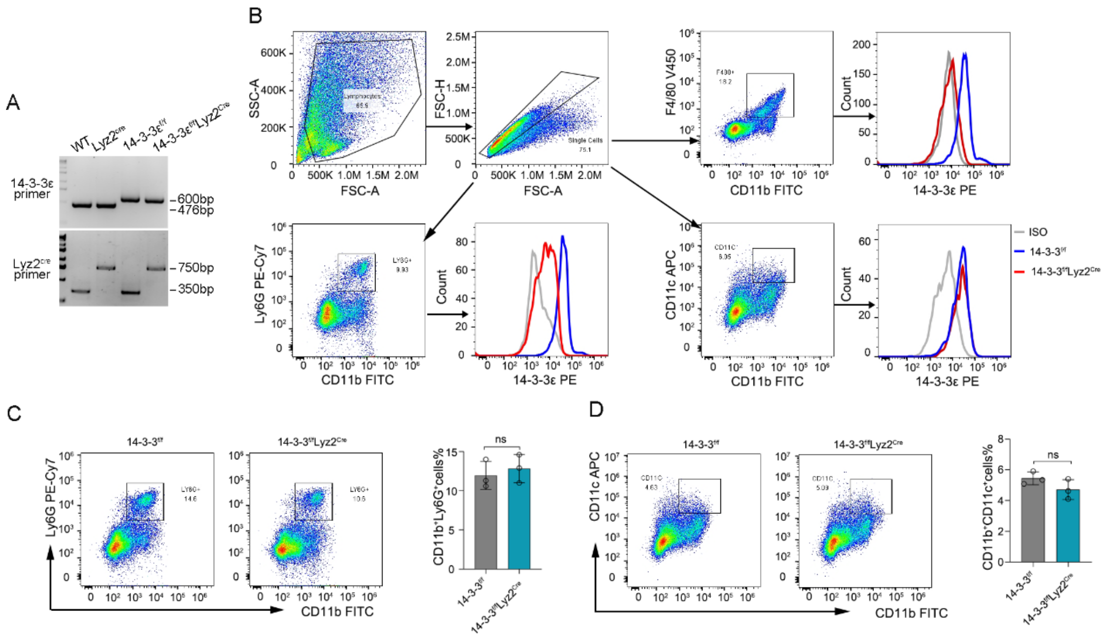

Supplemental Figure 7. 14-3-3ε$^{f/f}$Lyz2^{Cre} conditional knockout did not affect the recruitment of neutrophils and DCs into peritoneal cavities of septic mice. (A) Genotypes of WT, Lyz2^{Cre}, 14-3-3ε$^{f/f}$ and 14-3-3ε$^{f/f}$Lyz2^{Cre} mice were detected by PCR amplification on genomic DNA extracted from mouse tails. (B) Flow cytometric analysis of 14-3-3ε expression levels in peritoneal macrophages (CD11b^{+}F4/80^{+}),

neutrophils(CD11b$^+$LY6G$^+$), and DCs (CD11b$^+$CD11c$^+$) from CLP-modeled 14-3-3$\varepsilon^{f/f}$ and 14-3-3$\varepsilon^{f/f}$Lyz2^{Cre} mice. (C, D) Flow cytometry analysis of the proportions of neutrophil(CD11b$^+$LY6G$^+$) and DCs (CD11b$^+$CD11c$^+$) in peritoneal lavage fluid from CLP-modeled mice. Data are presented as mean ±SD and were analyzed using unpaired Student’s t-test (C, D). ns, nonsignificant.

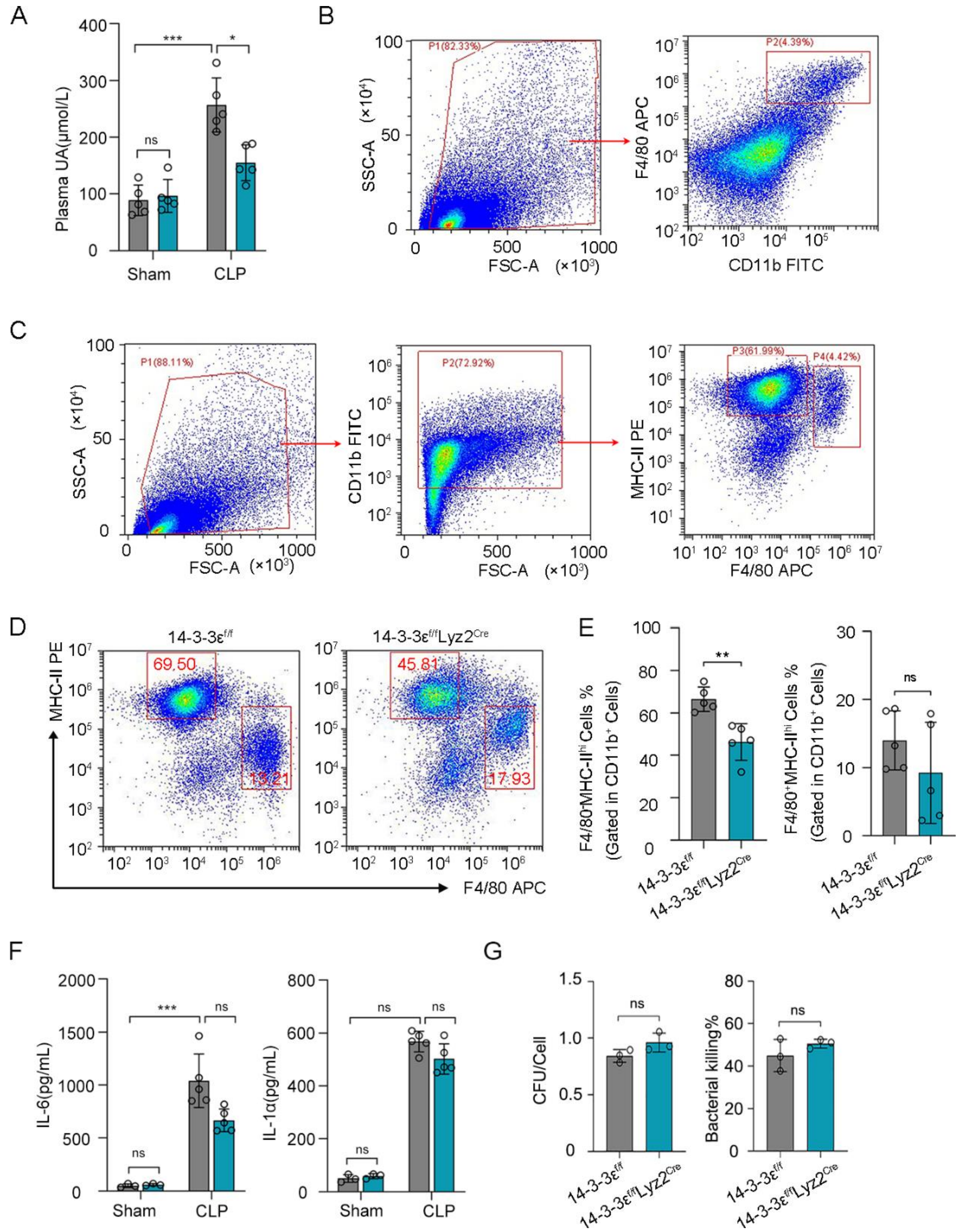

Supplemental Figure 8. 14-3-3$\varepsilon^{f/f}$Lyz2^{Cre} conditional knockout reduced small peritoneal macrophages. (A) UA in mice serum after 24 h of sham or CLP surgery was quantified using an automated biochemistry analyzer. (B) The gating strategy for macrophage (CD11b^{+}F4/80^{+}). (C) The gating strategy for large peritoneal macrophages (F4/80^{hi}MHC-II^{lo}) and small peritoneal macrophages (F4/80^{lo}MHC-II^{hi}). (D, E) Flow

cytometric analysis of large peritoneal macrophages and small peritoneal macrophages from the peritoneal cavity after 24 h of sham or CLP surgery. (F) IL-6 and IL-1α levels in mouse serum were measured by ELISA. (G) Efficiency of phagocytosis and intracellular killing of P. A. by BMDMs from 14-3-3$\varepsilon^{f/f}$ mice or 14-3-3$\varepsilon^{f/f}$Lyz2^{Cre} mice. Data are presented as mean ±SD and were analyzed using two-way ANOVA with Tukey’s multiple-comparison test (A, F) and unpaired Student’s t-test (E, G). ns, nonsignificant; *p<0.05; **p<0.01; ***p<0.001.

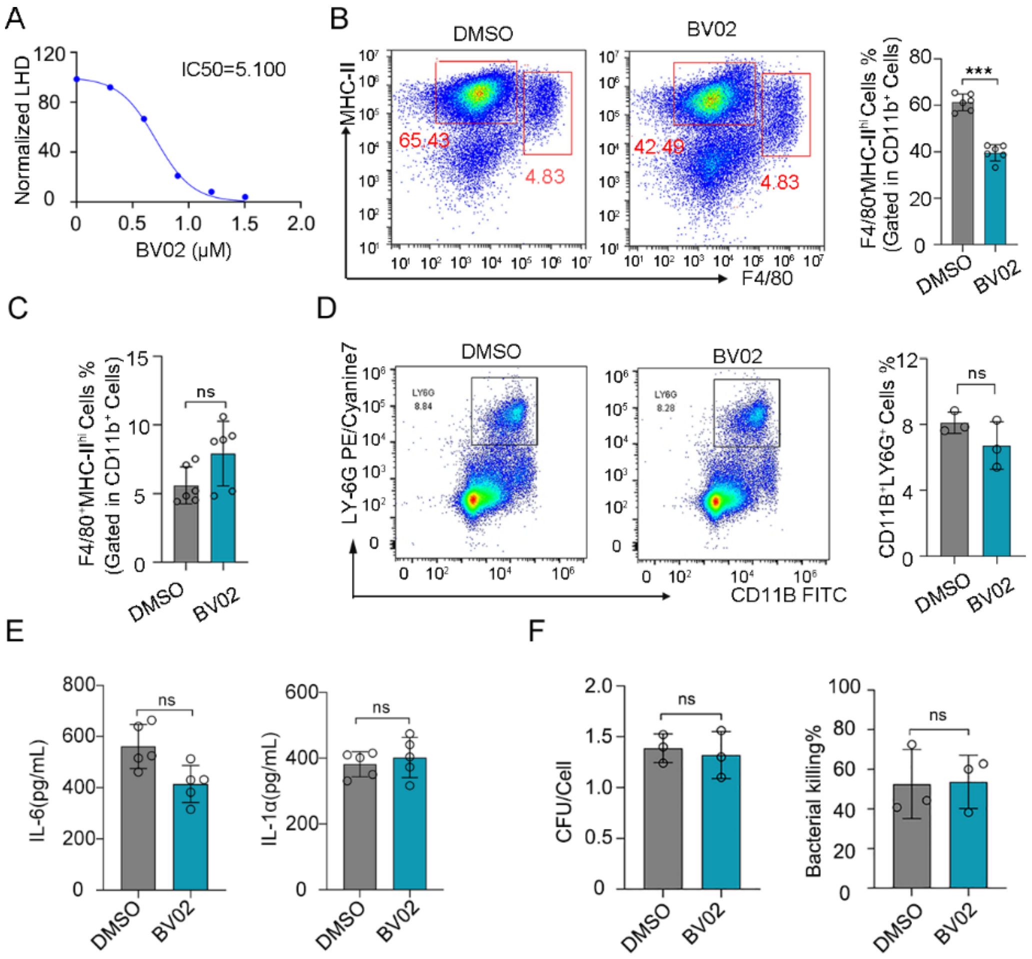

Supplemental Figure 9. BV02 treatment reduced small peritoneal macrophages but did not affect bacterial phagocytosis. (A) BV02 concentration–inhibition curves obtained for LDH release in BMDMs treated with LPS and Nig; BV02 IC50 was 5.1 μM. (B–D) Mice were treated with BV02 or DMSO (n=5 per group) at 12 h post-CLP, and peritoneal cells were analyzed 12 h after injection. Flow cytometry analysis of (B, C) large peritoneal macrophages and small peritoneal macrophages and (D) neutrophils ($CD11b^{+}Ly6G^{+}$). (E) IL-6 and IL-1α levels in mouse serum were measured by ELISA. (F) Efficiency of phagocytosis and intracellular killing of P. A. by DMSO treated- or BV02 treated- BMDMs. Data are presented as mean ±SD and were analyzed using unpaired Student's t-test (B–F). ns, nonsignificant; ***p<0.001.

Supplemental Table1. Top 20 bonded proteins in mass spectrometry analysis.

No.	Accession	-10lgP	Area N3Mock	Area N3Sepsis	#Peptides	Avg. Mass	Gene
1	Q8R4B8	259.94	1.07E+08	1.89E+08	28	118275	Nlrp3
2	P48678	232.48	1.72E+07	2.17E+07	18	74238	Lmna
3	P35700	231.81	1.13E+08	1.10E+08	14	22176	Prdx1
4	Q8VDD5	227.9	8.68E+06	4.62E+06	29	226370	Myh9
5	P20029	223.12	6.16E+07	7.81E+07	14	72422	Hspa5
6	P62259	217.96	5.38E+07	6.83E+07	14	29174	Ywhae
7	P38647	206.3	2.37E+07	3.80E+07	12	73461	Hspa9
8	E9Q555	203.64	9.67E+06	1.02E+07	23	584265	Rnf213
9	P29341	199.27	1.84E+07	2.05E+07	13	70671	Pabpc1
10	O70133	197.01	3.18E+06	6.24E+06	11	149474	Dhx9
11	P52480	192.76	2.67E+07	2.82E+07	12	57845	Pkm
12	Q99P91	191.89	2.57E+07	1.95E+07	8	63676	Gpnmb
13	Q8VDP4	188.62	1.61E+07	1.66E+07	10	103002	Ccar2
14	P62960	184.96	8.93E+06	1.14E+07	5	35730	Ybx1
15	P62908	182.33	2.19E+07	3.06E+07	9	26674	Rps3
16	P54987	182.06	1.19E+07	2.08E+07	9	53759	Acod1
17	Q03265	178.72	8.85E+06	1.24E+07	10	59753	Atp5f1a
18	Q9JKF1	176.96	4.39E+06	5.49E+06	9	188741	Iqgap1
19	P99024	176.6	2.30E+06	2.06E+06	9	49671	Tubb5
20	P63017	175.27	6.58E+06	8.55E+06	10	70871	Hspa8

Supplemental Table2 The characteristics of healthy controls and sepsis patients

**Variables**	**Healthy controls**	**Sepsis Patients**
Sample size (no.)	29	60
Sex-no. (%)		
Male	15 (51.72)	30 (50)
Female	14 (48.28)	30 (50)
Age (years)		
Mean ±SD	62.69 ±11.42	66.65 ±14.44
Median (IQR)	61.0 (54, 70)	68.0(59.75, 72.25)
Range	42-90	21-96
Blood cells		
Platelets ($\times 10^9$/ L)	257.83±53.16	184.53±148.49
Granulocyte($\times 10^9$/ L)	6.43±1.37	16.37 ±7.83
Lymphocyte ($\times 10^9$/ L)	2.11±0.60	0.65 ±0.27
Serum biochemical indexes		
Fibrinogen (g/L)	3.81±0.72	5.03±1.57
CRP (mg/L)	/	149.39±91.54
ALT (U/L)	17.96±9.93	55.9±84.32
Urea(mmol/L)	6.03±5.41	12.55±8.93
PT (s)	13.27±1.49	18.20±9.4

Abbreviations: CRP, C-reactive protein; PT, prothrombin time; ALT, alanine aminotransferase. All data are shown as Mean ±SD.

Supplemental Table3 Sequences of q-PCR primers and siRNA.

Primer	Sequence
Primer: *nlrp3*Forward	TCACAACTCGCCCAAGGAGGAA
Primer: *nlrp3* Reverse	AAGAGACCACGGCAGAAGCTAG
Primer: *il-1b*Forward	TGGACCTTCCAGGATGAGGACA
Primer: *il-1b* Reverse	GTTCATCTCGGAGCCTGTAGTG
Primer: *caspase1* Forward	GGCACATTTCCAGGACTGACTG
Primer: *caspase1* Reverse	GCAAGACGTGTACGAGTGGTTG
Primer: *asc* Forward	CTGCTCAGAGTACAGCCAGAAC
Primer: *asc* Reverse	CTGTCCTTCAGTCAGCACACTG
Primer: *beta actin* Forward	GATTACTGCTCTGGCTCCTAGC
Primer: *beta actin* Reverse	GACTCATCGTACTCCTGCTTGC
14-3-3ε-siRNA	UGUACAUCCAGAAUGUCACAACAGA

References

1. Ming S, Li X, Xiao Q, Qu S, Wang Q, Fang Q, et al. TREM2 aggravates sepsis by inhibiting fatty acid oxidation via the SHP1/BTK axis. *J Clin Invest.* 2024;135(1).
2. Wu Y, Wang Q, Li M, Lao J, Tang H, Ming S, et al. SLAMF7 regulates the inflammatory response in macrophages during polymicrobial sepsis. *The Journal of clinical investigation.* 2023;133(6).
3. Li X, Zhou G, Sun X, Qu S, Lai H, Wu Y, et al. NLRP12 Senses the SARS-CoV-2 Membrane Protein and Promotes an Inflammatory Response. *J Infect Dis.* 2024;229(3):660-70.
4. Li X, Dong Z, Liu Y, Song W, Pu J, Jiang G, et al. A Novel Role for the Regulatory Nod-Like Receptor NLRP12 in Anti-Dengue Virus Response. *Front Immunol.* 2021;12:744880.
5. MM LHHKKJ. SYNCRIP (synaptotagmin-binding, cytoplasmic RNA-interacting protein) is a host factor involved in hepatitis C virus RNA replication. *Virology.* 2009;386(2).
6. Liu HM, Loo YM, Horner SM, Zornetzer GA, Katze MG, and Gale M, Jr. The mitochondrial targeting chaperone 14-3-3ε regulates a RIG-I translocon that mediates membrane association and innate antiviral immunity. *Cell Host Microbe.* 2012;11(5):528-37.

7. Zhou R, Yazdi AS, Menu P, and Tschopp J. A role for mitochondria in NLRP3 inflammasome activation. *Nature.* 2011;469(7329):221-5.

8. Wieckowski MR, Giorgi C, Lebiedzinska M, Duszynski J, and Pinton P. Isolation of mitochondria-associated membranes and mitochondria from animal tissues and cells. *Nat Protoc.* 2009;4(11):1582-90.
