# Supplementary material for: 14-3-3ε–dependent deubiquitination and translocation of NLRP3 activates the inflammasome during sepsis
Source: JCI Insight. 2026 Jan 9;11(1):e192970. doi: 10.1172/jci.insight.192970 (PMC12890513; doi:10.1172/jci.insight.192970)

Figure1E

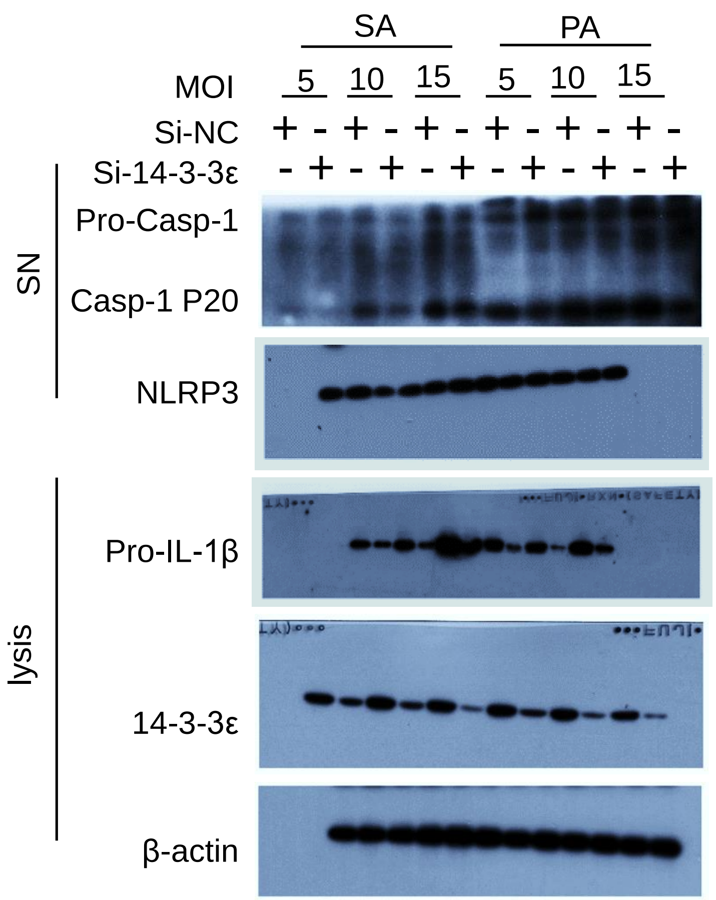

Figure1G

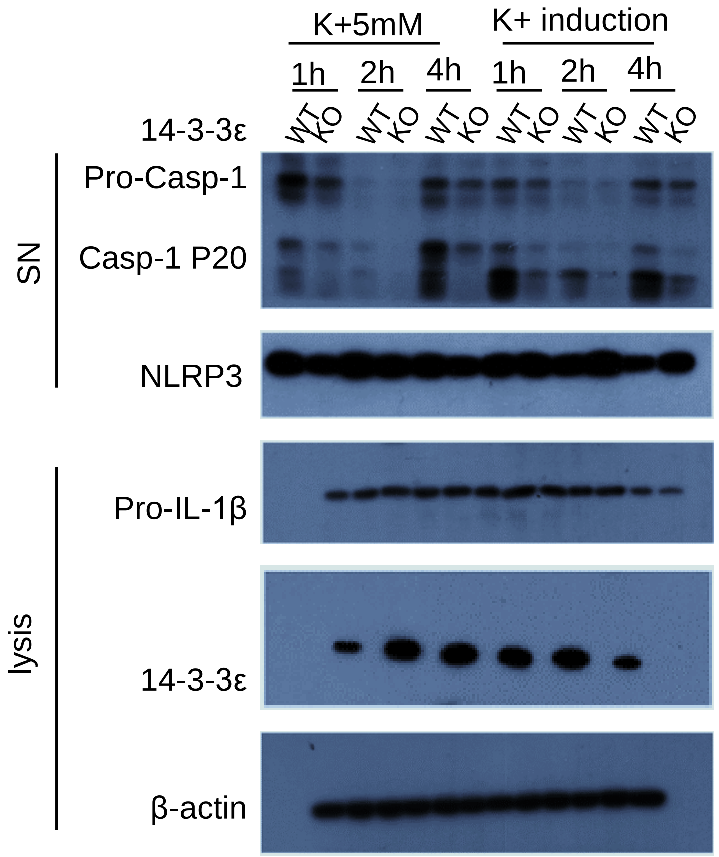

Figure1I

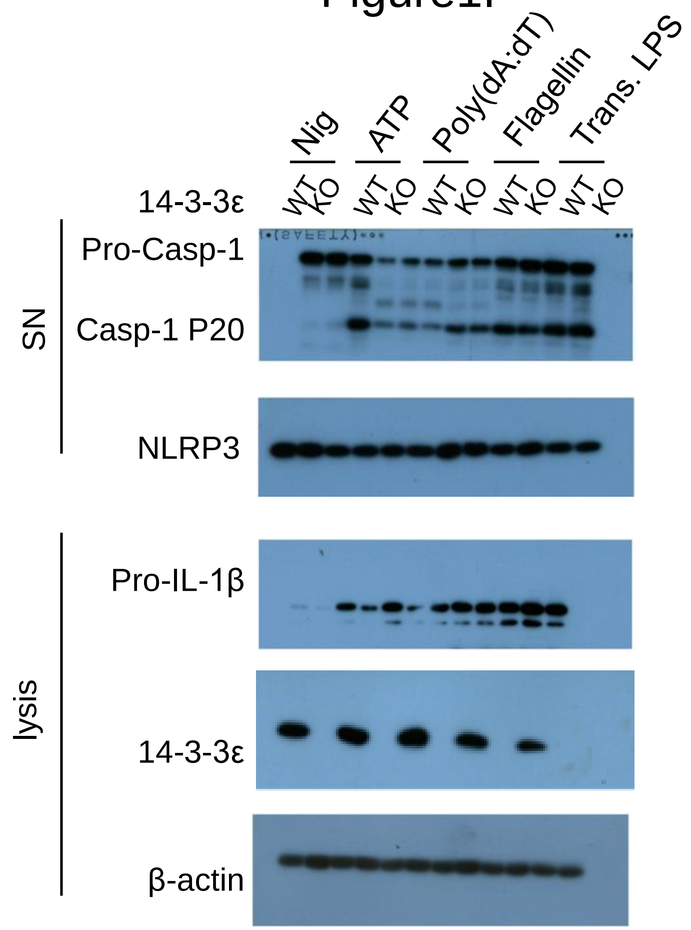

Figure1K

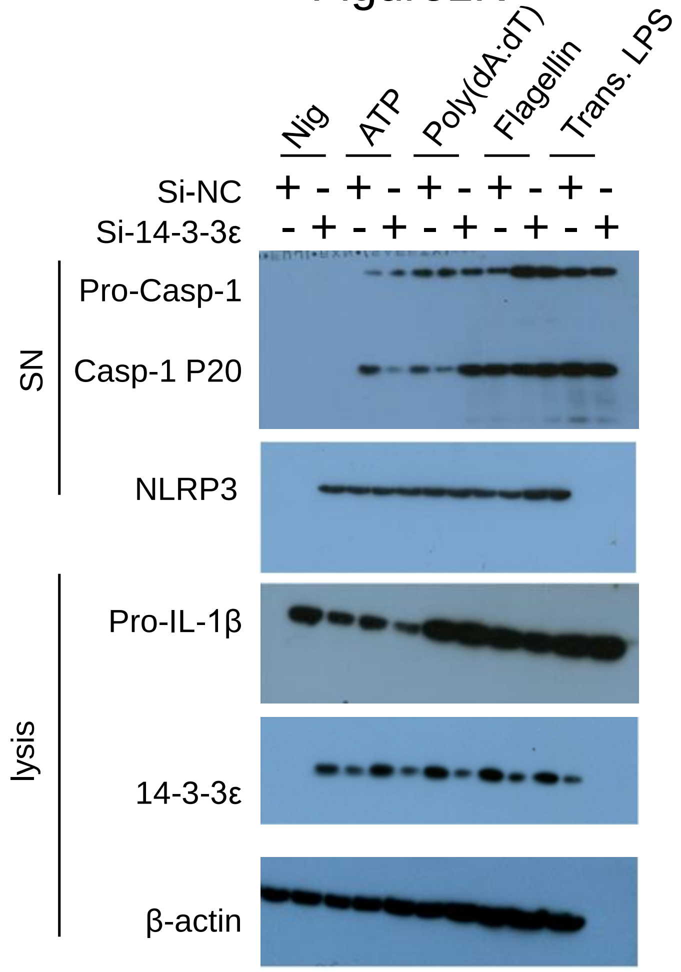

Figure 2A

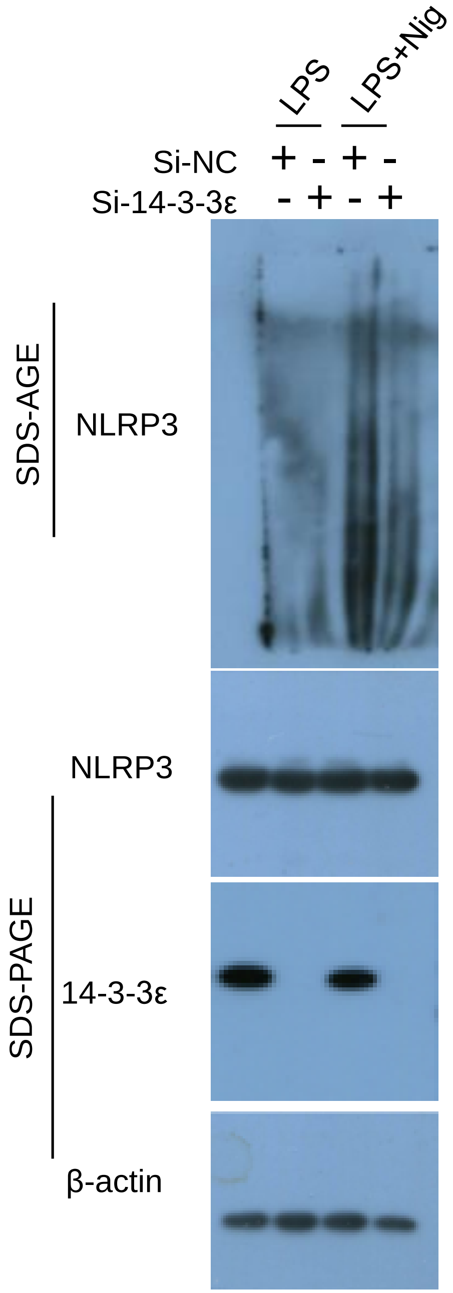

Figure 2B

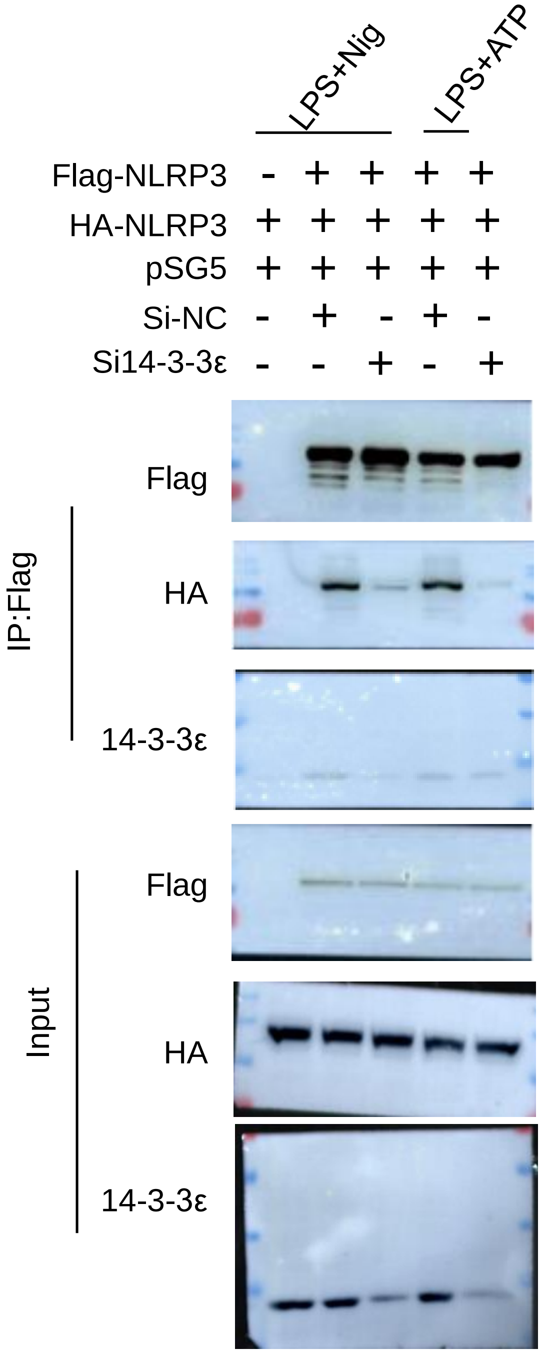

Figure 2D

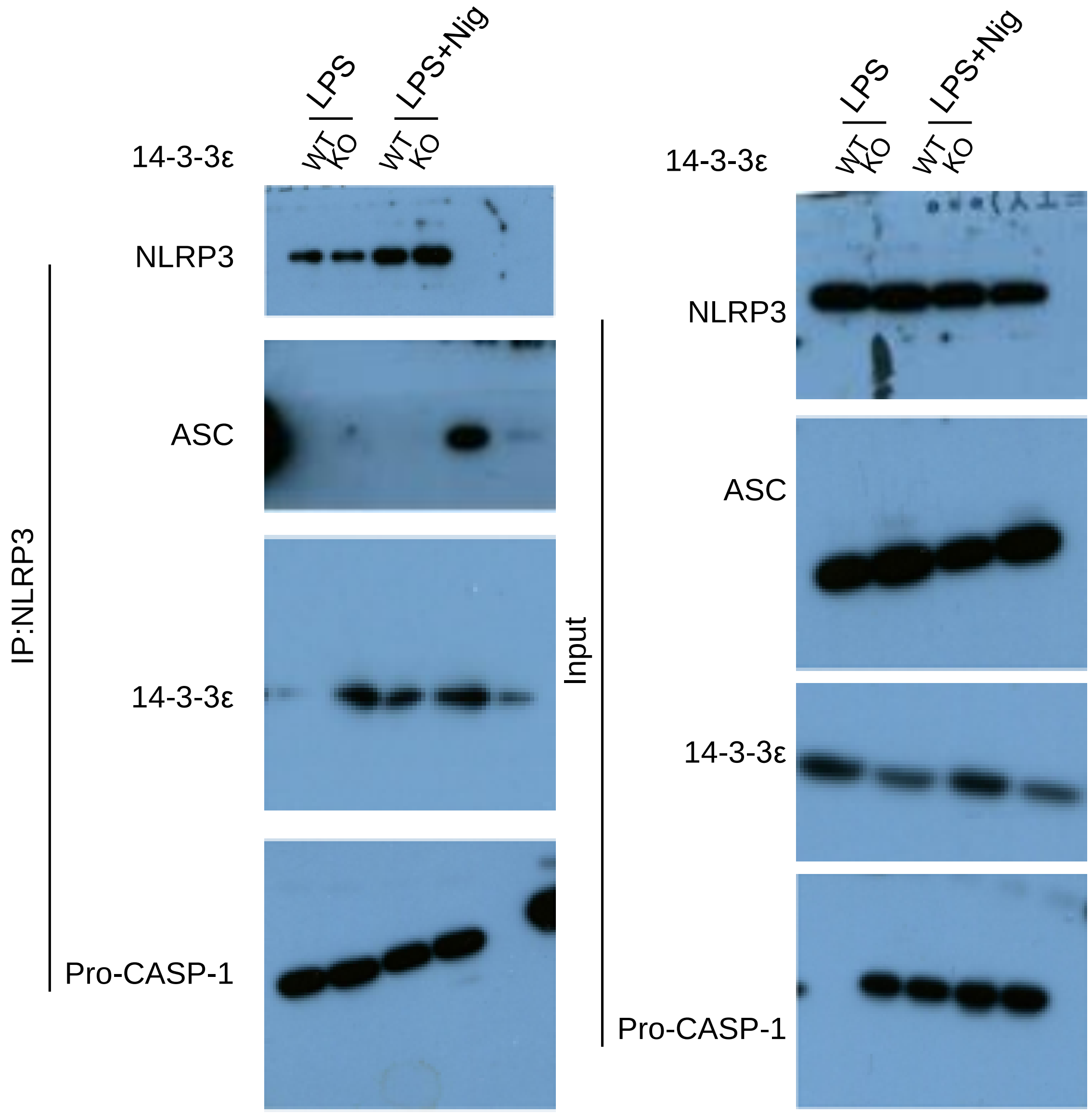

Figure 2E

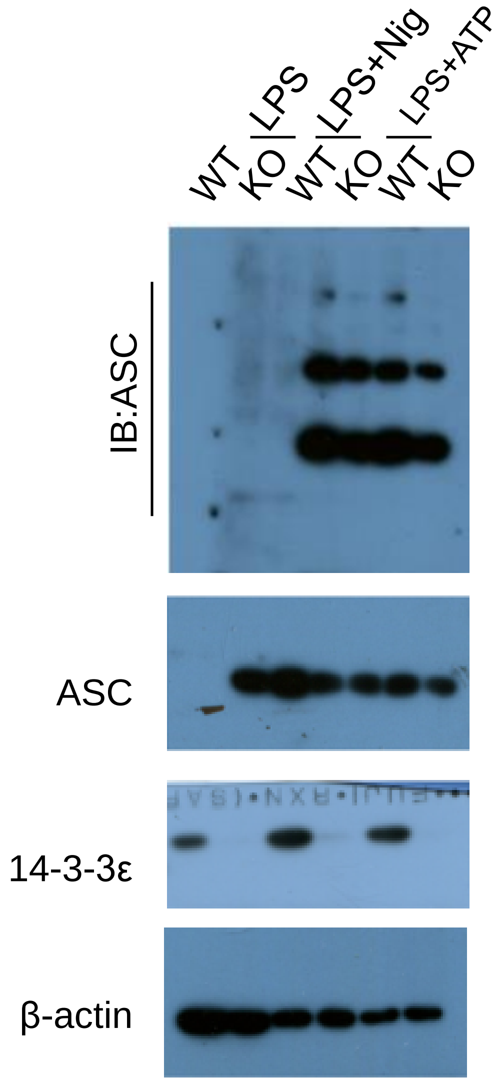

Figure 3A

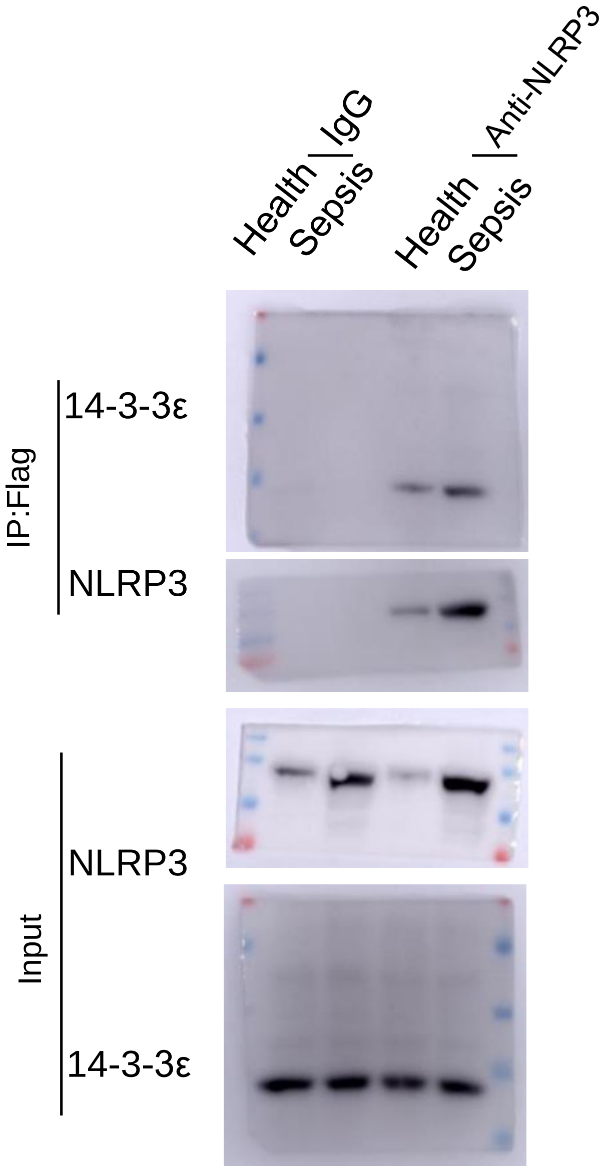

Figure 3B

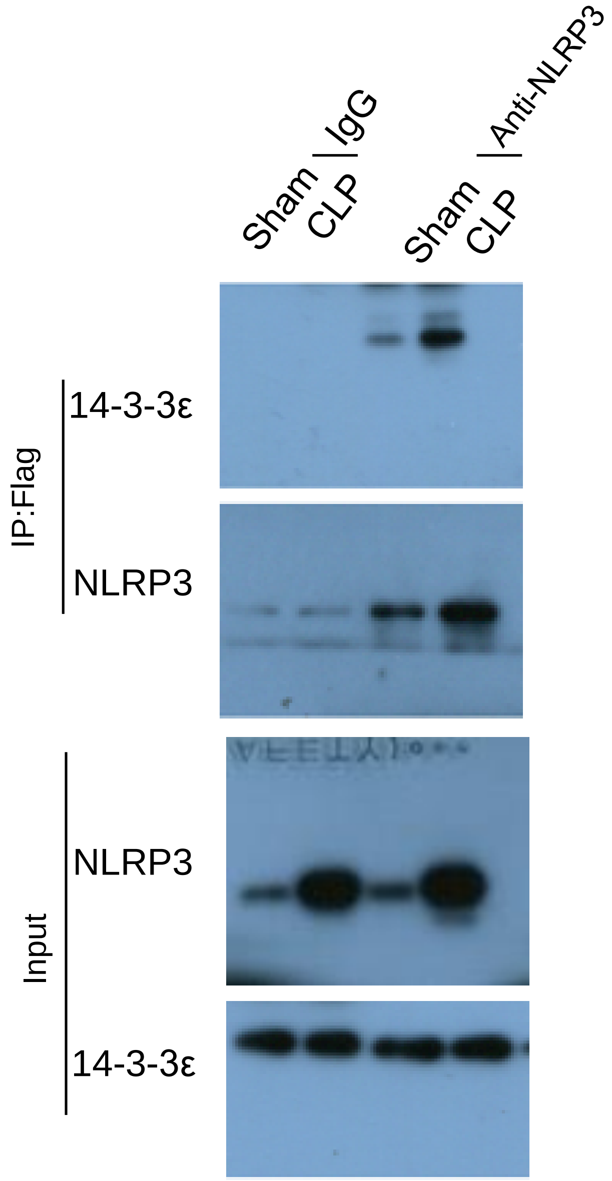

Figure 3E

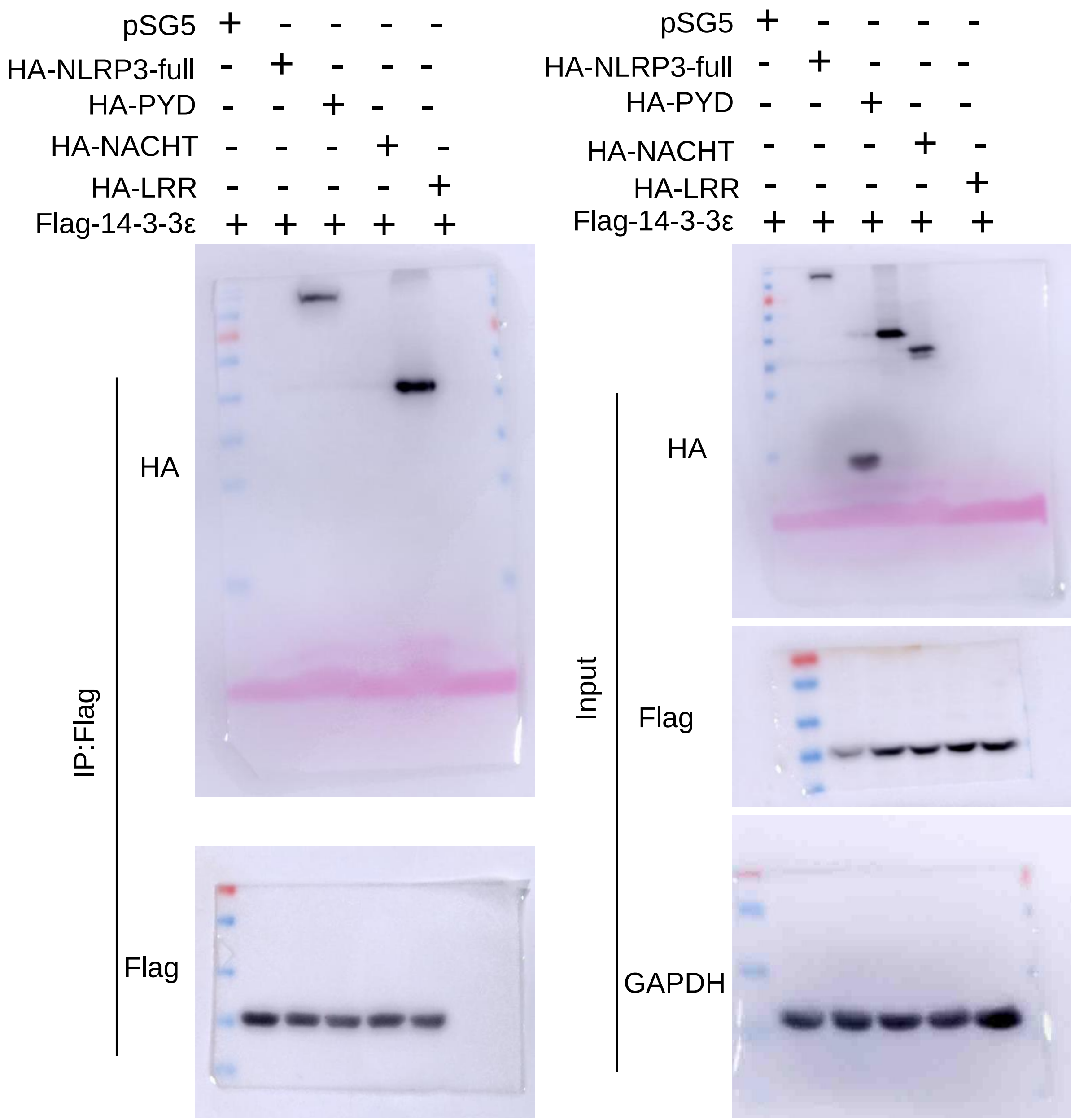

Figure 3F

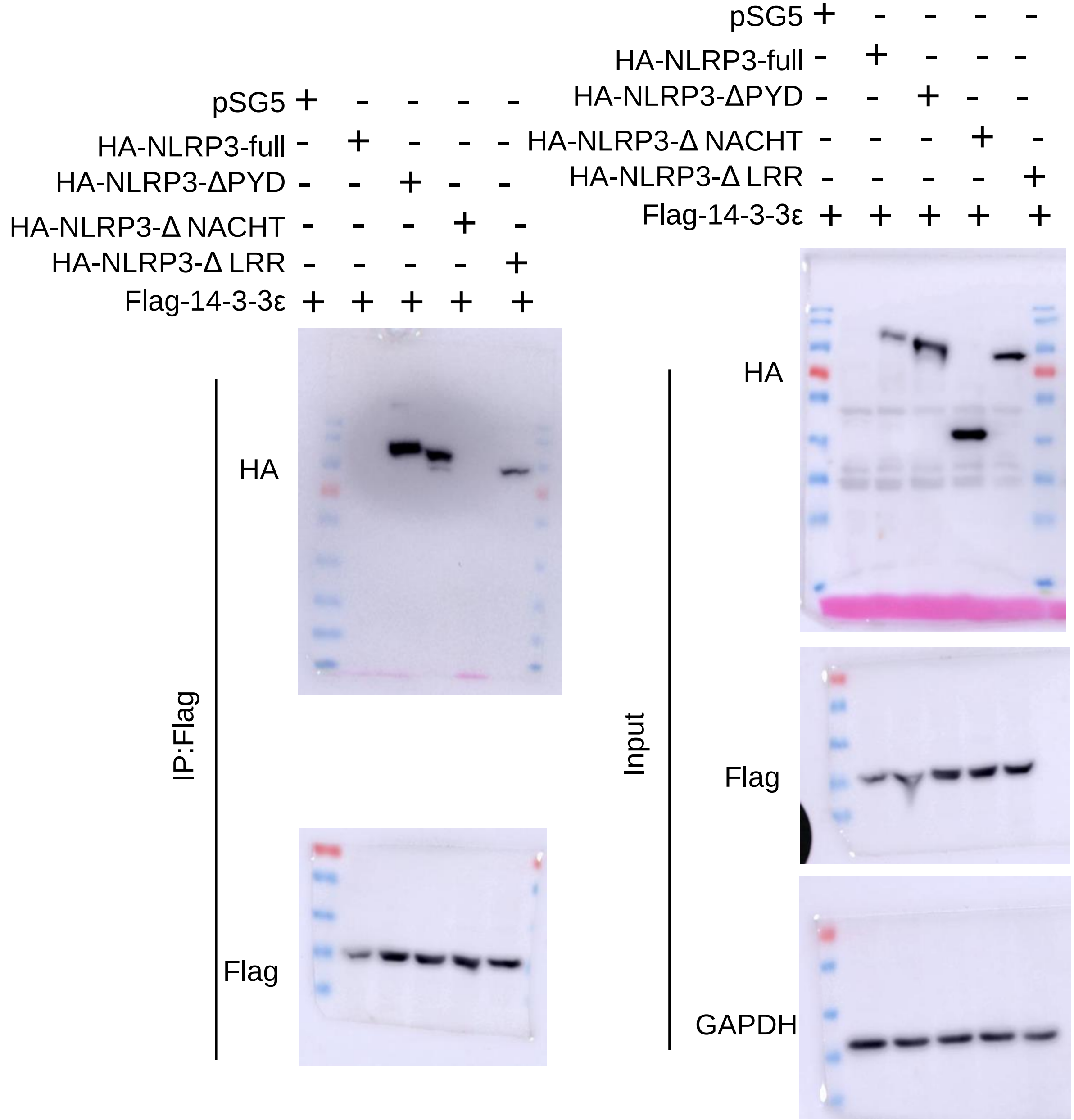

Figure 4B

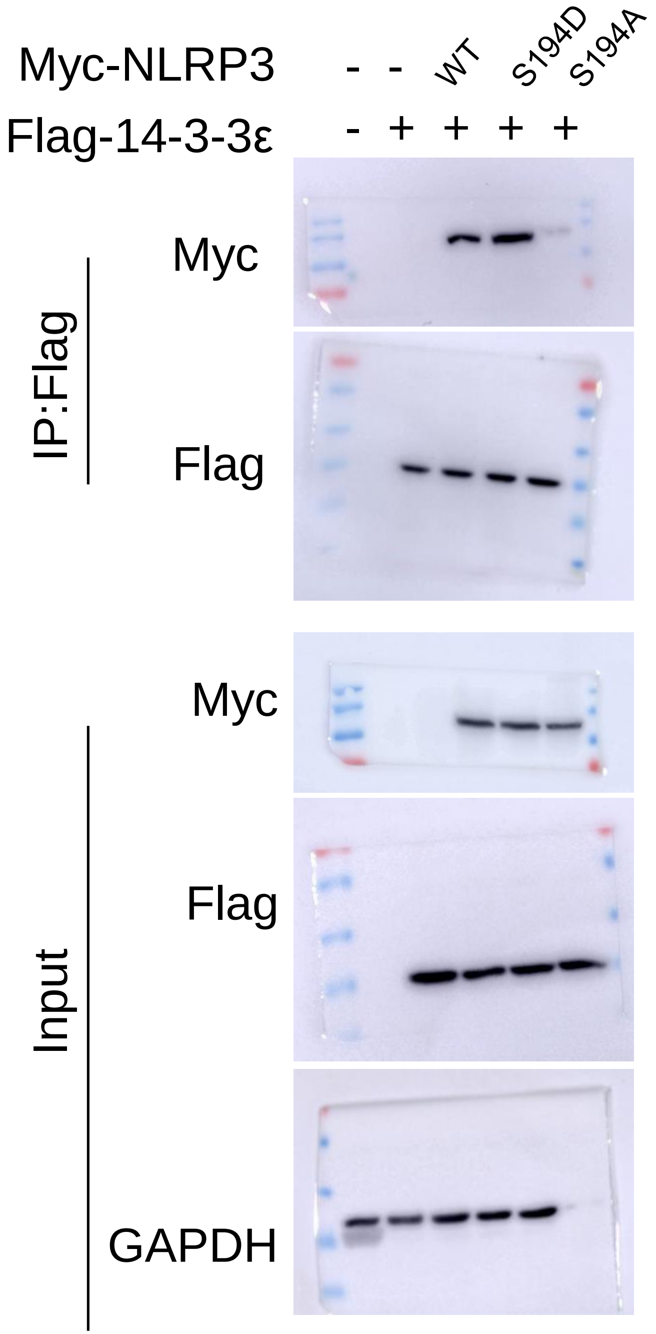

Figure 4E

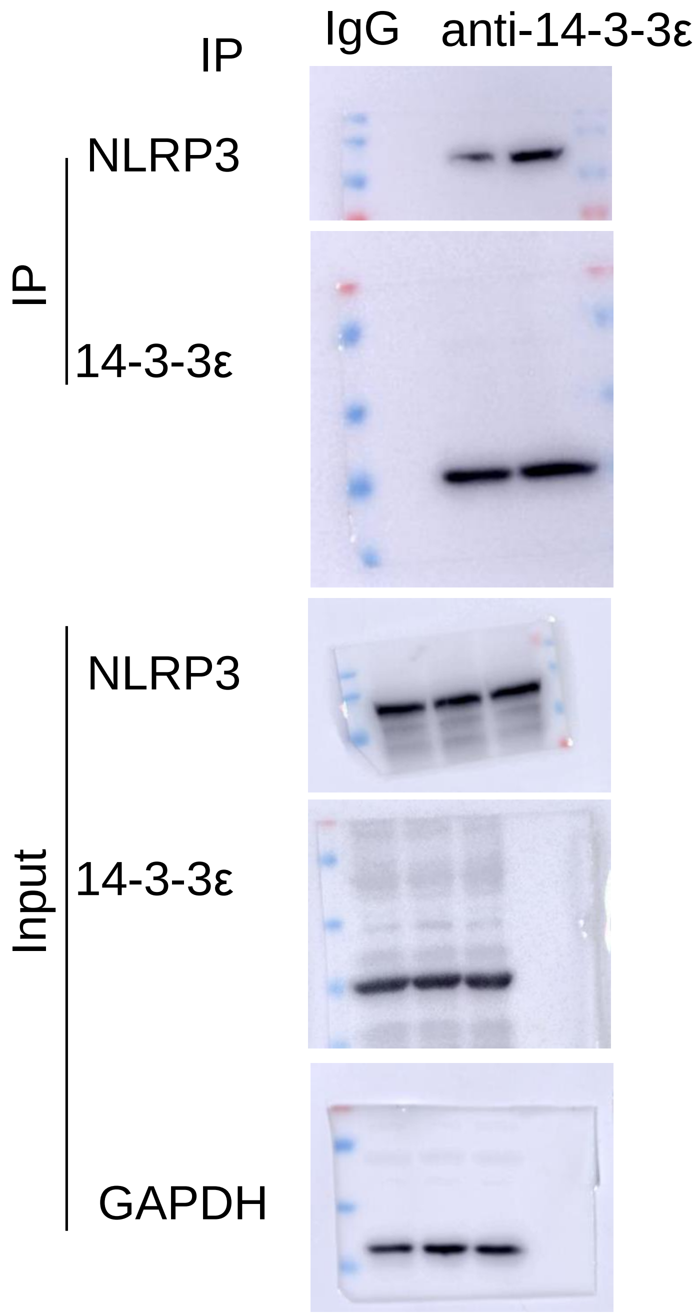

Figure 4F

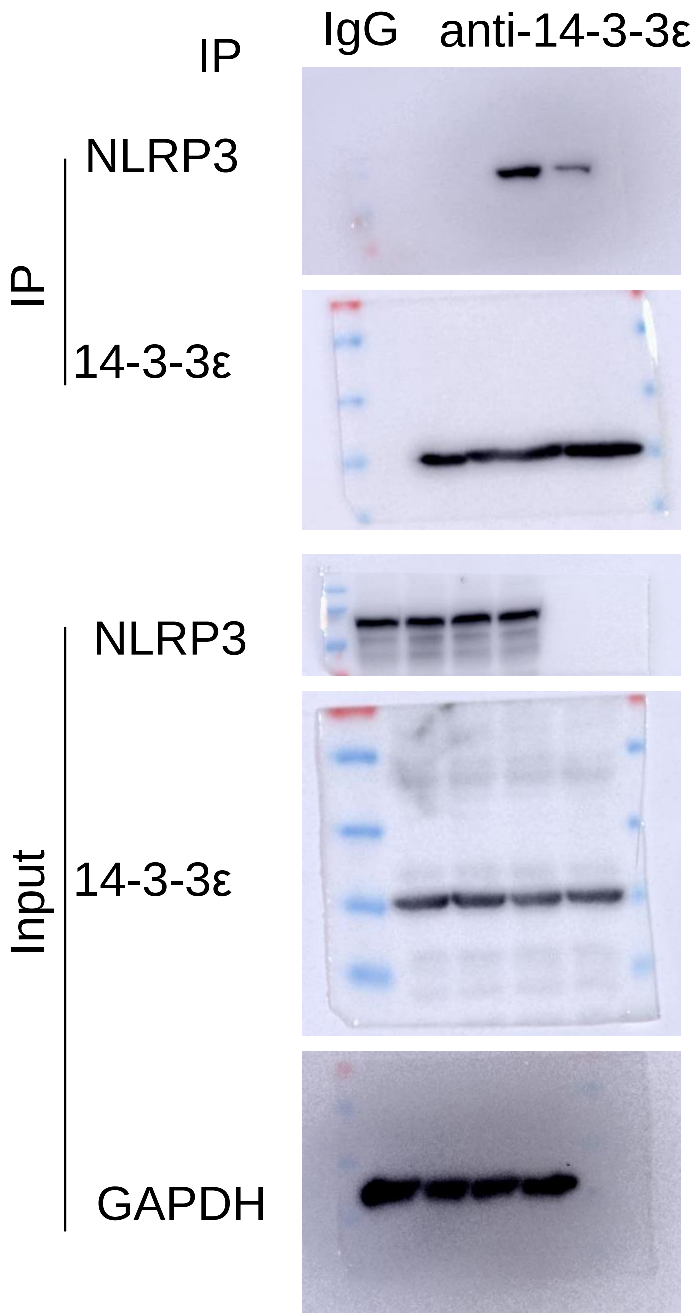

Figure 4G

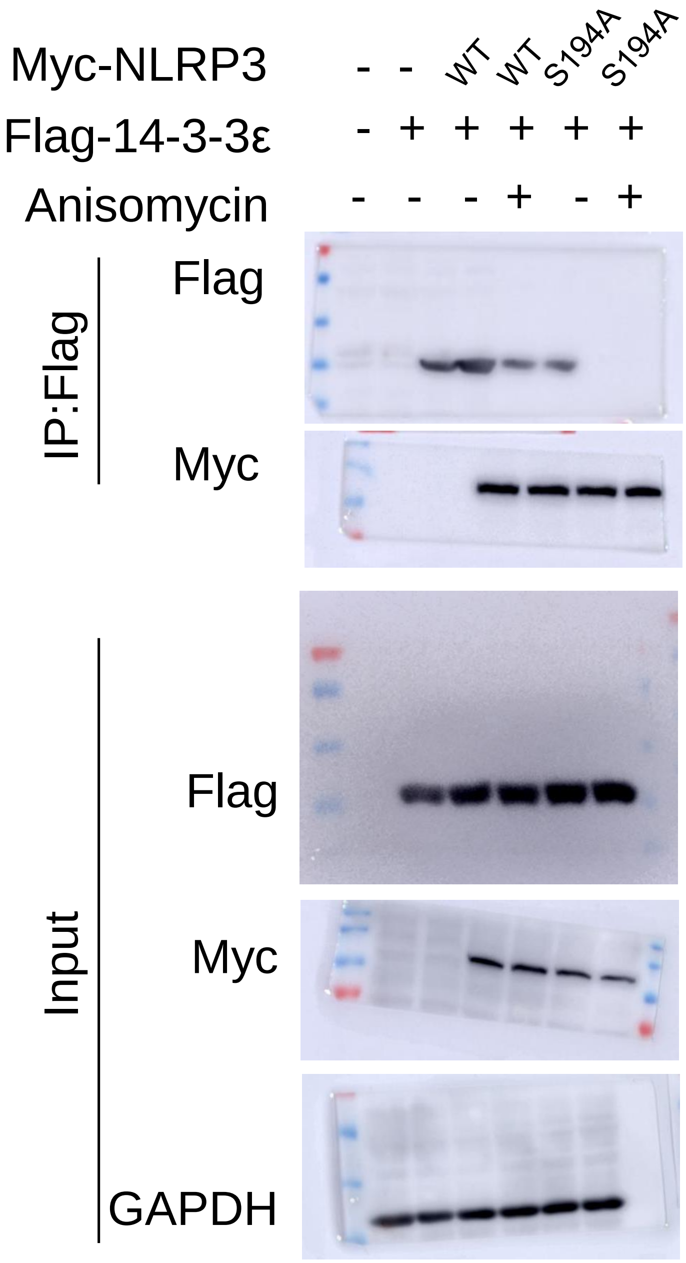

Figure 4G

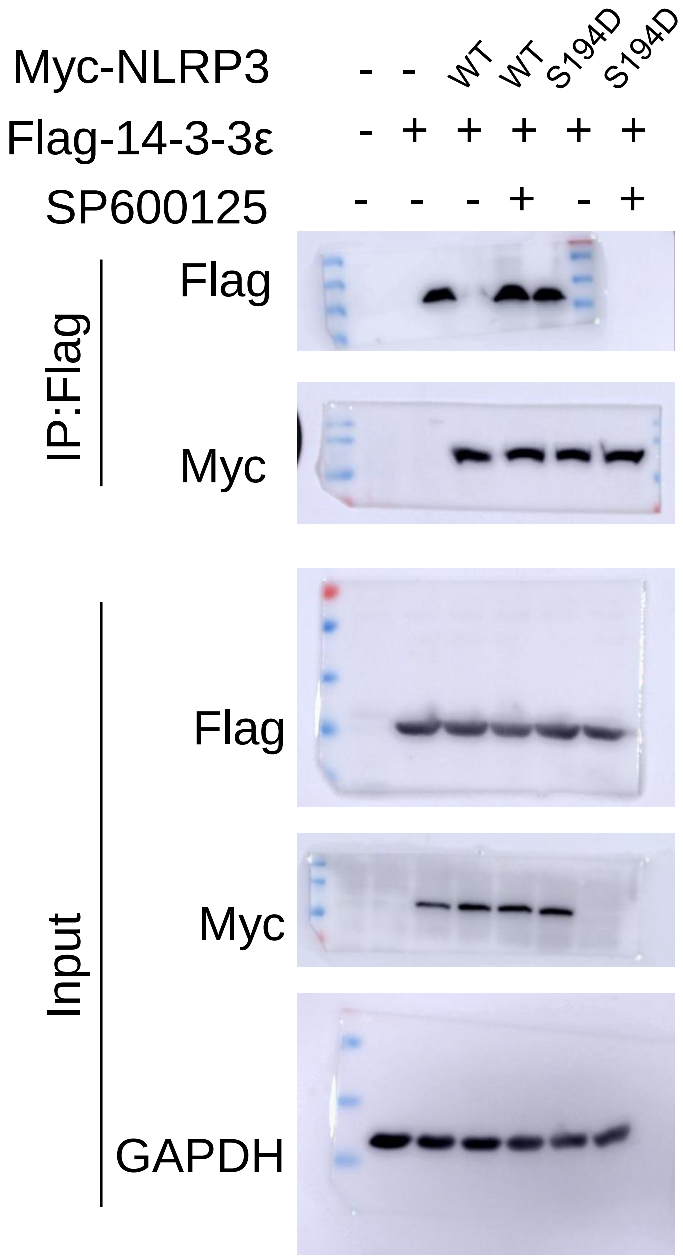

Figure 5A

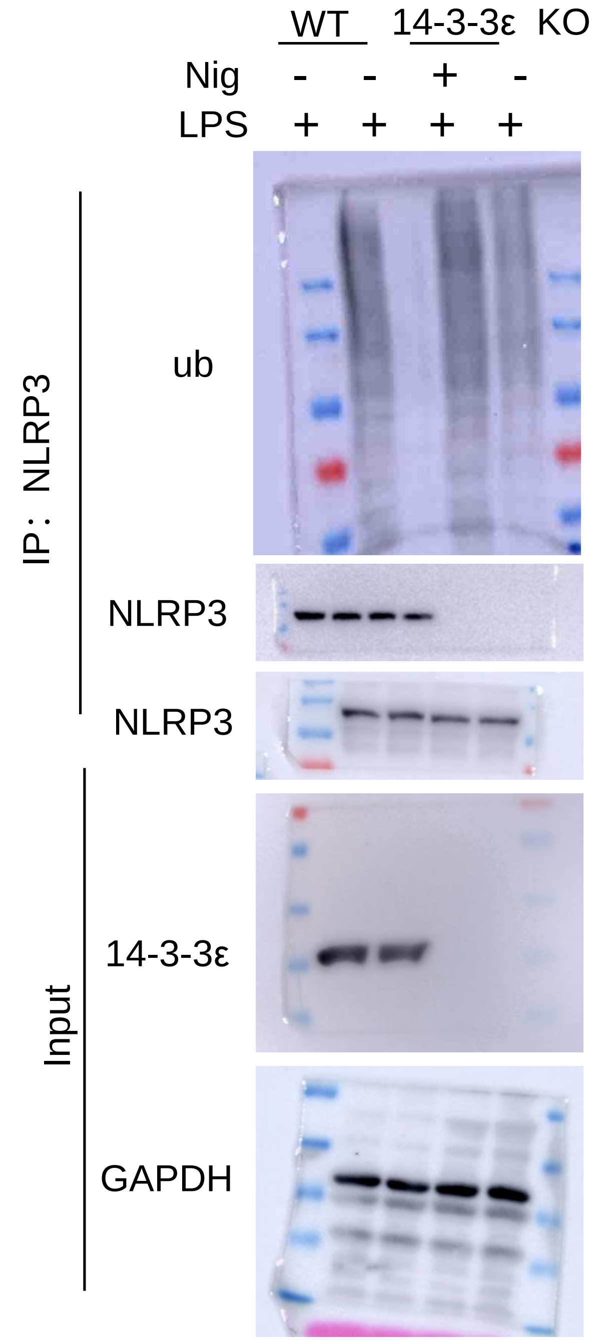

Figure 5B

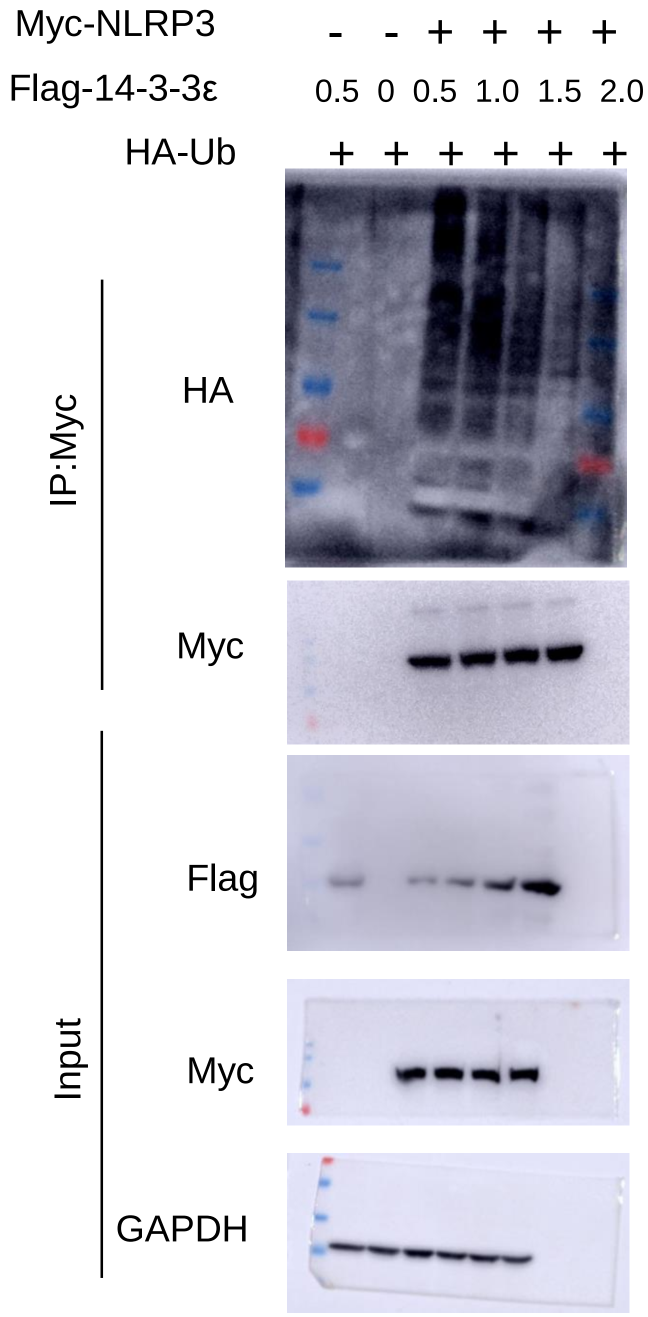

Figure 5C

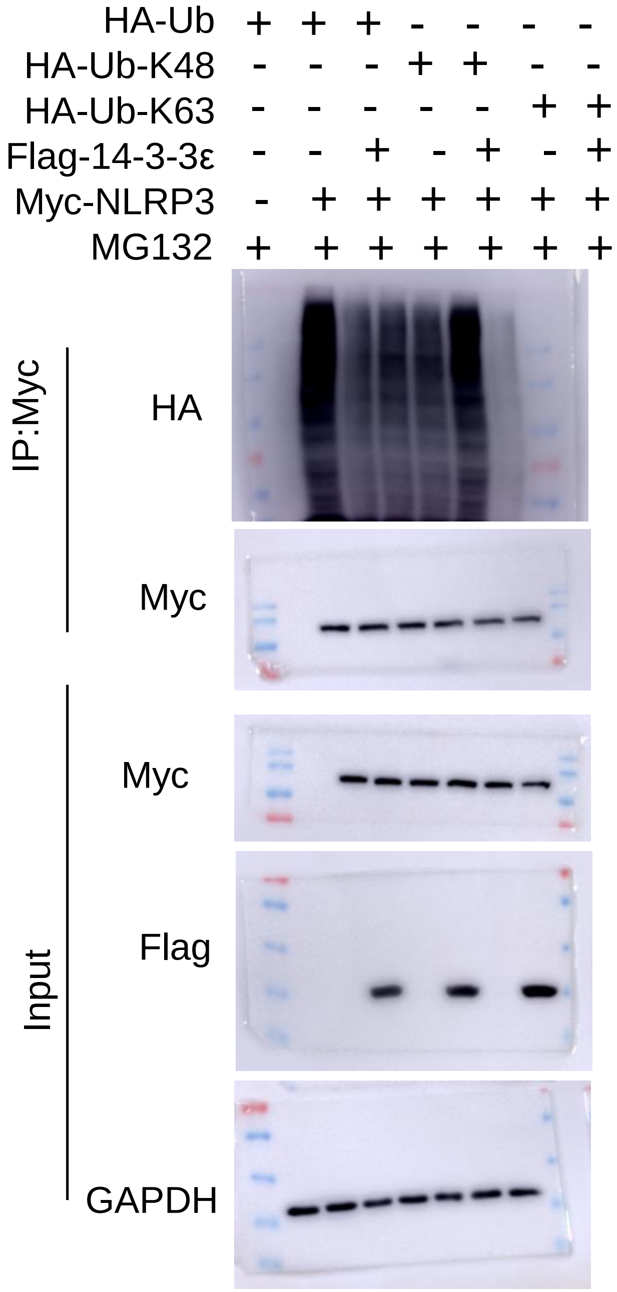

Figure 5D

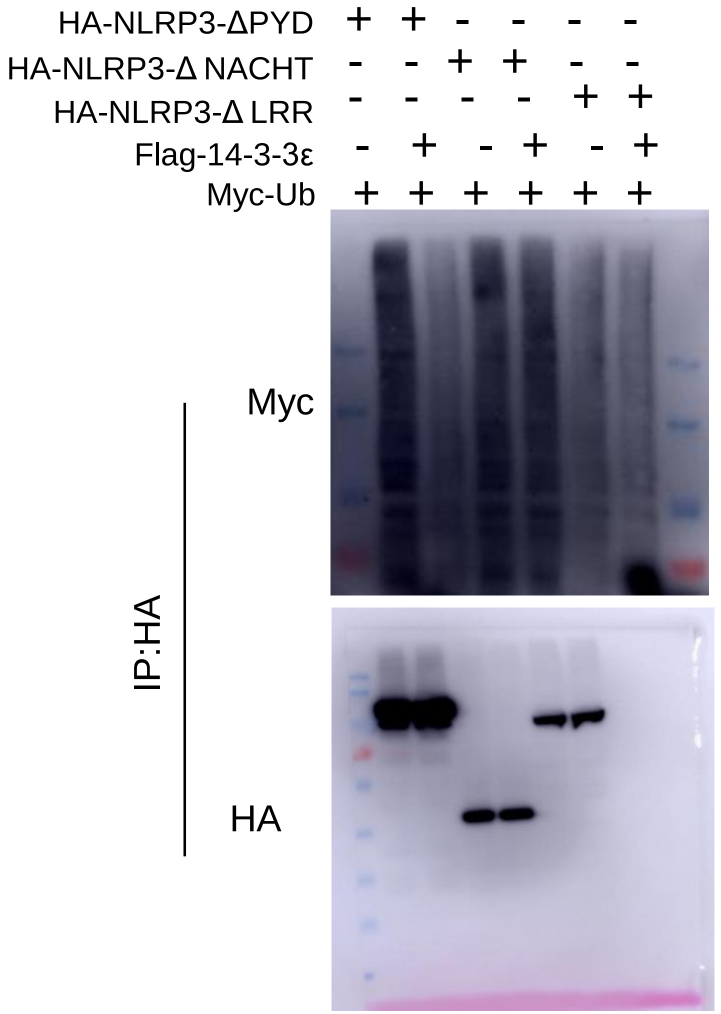

Figure 5D

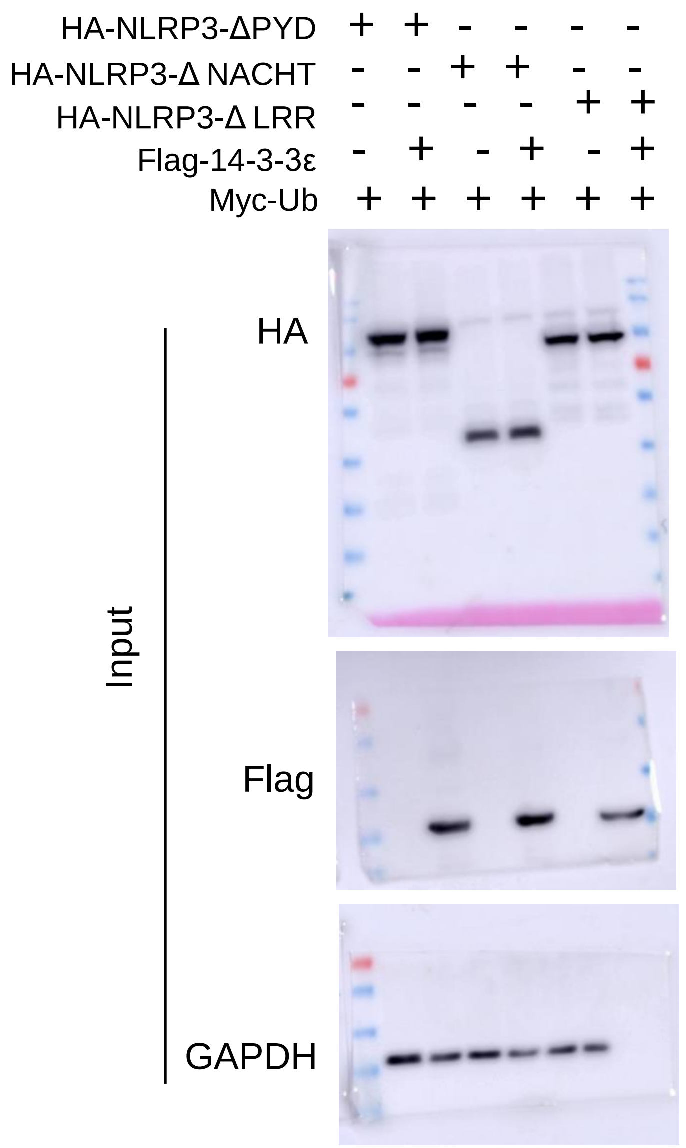

Figure 5E

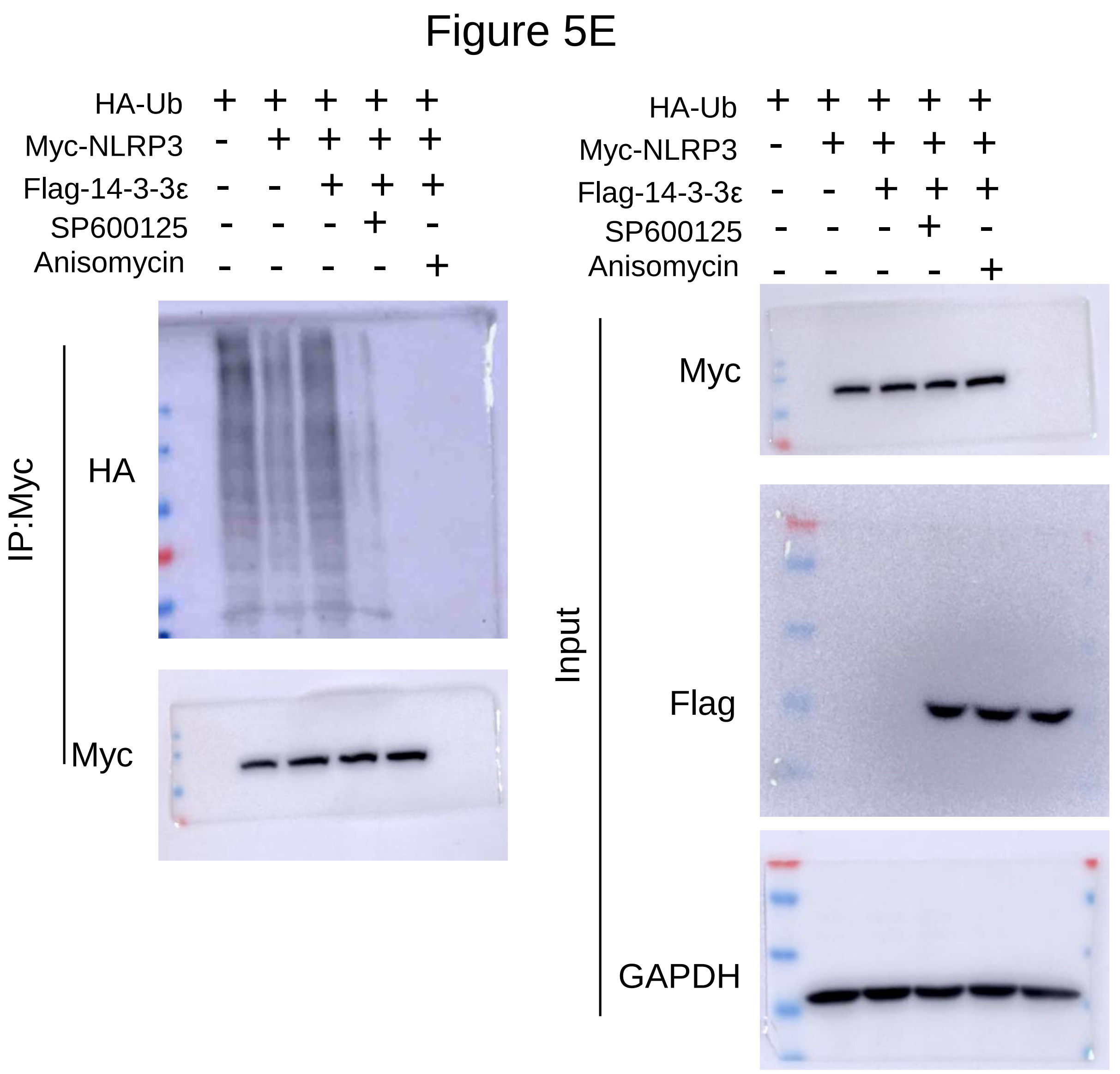

Figure 5F

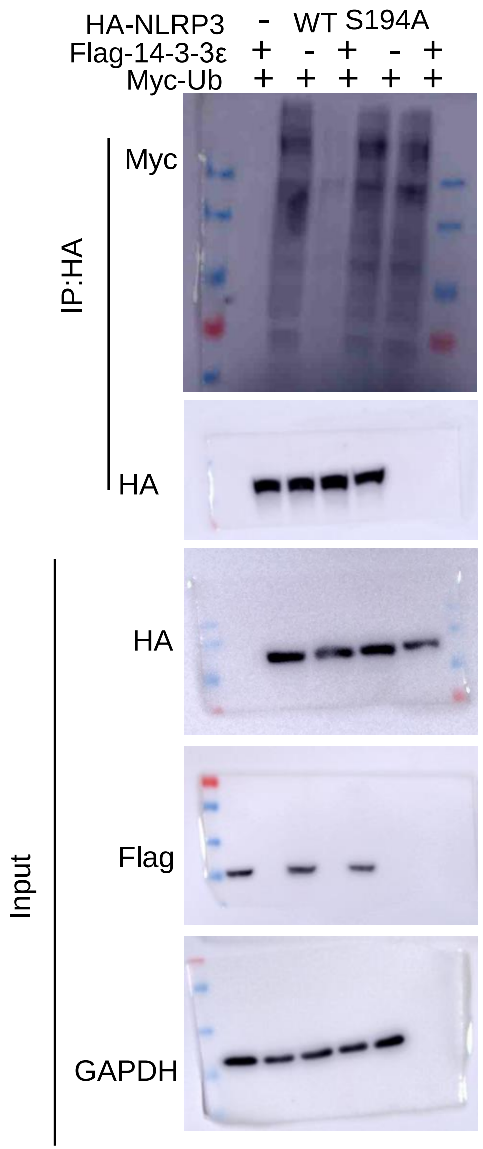

Figure 6A

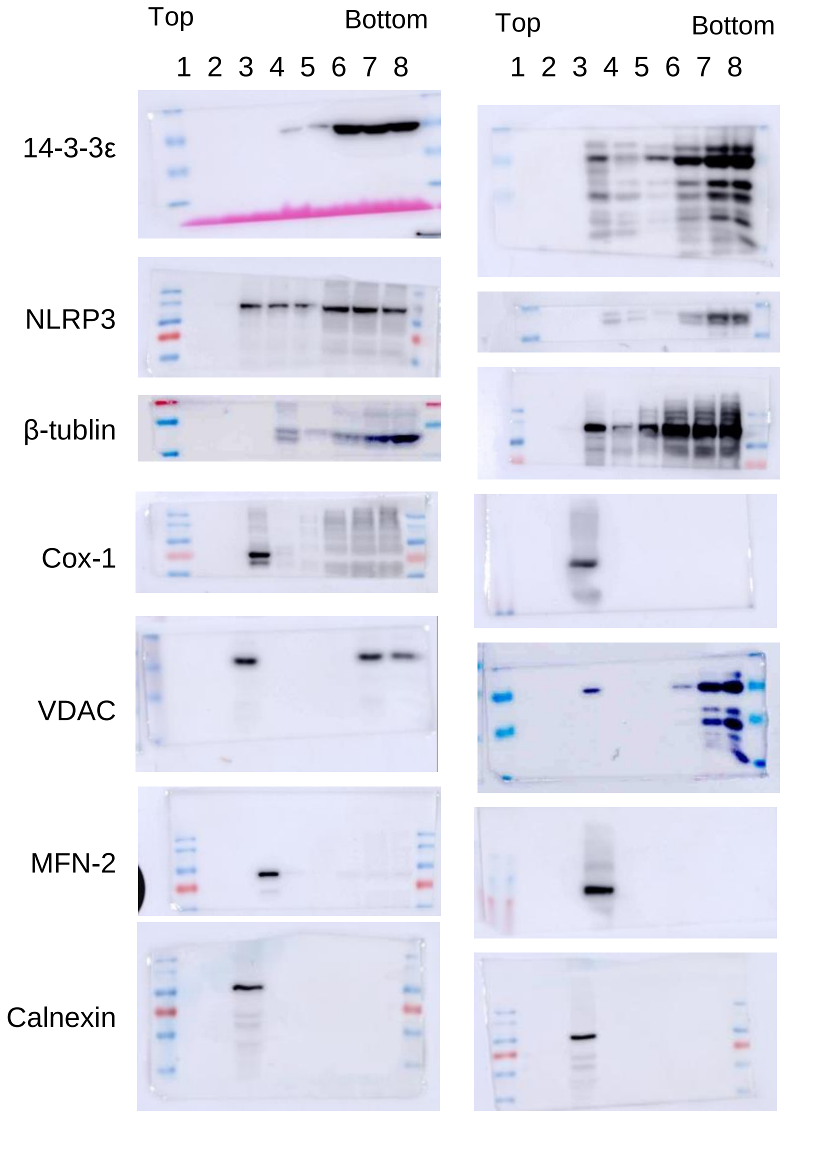

Figure 6B

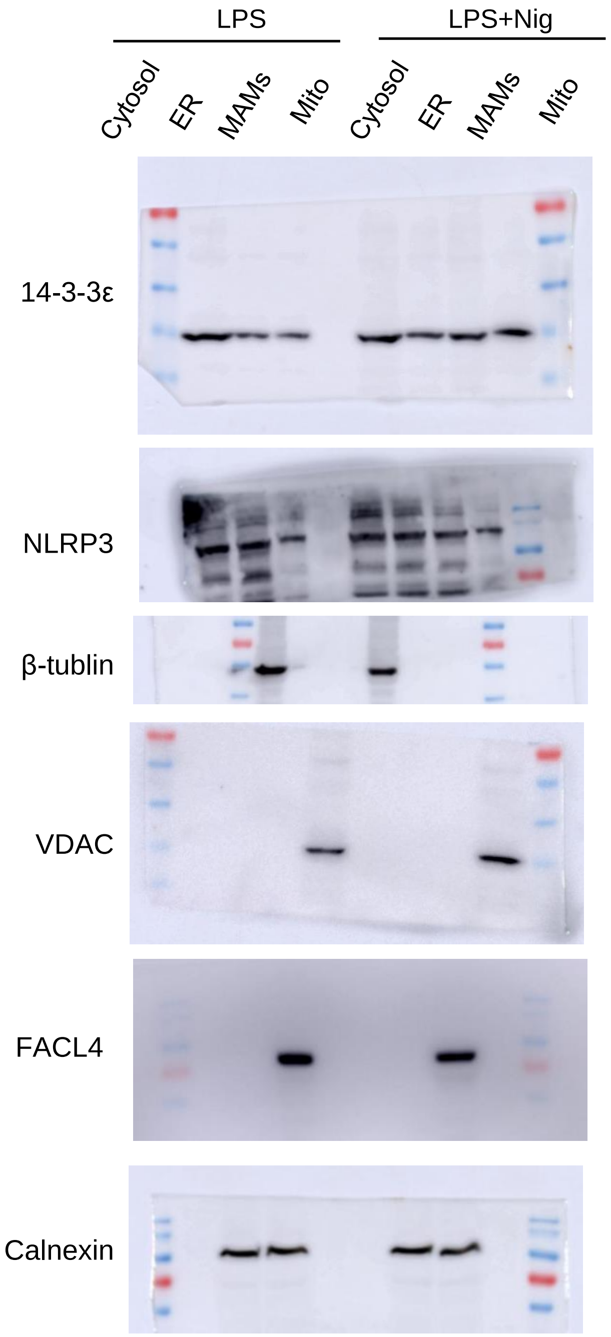

Figure 6F

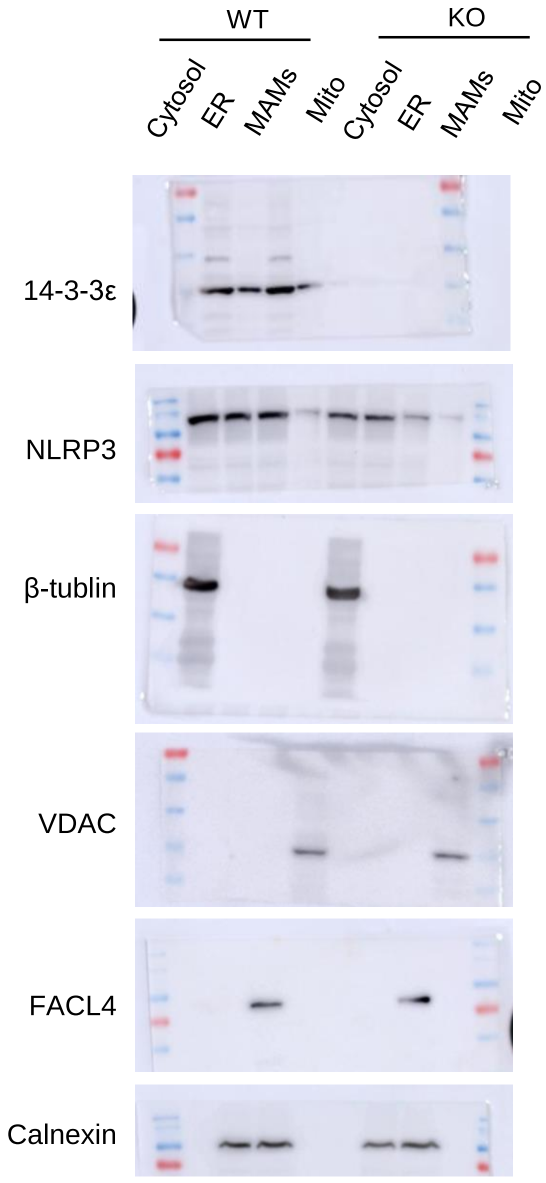

Figure 6G

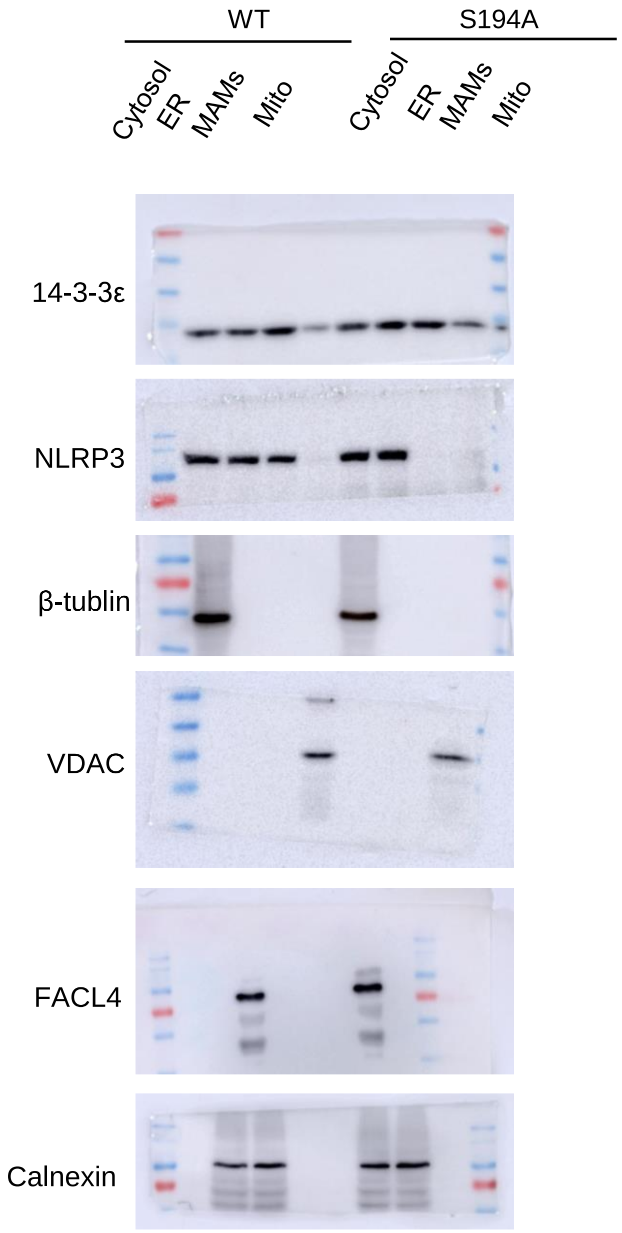

Figure S1A

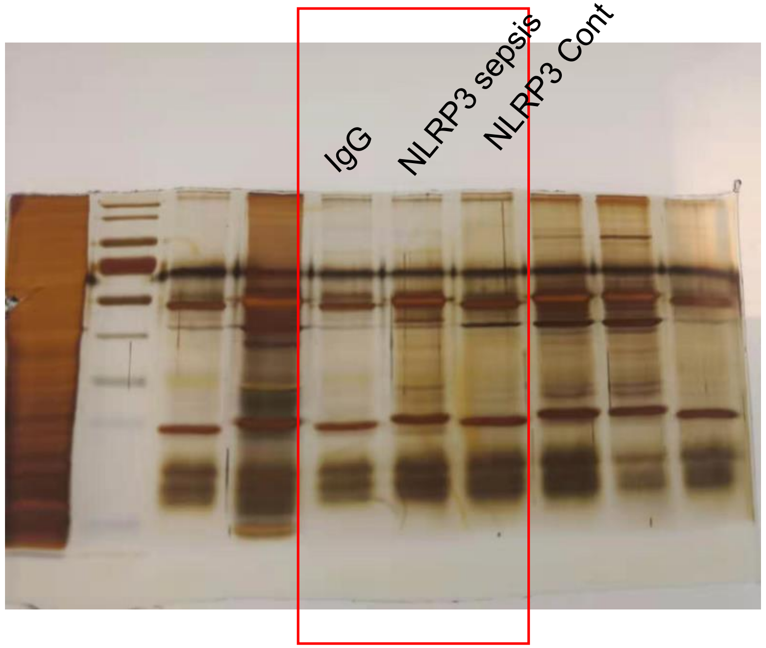

Figure S2A

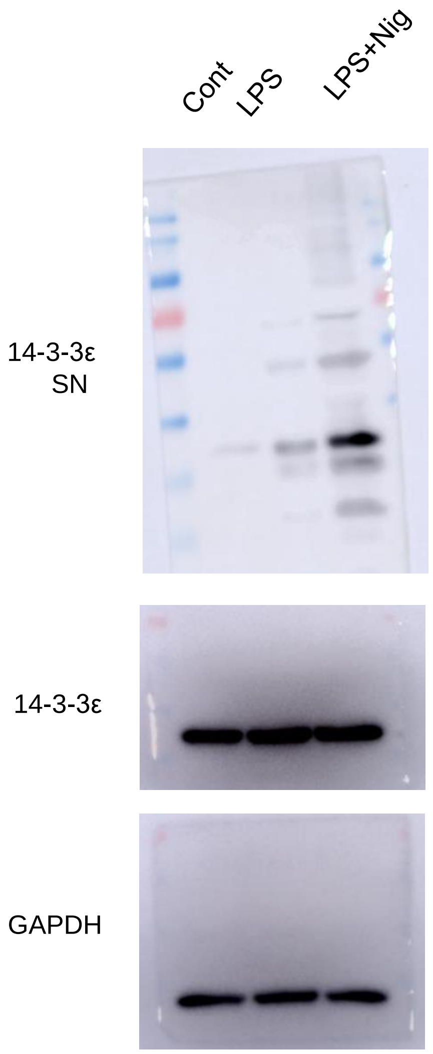

Figure S3A

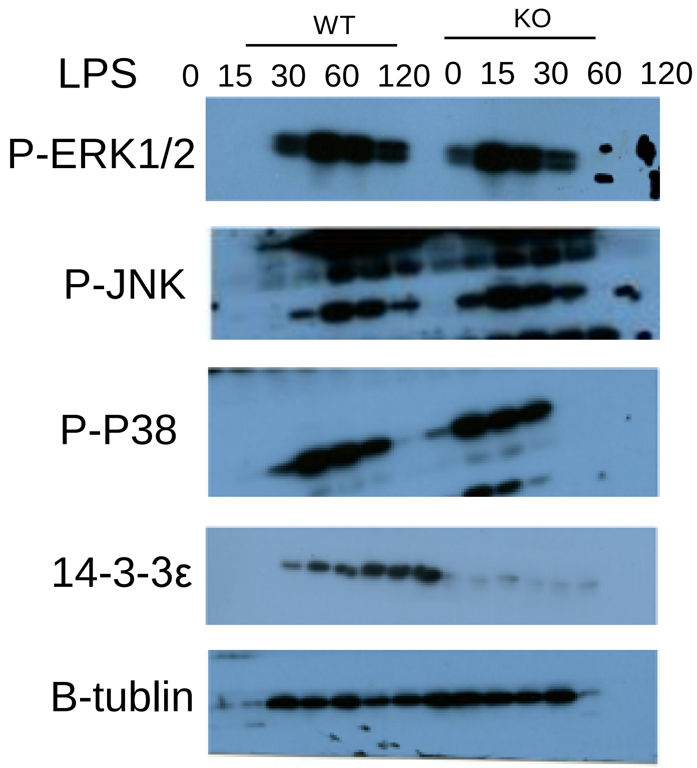

Figure S3C

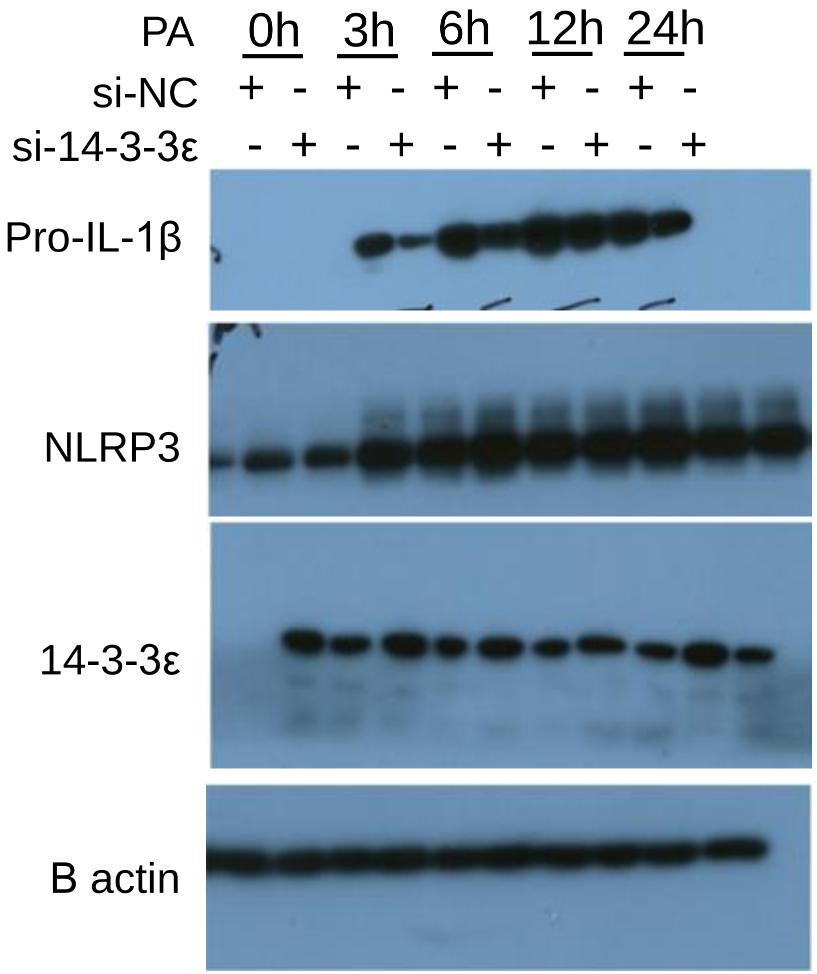

Figure S3D

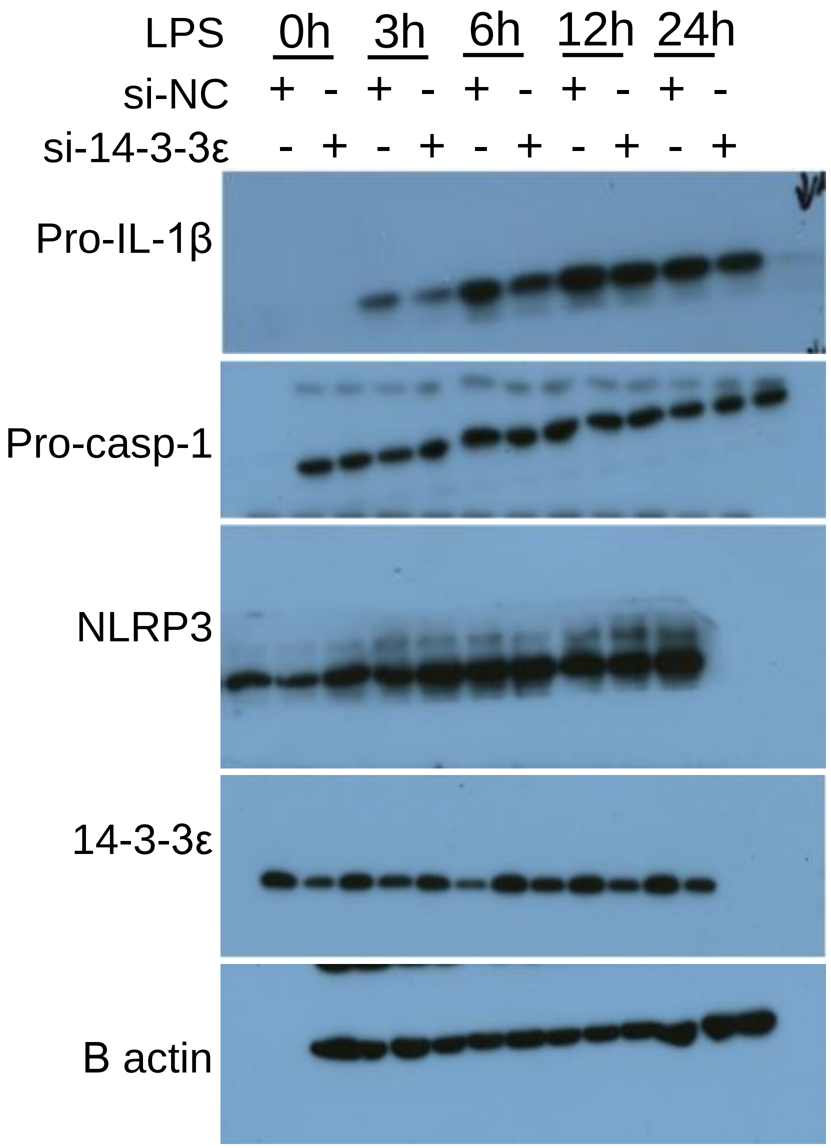

Figure S3E

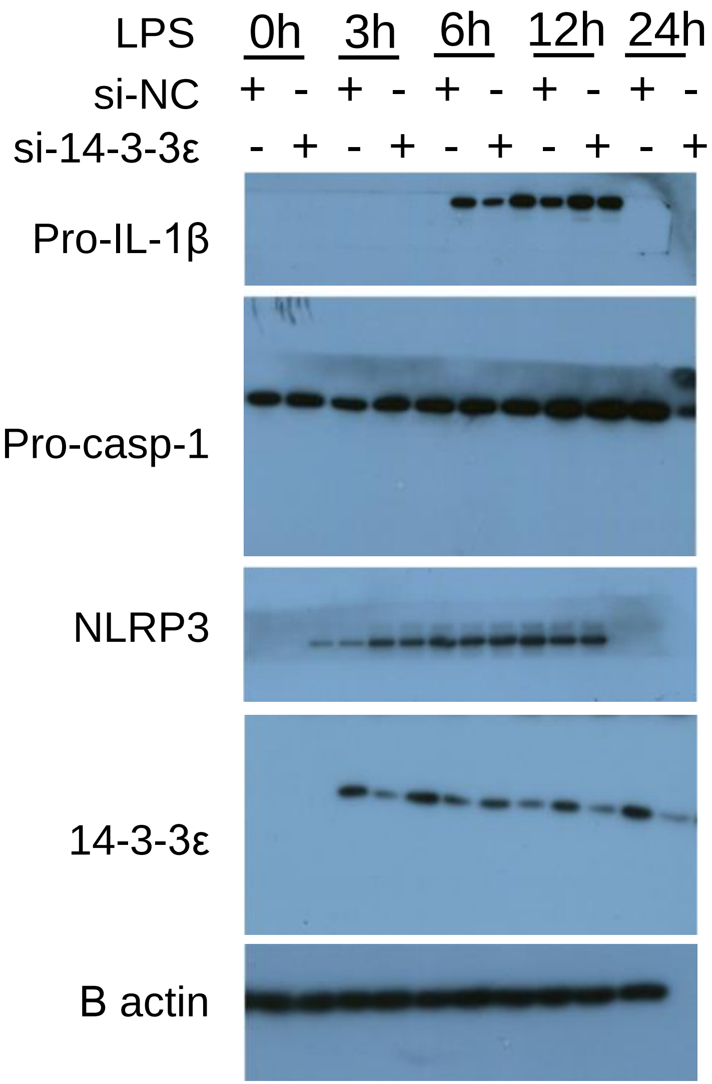

Figure S3F

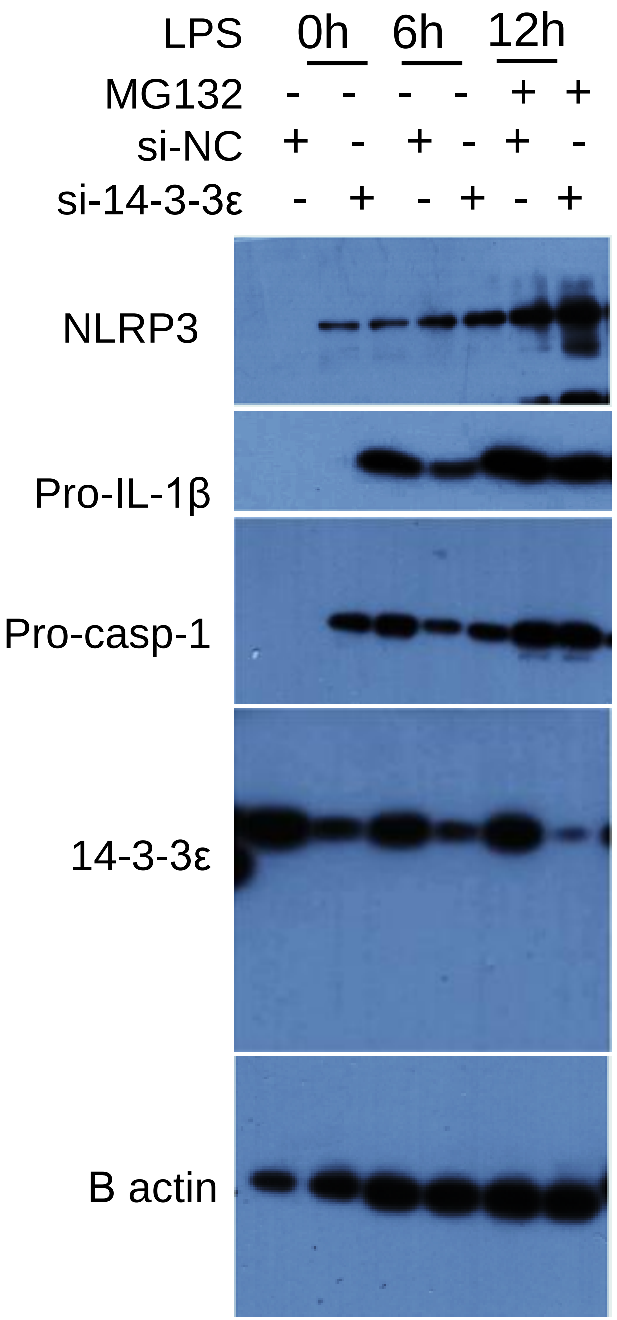

Figure S3F

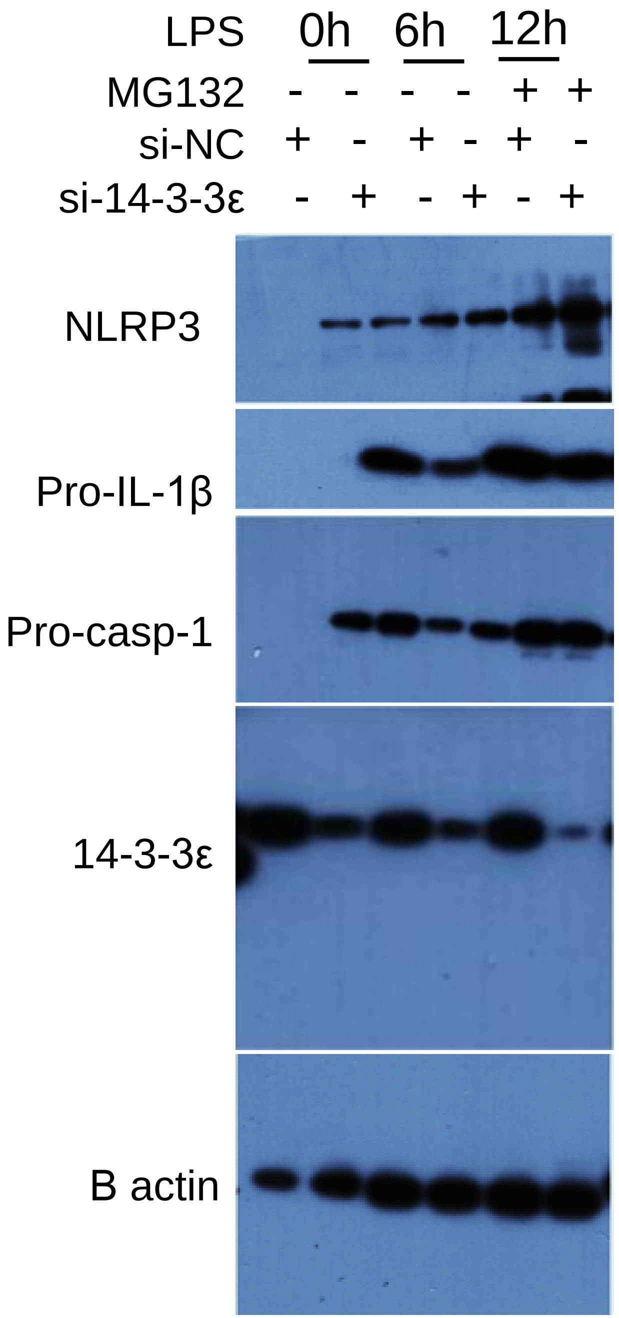

Figure S3G

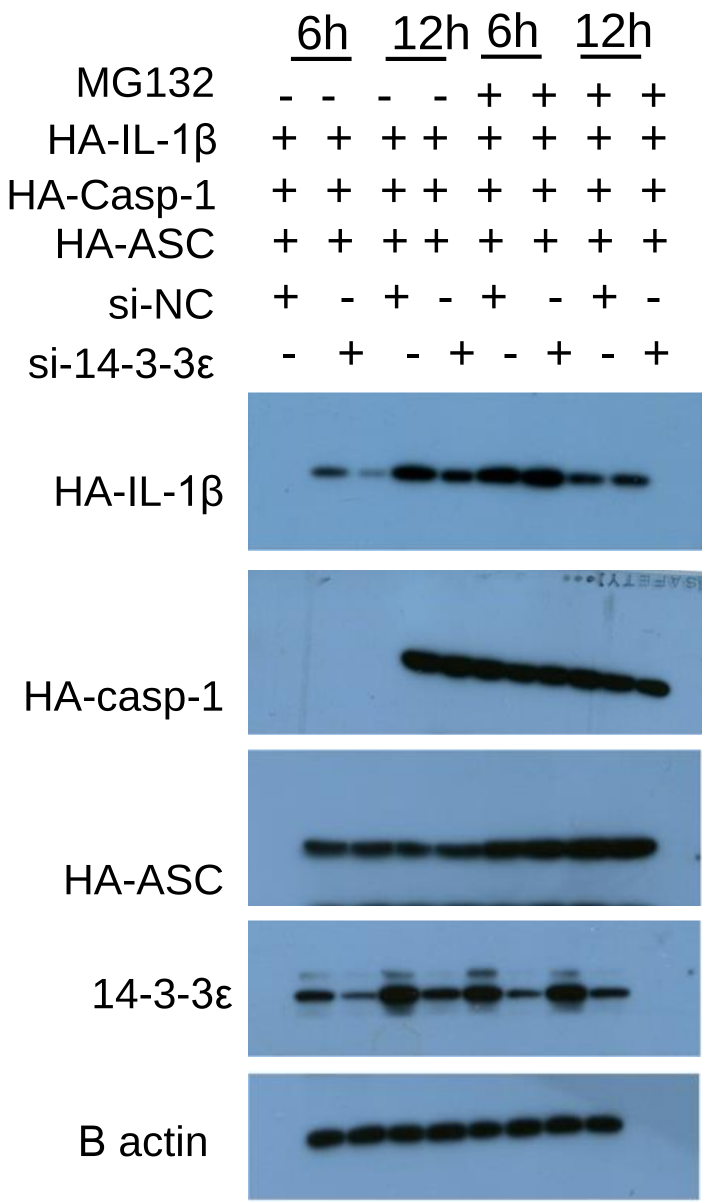

Figure S4A

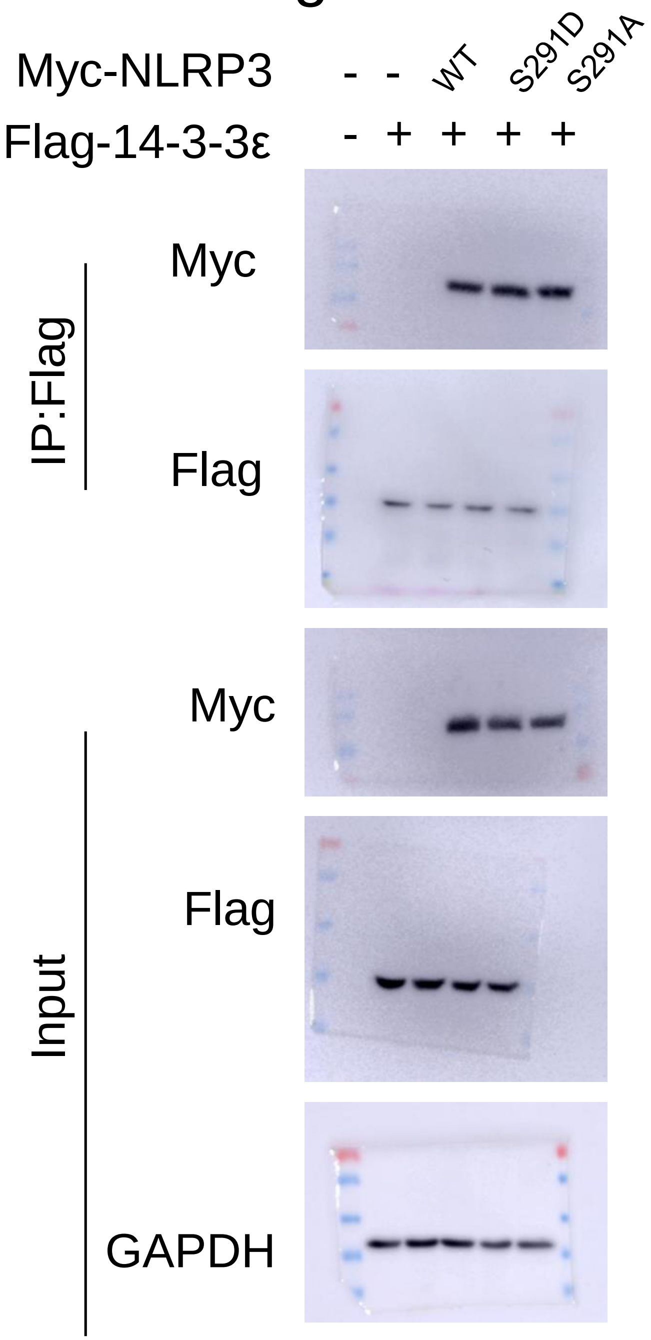

Figure S9C

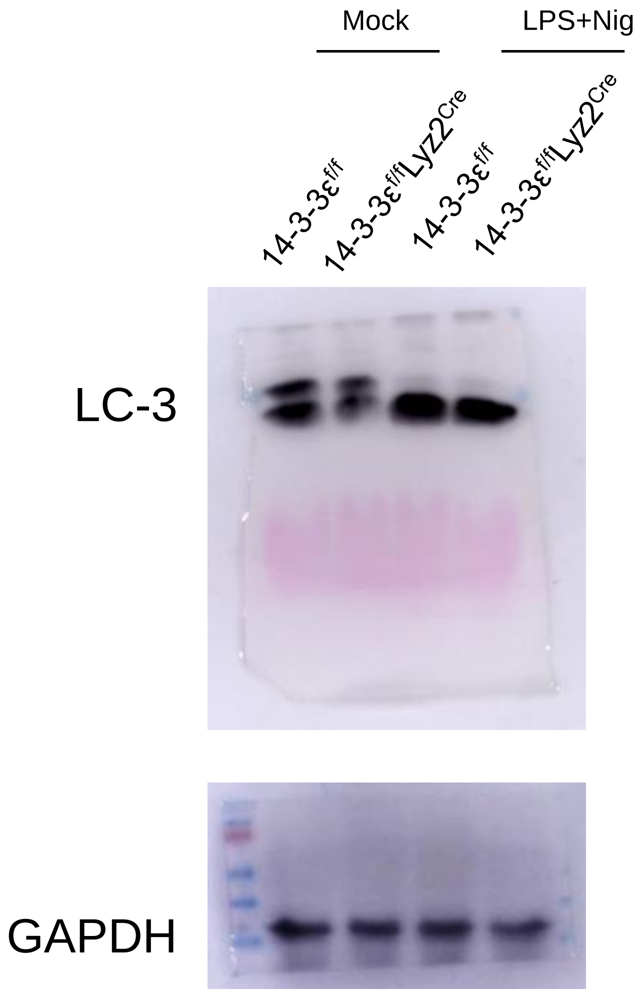

Supplement: Unedited blot and gel images [file jciinsight-11-192970-s211.pdf]
